# Adaptation of A-to-I RNA editing in *Drosophila*

**DOI:** 10.1371/journal.pgen.1006648

**Published:** 2017-03-10

**Authors:** Yuange Duan, Shengqian Dou, Shiqi Luo, Hong Zhang, Jian Lu

**Affiliations:** 1 State Key Laboratory of Protein and Plant Gene Research, Center for Bioinformatics, School of Life Sciences & Peking-Tsinghua Center for Life Sciences, Peking University, Beijing, China; 2 Academy for Advanced Interdisciplinary Studies, Peking University, Beijing, China; University of Michigan, UNITED STATES

## Abstract

Adenosine-to-inosine (A-to-I) editing is hypothesized to facilitate adaptive evolution by expanding proteomic diversity through an epigenetic approach. However, it is challenging to provide evidences to support this hypothesis at the whole editome level. In this study, we systematically characterized 2,114 A-to-I RNA editing sites in female and male brains of *D*. *melanogaster*, and nearly half of these sites had events evolutionarily conserved across *Drosophila* species. We detected strong signatures of positive selection on the nonsynonymous editing sites in *Drosophila* brains, and the beneficial editing sites were significantly enriched in genes related to chemical and electrical neurotransmission. The signal of adaptation was even more pronounced for the editing sites located in X chromosome or for those commonly observed across *Drosophila* species. We identified a set of gene candidates (termed “PSEB” genes) that had nonsynonymous editing events favored by natural selection. We presented evidence that editing preferentially increased mutation sequence space of evolutionarily conserved genes, which supported the adaptive evolution hypothesis of editing. We found prevalent nonsynonymous editing sites that were favored by natural selection in female and male adults from five strains of *D*. *melanogaster*. We showed that temperature played a more important role than gender effect in shaping the editing levels, although the effect of temperature is relatively weaker compared to that of species effect. We also explored the relevant factors that shape the selective patterns of the global editomes. Altogether we demonstrated that abundant nonsynonymous editing sites in *Drosophila* brains were adaptive and maintained by natural selection during evolution. Our results shed new light on the evolutionary principles and functional consequences of RNA editing.

## Author summary

Adenosine-to-inosine (A-to-I) RNA editing is an evolutionarily conserved mechanism that alters RNA sequences at the co-transcriptional or post-transcriptional level. RNA editing is hypothesized to facilitate adaptation in that it expands the transcriptomic and proteomic diversity. However, evidence for adaptation of RNA editing at the whole editome level is still lacking. In this study we systematically identified A-to-I RNA editing sites in female and male brains of three *Drosophila* species at different temperatures. With evolutionary analysis from different perspectives, we provide lines of evidence to demonstrate that the nonsynonymous editing sites in *Drosophila* brains are generally adaptive. The signals of adaptation for the editing sites are significantly enriched in genes related to chemical and electrical neurotransmission. We show that the RNA editing events might interplay with gene expression plasticity in temperature stress responses. Furthermore, we demonstrated that the expression level of *Adar*, together with the expression profiles of a set of genes that have editing sites favored by natural selection, were important in shaping the overall selective patterns of the global editomes at different developmental stages (or tissues) of *D*. *melanogaster*. Altogether our results support the hypothesis that A-to-I editing provides a driving force for adaptive evolution in *Drosophila* from different aspects.

## Introduction

Genomic mutations are the major sources for phenotypic changes and adaptation [1–4]. In diploid multicellular organisms, a nonsynonymous DNA mutation (a mutation that alters the amino acid sequence of a protein) will permanently affect the protein products in all the cells (soma or germline) that express the mutant allele. The “all-or-none” property of DNA mutations might incur pleiotropic effects that are antagonistic among cell types, tissues, developmental stages, sexes, or other aspects of life history [5–7], which would constrain the available genetic diversity for a species. Given the prevalence of pleiotropic effects in the genomes [8–10], the sequence space might be inaccessible to many mutations, which potentially slows down the rate of phenotypic evolution and adaptation [11]. However, the transcriptomic or proteomic diversity limited by mutation sequence space could be expanded by the alteration of RNA sequences in an epigenetic approach, such as RNA editing, which was hypothesized to facilitate adaptation [12–14]. In addition, RNA editing has the advantage to quickly respond to environmental stress and adjust the activity of final protein products accordingly [15, 16].

RNA editing is an evolutionarily conserved mechanism that alters RNA sequences at the co-transcriptional or post-transcriptional level [13, 17–23]. Among various RNA editing systems in animals, the base substitution from adenosine (A) to inosine (I), termed A-to-I editing, is the most common form [13, 20]. Due to the high level of structural similarity between inosine (I) and guanosine (G), the cellular machineries, such as ribosomes, spliceosomes or the microRNA ribonucleoprotein complex (miRNP), would recognize I as G during translation [13, 20, 24, 25], splicing [26–29], microRNA target recognition [30–32], or other RNA biological processes [14]. Therefore, A-to-I RNA editing usually produces a change similar to an A-to-G DNA change in particular tissues or developmental stages, which potentially increases phenotypic plasticity without the alteration of genomic sequences [13, 20, 24, 25]. The adenosine deaminase acting on RNA (ADAR) family are the enzymes that convert adenosine (A) to inosine (I) in pre-mRNAs [33–36]. Although multiple *Adar* genes are encoded in the genomes of mammals and worms, there is only one *Adar* locus in *Drosophila* [37, 38], which is predominately expressed in the nervous system [39]. The substrates of ADAR are usually double-stranded RNAs [34, 36, 40–42]. A-to-I editing plays essential roles in many biological processes [18, 19, 43–45], and the abolition of *Adar* in *D*. *melanogaster* severely affects its viability and behavior [33, 34, 46].

Previous studies have identified thousands of A-to-I editing sites in different developmental stages, adult heads or whole animals of *Drosophila* [47–52]. In addition, A-to-I editing has been extensively characterized in other organisms, such as humans [53–58], macaques [59, 60], mice [61], worms [62], and squids [63]. Despite these intriguing advances, only a few examples of the advantageous effects conferred by RNA editing have been demonstrated [13, 14, 20, 63]. For example, the A-to-I editing events in *Kv1* mRNA provide numerous adaptive amino acid changes that allow the octopus to adapt to extremely cold temperatures [64]. The functional consequences of the majority of A-to-I editing events, however, remain to be explored. In fact, comparative genomics has demonstrated that only a small fraction of the human A-to-I editing events were evolutionarily conserved [65–68]. Furthermore, it was nicely demonstrated that the editing events in primate coding regions were generally non-adaptive [60, 67, 68]. Nevertheless, the targets of RNA editing might have evolved rapidly across species because A-to-I editing in mammals predominantly occurs in repetitive sequences [53–55, 61, 65, 66], while the editing events in *Drosophila* are mainly located in coding regions of genes encoding neurotransmitters or ion channels [47–50, 69, 70]. Therefore, the evolutionary forces acting on A-to-I RNA editing might be different between *Drosophila* [50] and primates [60, 67, 68].

If A-to-I editing indeed facilitates adaptation by expanding proteomic diversity, we expect to observe predominant signals of adaptation in the editing sites. A recent study [50] reported that although signals of positive selection could be found in genes of the nervous system, the A-to-I RNA editing events were overall subject to purifying selection in *Drosophila*. Additionally, the overall effect of natural selection on the editome is different across *Drosophila* developmental stages [50]. Despite these intriguing discoveries, it still remains a mystery whether or not we can find evidence to show that the whole editome is overall adaptive. Specifically, we are interested in testing whether the nonsynonymous A-to-I editing events in *Drosophila* brains, the core component of the nervous system, are predominantly adaptive. Furthermore, several other fundamental questions on editing deserve to be further investigated: 1) Do editing sites preferentially increase sequence space of evolutionarily conserved genes? 2) Why does the global editome of different tissues or developmental stages show differential selective patterns? 3) How does temperature shape the global editomes? Answers to these questions will help understand the evolutionary principles and functional consequences of RNA editing.

In this study we addressed these questions by systematically sequencing the transcriptomes and deciphering A-to-I editing in the female and male brains of three *Drosophila* species at different temperatures. With evolutionary analysis from different perspectives, we provided lines of evidence to demonstrate that the nonsynonymous editing events in coding regions are generally adaptive in brains of *Drosophila*. Then we identified a set of gene candidates that had nonsynonymous editing events favored by natural selection. Overall our results demonstrated that abundant nonsynonymous editing events in *Drosophila* brains were adaptive and maintained by natural selection during evolution.

## Results

### The landscapes of brain editomes of *Drosophila melanogaster*

To comprehensively characterize the A-to-I editing landscapes in brains of *Drosophila*, we set out to dissect the brains of 1- to 5-day-old or 1- to 14-day-old female and male adults of the inbred ISO-1 strain of *Drosophila melanogaster* that were constantly raised at 25°C, or raised at 25°C and treated at 30°C for 14 hours or 48 hours (Table 1). Next we selected the poly(A)-tailed mRNAs, fragmented them, ligated the mRNA fragments with adaptors, and deep sequenced the transcriptome of each brain sample (Materials and Methods). We obtained 13.9–21.6M reads mapped on the reference genome (see Table 1 and S1 Table for detailed statistics), and the median coverage on an exonic site in a library ranges from 5 to 9 reads (S1 Fig). As justified previously [71], the mRNA fragmentation library preparation procedure we employ minimizes the bias of non-uniform sequencing read coverage along mRNAs, which would reduce the bias in detecting editing in 3' ends of mRNAs. It is a challenging task to reliably distinguish the A-to-I editing events from SNPs [72–76], therefore, the ISO-1 strain used in this study, which was inbred and sequenced to assemble the reference genome of *D*. *melanogaster* [77], enables us to detect DNA-RNA differences with high accuracy and sensitivity.

**Table 1:** A-to-I editing sites in female and male brains of *D*. *melanogaster*.

Library	Gender	Age (day)	Temp	Gene expressed	Mapped reads (M)	Edited genes	Sites	*N*	*S*	*N/S* ratio (95% CI)	*P* value	*α* (95% CI)
B1	F	1–14	25°C	9,770	20.8	383	1,157	393	82	4.79 (3.85, 6.20)	0.031	0.21 (0.01, 0.39)
B2	F	1–5	25°C	10,019	21.6	295	801	300	48	6.25 (4.70, 8.67)	4.9×10^−4^	0.39 (0.19, 0.56)
B3	F	1–5	30°C, 14 h	9,982	13.9	303	842	340	58	5.86 (4.53, 8.05)	9.2×10^−4^	0.35 (0.16, 0.53)
B4	F	1–5	30°C, 48 h	9,900	15.1	307	777	330	53	6.23 (4.80, 8.58)	2.9×10^−4^	0.39 (0.19, 0.56)
B5	M	1–14	25°C	9,968	16.6	419	1,297	448	85	5.27 (4.23, 6.84)	2.7×10^−3^	0.28 (0.10, 0.43)
B6	M	1–5	25°C	10,304	16.2	341	974	364	59	6.17 (4.72, 8.40)	1.8×10^−4^	0.38 (0.21, 0.54)
B7	M	1–5	30°C, 14 h	11,131	16.4	354	1,106	426	79	5.39 (4.26, 6.89)	2.0×10^−3^	0.29 (0.11, 0.46)
B8	M	1–5	30°C, 48 h	9,934	15.0	366	1,042	403	69	5.84 (4.62, 7.74)	3.7×10^−4^	0.35 (0.18, 0.51)
Total events						580	2,114	678	144	4.71 (3.95, 5.68)	0.01	0.19 (0.04, 0.33)

Totally 2,114 high-confidence editing sites were identified. In each library (*k*), we report the number of high-confidence sites that meet the following criteria: 1) editing level > 0.01; 2) mRNA-seq coverage ≥ 5, 3) edited G alleles ≥ 2, and 4) *P*_*k*_*(E*_*0*_*)* < 0.0001.

F: female; M: male. *N*: nonsynonymous change when edited; *S*: synonymous change when edited.

*P*: *P* value of the observed *N/S* ratio compared to the expected *N/S* ratio under neutral evolution.

*α*: the proportion of *N* sites that are adaptive with the formula 1- (*N/S)*_expected_*/* (*N/S)*_observed._

25°C: the flies were constantly raised at 25°C.

30°C, 14 h: the flies were raised at 25°C and treated at 30°C for 14 hours.

30°C, 48 h: the flies were raised at 25°C and treated at 30°C for 48 hours.

**Fig 1:**
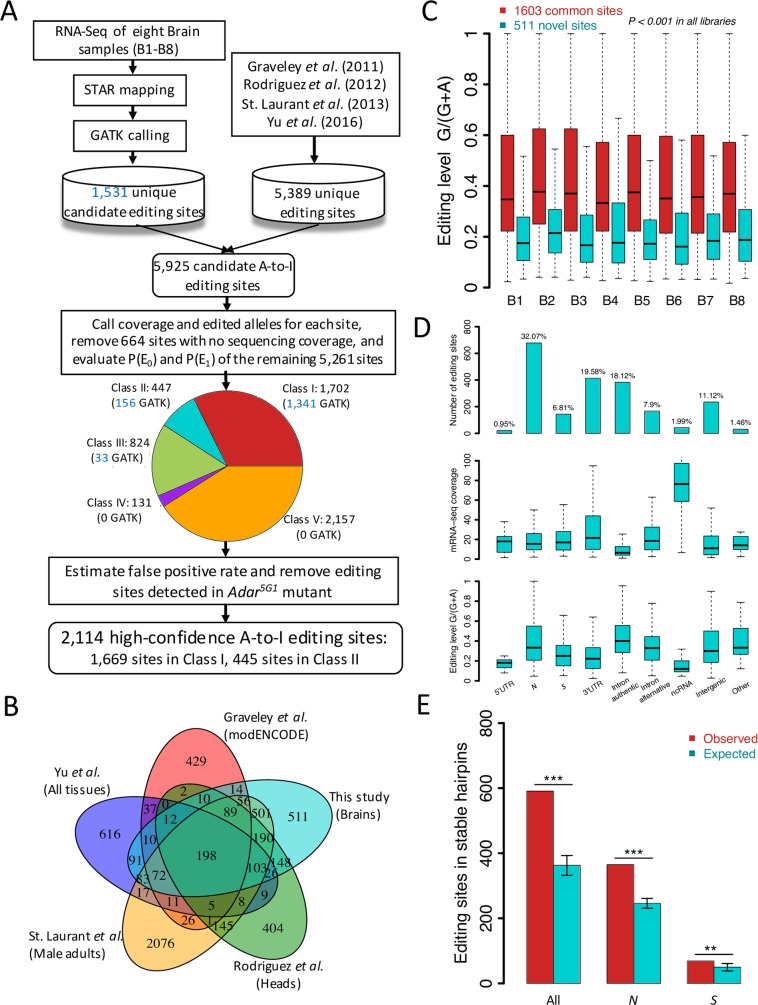
The landscape of A-to-I editomes in *D*. *melanogaster*. (A) A flowchart of A-to-I editing detection in brains of *D*. *melanogaster*. Editing sites are classified into five distinct classes based on the decreasing confidence of editing, sequencing coverage, and the number of libraries in which the editing events are detected. (B) Overlaps of the editing sites identified in this study and previous studies. (C) Boxplots of the editing levels of the common and novel sites in each brain library (*P* < 0.001 in each brain library, KS tests). (D) A summary of the editing sites with respect to their functional annotations. The numbers of high-confidence editing sites in each functional category in *D*. *melanogaster* are given in the top panel, and the proportion of editing sites is presented above the bars. mRNA-seq coverage (middle) and editing level (bottom) of editing sites in each category are also shown. For a site, the median value of coverage and editing level across all the libraries (if applicable) is used for the boxplots. (E) The observed numbers of editing sites located in stable hairpin structures and the expected numbers of sites (median and 95% confidence intervals) under randomness. ***, *P*< 0.001 revealed by simulations.

We employed a two-step strategy to identify editing sites in the brains of *D*. *melanogaster* (Fig 1A). First, we used the GATK RNA-Seq variant calling pipeline [78] to identify the candidate A-to-I editing sites in each brain library (*i*.*e*., the A-to-G differences in the final sequencing results). We identified 1,531 unique sites with A-to-G DNA-RNA differences in these brain libraries, and such differences accounted for 81.5% (with a standard error of 0.97%) of all the differences detected by the GATK pipeline in each library (S2A Fig). In contrast, the proportion of A-to-G DNA differences (reference vs. alternative allele) was only 9.9% out of all the mutations (S2B Fig) in the global populations of *D*. *melanogaster* [79]. This comparison justified the reliability and accuracy of our procedures in defining the candidate A-to-I editing sites. Second, we retrieved a total of 5,389 editing sites characterized in *D*. *melanogaster* in previous studies (972 in Graveley *et al*. [47], 1,350 in Rodriguez *et al*. [49], 3,581 in St. Laurant *et al*. [48], and 1,298 in Yu *et al*. [50]). Altogether, we obtained 5,925 unique candidate sites (986 sites overlapped between GATK and the four previous studies, S2 Table). For each candidate site in a brain library *k*, we calculate the probability that the A-to-G difference (if detected) is caused by editing with *P*_*k*_(*E*_1_) = 1 − *P*_*k*_(*E*_0_), where *P*_*k*_(*E*_0_) is the probability that the difference is solely caused by sequencing error (*ε*), by incorporating the sequencing coverage (*C*_*k*_) and the number of G allele (*L*_*k*_) at that site. Next, we utilize the multiple-sample information and calculate the joint probability that this site is edited in at least one library, *P*(*E*_1_) = 1 − *P*(*E*_0_), where *P*(*E*_0_) is the probability that the A-to-G differences observed in that site across all the applicable libraries are entirely caused by sequencing errors (Materials and Methods).

Among the 5,925 candidate sites, we did not detect expression of 664 sites in any brain library. For the remaining 5,261 expressed sites, we divided them into five exclusive classes with decreasing confidence based on *P*(*E*_1_), sequencing coverage, and the number of libraries in which the editing events were detected. Briefly, Class I (1,702 sites) were defined with the following criteria: 1) at FDR of 0.001, 2) the maximum sequencing coverage across all the libraries (*C*_*max*_) ≥ 10, 3) the total coverage across all the libraries (*C*_*total*_) ≥ 40, and 4) editing was detected in at least two libraries. Among the remaining sites that had editing events detected in at least two libraries, we defined Class II (447 editing sites) with these criteria: 1) at FDR of 0.01, 2) *C*_*max*_ ≥ 5 and 3) *C*_*total*_ ≥ 16 (we also employed other cutoffs to define editing sites in Class I and II, and obtained results not very different from the results reported here; see S3 Table for details). 824 sites do not meet the aforementioned two criteria but have *P*(*E*_1_) > 0.99, which suggests they might also be edited, although with lower confidence in brains of *D*. *melanogaster* (Class III). Moreover, 131 sites have editing detected in at least one library but have *P*(*E*_1_) ≤ 0.99 (Class IV). Notably, we detected mRNA-Seq reads covering the remaining 2,157 candidate sites but none of them has editing events detected (Class V). The detailed information about these sites is presented in S2 Table.

It is not surprising that the sequencing coverage decreases in the order of Class I, II, and III in each library [the median coverage in each library is 17.4±1.4 (mean ± s.e. throughout this study), 3.87±0.35 and 1±0 raw reads, respectively; S3A Fig]. Interestingly, although the sites in Class II have significantly lower coverage compared to sites in Class I (*P* < 0.05 in each library, Kolmogorov-Smirnov tests), the editing levels are even significantly higher in Class II than in Class I (the median editing level in each library is 0.24±0.02 vs. 0.16±0.01 in Class II vs. I, S3B Fig). From another perspective, 45.5% of the Class I sites were edited in all the eight brain libraries, meanwhile, only 8.5% of the Class II sites were edited in all the eight brain libraries (*P* < 0.01, Fisher’s exact test; S3C Fig). Compared to Class I and II, sites in Class III have both lower coverage and editing levels (S3A and S3B Fig). Sites in Class IV are extremely lowly edited and sites in Class V do not have any editing event detected in our samples; however, these two classes do not have the lowest sequencing coverage compared to the other three classes (the median coverage in a library is 18.6±1.1 and 6.1±0.35 for Class IV and V respectively, S3A Fig), suggesting they might have negligible editing in brains of *D*. *melanogaster*. To estimate the false positive rates of the editing sites in each class, we analyzed the RNA-Seq datasets from paired samples of wild-type strain *w*^*1118*^ vs. *Adar*^*5G1*^ mutant of *D*. *melanogaster* as conducted previously [50, 51]. We found 1,145, 161, and 103 editing sites in Class I, II, and III respectively that have editing events detected in *w*^*1118*^ heads, and correspondingly, 33, 2, and 7 of these sites were detected in the heads of *Adar*^*5G1*^ mutant, yielding a false positive rate of 2.88%, 1.24% and 6.80% for Class I, II, and III, respectively. Therefore, the sites in Class I and II captured the editing events in brains of *D*. *melanogaster* with high accuracy, and Class III sites were not considered in the down-stream analysis due to the high positive rate.

For the sites in Class I and II, we identified 1,630 (1,243 in Class I and 387 in Class II) sites overlapped with previous studies [47–50], and 519 sites (459 in Class I and 60 in Class II) are novel in this study (S2 Table). It is not uncommon that many editing sites are not overlapping between studies in *Drosophila* [47–50]: on average 30.4±3.6% of the editing sites are shared in pairwise comparisons (ranging from 12.8% to 54.7%, S4 Table); and we observed comparable proportions of shared sites between our study and the previous ones: 21.8%, 36.7%, 61.1% and 28.2% of the Class I+II sites in our study are overlapping with Graveley *et al*. [47], Rodriguez *et al*. [49], St. Laurant *et al*. [48], and Yu *et al*. [50], respectively (S4 Table). Importantly, when we pooled Class I and II together, we found the novel sites have comparable false positive rates as the common ones in the *w*^*1118*^ vs. *Adar*^*5G1*^ mutant analysis (8/242 = 3.31% vs. 27/1064 = 2.54% for the novel vs. common sites). Furthermore, 111 of the novel sites are annotated in Ramaswami *et al*. [52], which is independent from this study. Taken together, we identified 2,114 “high-confidence” editing sites after combining sites in Class I and II (35 sites that have A-to-G difference in *Adar*^*5G1*^ mutants were removed), including 1,603 (75.8%) sites overlapped with sites identified by previous studies [47–50] and 511 (24.2%) novel sites (Fig 1B). The novel sites have slightly higher sequencing coverage than the common sites in the brain libraries (the median coverage is 25.1±1.5 and 20.5±1.1 for the former and latter, respectively, *P* < 0.05 in each library, KS tests; S3D Fig), but generally lower editing levels (the median is 0.18±0.006 vs. 0.36±0.006, *P* < 10^−16^ in each library, KS tests; Fig 1C). Moreover, compared to the common editing sites, the novel sites are generally edited in fewer brain samples: 42.9% of the common ones were detected in all the eight brain libraries, while only 20.0% of the novel sites were detected in all the libraries (*P* < 10^−16^, Fisher’s exact test; S3E and S3F Fig). Altogether these results suggest that these novel editing sites are genuine but lowly edited in the brains, and were probably diluted in the samples of previous studies that were carried out in heads or whole flies [47–50].

Among the 2,114 high-confidence sites (Fig 1D), 235 (11.1%) are in intergenic regions, 42 (2.0%) are in ncRNAs, and 1,837 (86.9%) are in 517 protein-coding genes, including 20 (0.95%) in 5' UTRs, 550 (26.0%) in introns, 414 (19.6%) in 3' UTRs, 678 (32.1%) nonsynonymous (in CDS regions and causes amino acid changes when edited, abbreviated as *N* throughout this study), and 144 (6.8%) synonymous (in CDS regions but do not cause amino acid changes when edited, abbreviated as *S*), and one editing site (chr3R:18806029) that putatively disrupts the stop codon of *CG18208* (UAG>UGG). The detailed annotation in each library was presented in S5 Table. The gene ontology analysis revealed that the high-confidence exonic editing sites were significantly enriched in genes that encode transporters, synaptic vesicles or neurotransmitters (S6 Table and S7 Table for male and female brains, respectively; and the top 50 genes that had the largest number of editing sites were presented in S8 Table). For the exonic editing sites, the editing levels (averaged across libraries) decrease in the order of *N* (0.319±0.010), *S* (0.214±0.017), 3' UTRs (0.168±0.008), and 5' UTRs (0.133±0.020), with levels in *N* sites significantly higher than those in the other three categories in the brains of *D*. *melanogaster* (*P* < 0.001, KS test; Fig 1D), suggesting that high levels of nonsynonymous editing events are overall favorable. Among the 550 intronic editing sites, 167 of them might also be exonic due to alternative splicing (we only used annotations of the canonical transcript and some intronic sites in the canonical transcripts might be coding in the non-canonical transcripts), and the coverage and editing levels (0.336±0.013) are comparable to the *N* sites (Fig 1D). Interestingly, the remaining 383 authentic intronic sites generally have significantly lower coverage than the coding regions (Fig 1D), however, high editing levels in these sites (0.418±0.006) were observed, supporting previous results that editing is exerted co-transcriptionally [49].

### Features influencing genomic locations of A-to-I editing sites

We uncovered a tendency that A-to-I editing events were more readily detected in the genes with higher expression levels (or adenosine sites with higher mRNA-Seq coverage). In each brain sample, when we grouped the expressed genes into 20 bins with increasing expression levels (only genes with RPKM ≥ 1 were considered), we found a significant positive correlation between the editing density (hereafter defined as the number of edited out of the total adenosine sites) and the gene expression level (*P* < 0.001 in each library; S4A Fig). Similar patterns were observed if we grouped all the adenosine sites with increasing mRNA-Seq coverage in each sample (only sites ≥ 5X coverage were considered; S4B Fig). Analogous results were obtained if we weighted each editing site with its editing level (“level-weighted density of editing sites”, see S4A and S4B Fig).

Consistent with previous observations [47–49], we found the editing density was significantly increased from the 5' to 3' of pre-mRNAs. After dividing the adenosine sites (≥ 5X coverage) into 20 equal bins along their positions in pre-mRNAs, our meta-gene analysis indicated that the editing density in each bin was significantly positively correlated with the relative distance of that bin to the transcriptional start sites (*P* < 0.005 in each library; S4C Fig). Despite our experimental optimization, the poly(A) selection procedure still caused slightly increased coverage bias towards 3' ends of mRNAs (S4D Fig). However, we found the coverage difference between 5' and 3' of mRNAs was not the main cause of elevated editing density in the 3' ends of mRNAs with two analyses. First, in each library, we split each gene (RPKM ≥ 1) into two equal parts, calculated the RPKM values for each half-gene separately, ranked all the half-genes with increasing RPKM values, and grouped them into 20 bins. Next, in each bin, we combined the 5' and 3' half-genes independently and calculated the editing density in the 5' and 3' half. We found within each bin the editing density in the 3' half-genes are significantly higher than the 5' half genes (*P* < 0.05 in each library; paired *t* tests, S4E Fig). To further reduce the coverage variation within the half-genes, we ranked all the adenosine sites (≥ 5X) with increasing coverage and binned them into 20 groups, and in each group we calculated the editing density in the 5' (front) half and 3' (rear) half of pre-mRNAs independently. We also found the editing density were significantly higher for sites in the rear half compared to sites in the front half of pre-mRNAs (*P* < 0.001 in each library; paired *t* tests, S4F Fig). Taken together, the increasing editing density along mRNAs is not likely caused by detection bias, but more likely shaped by the recruitment of ADAR to the transcription elongation complex, as previous functional studies demonstrated [49, 80].

We predicted that 591 (50.1%) of the 1,179 exonic editing sites were located in stable local hairpin structures of mRNAs (Materials and Methods), such as *Adar* (S5A Fig), *rtp*, *DIP1*, *rdgA*, *CG43897*, and *CG42540* (editing events in these genes were verified with Sanger sequencing; S5B and S5C Fig). In contrast, we obtained only 363 exonic sites (332–393 sites within 95% CI) located in stable hairpin structures after comprehensively folding all the transcripts expressed in brains and randomly sampling the equal amounts of editing sites (Materials and Methods; Fig 1E). Similar results were obtained when we focused on the *N* or *S* editing sites individually (*P* < 0.002 in simulations for both cases; Fig 1E). In addition, we found 181 intronic editing sites located in stable hairpin structures when we folded the pre-mRNA sequences. Long-range pseudoknots are another class of RNA substrates recognized by ADAR [81]. By extensively folding the flanking sequences of the editing sites (see Methods for details), we inferred 260 (22.1%) exonic editing sites that were located outside stable hairpin structures but were located in stems of long-range pseudoknots in pre-mRNAs of genes, such as the 3' UTR of *Adar* (S5D Fig), *nrm*, *B52*, *nAchRbeta1*, *CG8034* and *roX1* (S6 Fig; the editing events in *nrm* were verified by Sanger sequencing of the cDNA and genomic DNA, S6B and S6C Fig). Taken together, our results systematically demonstrated that at least 874 (74.1%) of the exonic A-to-I editing sites in the brains of *D*. *melanogaster* were located in pre-mRNA regions that formed stable secondary structures. These results also well explain why the A-to-I editing sites are located in clusters, as commonly observed in previous studies [41, 47–49, 62]. By clustering the editing sites with distances smaller than 100 nucleotides, we identified a total of 1,320 editing sites that form 413 clusters in brains of *D*. *melanogaster* (S7 Fig), and unusually large editing clusters were frequently observed, such as in *NaCP60E* and *CaMKII* (the Sanger verification was presented in S8 Fig).

### The evolutionarily conserved A-to-I editing events in brains of three *Drosophila* species

**Fig 2:**
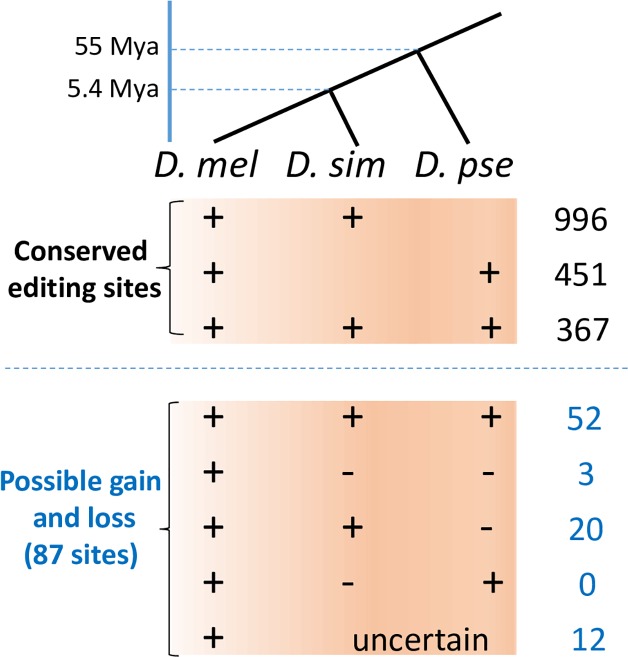
Conservation patterns of editing sites in brains of *D*. *melanogaster* and two other species. **“+”,** the high-confidence editing sites were reliably detected in a species (Top). Bottom: possible gain and loss patterns of 87 sites that have a minimal editing level of 0.05 in *D*. *melanogaster* and have at least 200 raw reads in both *D*. *simulans* and *D*. *pseudoobscura*. “-”, the orthologous site is not edited with high probability [joint *P(D*_*0*_*)* < 0.0002].

To characterize the A-to-I editing events that were evolutionarily conserved (*i*.*e*., commonly observed) across species, we deep sequenced the poly(A)-tailed transcriptomes of female and male brains of 1- to 5-day-old *D*. *simulans* and *D*. *pseudoobscura* that were accommodated at the same temperature conditions as *D*. *melanogaster* (six libraries for each species). The mapped reads range from 8.7–16.4M in each library of *D*. *simulans*, and 10.9–17.8M in *D*. *pseudoobscura* (Table 2, Table 3 and S1 Table for detailed information), and the median sequencing coverage on an exonic site in a library ranges from 5 to 9 reads in *D*. *simulans*, and ranges from 4 to 5 in *D*. *pseudoobscura* (S1 Fig). *D*. *simulans* diverged from *D*. *melanogaster* ~5.4 million years ago (Fig 2) while *D*. *pseudoobscura* diverged from *D*. *melanogaster* approximately 55 million years ago [82]. Comparing A-to-I editing across these three species will help us understand the role of natural selection in shaping the brain editomes during evolution. To exclude SNPs in the RNA editing characterization, we also deep sequenced the genomic DNA of the same strain of *D*. *simulans* (the median coverage per site is 46, totally 313,133 SNPs, S9A Fig) and *D*. *pseudoobscura* (the median coverage per site is 47, totally 489,828 SNPs, S9B Fig) and masked all the SNPs (Materials and Methods).

**Table 2:** The editing sites with events observed in brain of *D*. *simulans* and the matched brain sample of *D*. *melanogaster*.

Library	Gender	Age (day)	Temp	Mapped reads (M)	Edited genes	Sites	*N*	*S*	*N/S* ratio (95% CI)	*P* value	*α* (95% CI)
S2	F	1–5	25°C	8.68	158	275 (34.3%)	153	20	7.65 (5.18, 13.4)	4.4×10^−3^	0.45 (0.16, 0.69)
S3	F	1–5	30°C, 14 h	9.07	169	304 (36.1%)	192	22	8.73 (5.90, 14.3)	2.5×10^−4^	0.52 (0.29, 0.71)
S4	F	1–5	30°C, 48 h	13.5	197	342 (44%)	199	28	7.11 (4.97, 11.0)	3.5×10^−3^	0.41 (0.16, 0.62)
S6	M	1–5	25°C	11.8	216	420 (43.1%)	231	32	7.22 (5.12, 11.0)	1.4×10^−3^	0.42 (0.18, 0.62)
S7	M	1–5	30°C, 14 h	16.4	230	469 (42.4%)	252	32	7.88 (5.60, 11.9)	1.9×10^−4^	0.47 (0.25, 0.65)
S8	M	1–5	30°C, 48 h	15.3	209	414 (39.7%)	240	29	8.28 (5.90, 13.2)	1.1×10^−4^	0.50 (0.29, 0.66)
Total sites					333	996 (47.1%)	494	86	5.74 (4.63, 7.29)	2.9×10^−3^	0.27 (0.10, 0.43)

Totally 996 sites with conserved events were identified. We report the number of sites that meet the following criteria: 1) editing level > 0.01; 2) mRNA-seq coverage ≥ 5, 3) edited G alleles ≥ 2, and 4) *P*_*k*_*(E*_*0*_*)* < 0.0001 in each library (*k*) of *D*. *simulans*.

“Edited genes”: genes that have high-confidence editing sites in brains of both *D*. *simulans* and the matched sample of *D*. *melanogaster*.

“Sites”: sites in brains of both *D*. *simulans* and the matched sample of *D*. *melanogaster* are reported. The percentages of the conserved sites out of the sites in each matched brain library of *D*. *melanogaster* were presented in the parenthesis.

**Table 3:** The editing sites with events observed in brain of *D*. *pseudoobscura* and the matched brain sample of *D*. *melanogaster*.

Library	Gender	Age (day)	Temp	Mapped reads (M)	Edited genes	Sites	*N*	*S*	*N/S* ratio (95% CI)	*P* value	*α* (95% CI)
P2	F	1–5	25°C	10.9	105	179 (22.3%)	142	13	10.9 (6.75, 21.1)	0.08	0.35 (-0.05, 0.66)
P3	F	1–5	30°C, 14 h	11.7	112	188 (22.3%)	147	14	10.5 (6.67, 22.0)	0.10	0.32 (-0.12, 0.68)
P4	F	1–5	30°C, 48 h	13.7	114	179 (23%)	143	14	10.2 (6.48, 21.4)	0.12	0.30 (-0.16, 0.67)
P6	M	1–5	25°C	15.8	125	235 (24.1%)	181	17	10.7 (6.92, 18.8)	0.06	0.33 (-0.03, 0.62)
P7	M	1–5	30°C, 14 h	17.8	120	253 (22.9%)	190	22	8.64 (5.84, 14.1)	0.23	0.18 (-0.22, 0.50)
P8	M	1–5	30°C, 48 h	15.8	125	237 (22.7%)	181	19	9.53 (6.41, 17.2)	0.13	0.25 (-0.11, 0.59)
Total events					159	451 (21.3%)	325	48	6.77 (5.11, 9.36)	0.66	-0.05 (-0.39, 0.24)

Totally 451 sites with conserved events were identified. We report the number of sites that meet the following criteria: 1) editing level > 0.01; 2) mRNA-seq coverage ≥ 5, 3) edited G alleles ≥ 2, and 4) *P*_*k*_*(E*_*0*_*)* < 0.0001 in each library (*k*) of *D*. *pseudoobscura*.

“Edited genes”: genes that have high-confidence editing sites detected in brains of both *D*. *pseudoobscura* and the matched sample of *D*. *melanogaster*.

“Sites”: sites in brains of both *D*. *pseudoobscura* and the matched sample of *D*. *melanogaster* are reported. The percentages of the conserved sites out of the sites in each matched brain library of *D*. *melanogaster* were presented in the parenthesis.

For each high-confidence editing site in brains of *D*. *melanogaster*, we employed two complementary approaches to search for the orthologous sites in *D*. *simulans* and *D*. *pseudoobscura*. First, we used liftOver [83] to convert the genomic coordinates of the orthologous sites between *D*. *melanogaster* and *D*. *simulans*, or between *D*. *melanogaster* and *D*. *pseudoobscura*, based on the pairwise genome alignments as previously conducted [51] (termed “g_align” approach, Materials and Methods). Second, we parsed out the genomic coordinates with the pairwise CDS alignments that were made based on the protein alignments between *D*. *melanogaster* and the other species (“c_align” approach). We pooled orthologous sites by the two approaches together. For each site in each species, we calculated the joint probability that this site is edited in at least one library *P*(*E*_1_). At FDR of 0.05, we identified 996 sites edited in *D*. *simulans* (S9 Table), and 451 sites edited in *D*. *pseudoobscura* (S10 Table), and 367 sites edited in both *D*. *simulans* and *D*. *pseudoobscura* (Fig 2). We present the editing sites evolutionarily conserved in the same gender under the same temperature conditions in *D*. *simulans* (Table 2 and S11 Table) and *D*. *pseudoobscura* (Table 3 and S12 Table). For the editing sites we characterized in the brains of *D*. *melanogaster*, 34.3–44.0% of them have editing events detected in the matched samples of *D*. *simulans* (Table 2), and 22.3–24.1% of them have editing in the matched samples of *D*. *pseudoobscura* (Table 3). Note the proportion of editing sites in *D*. *melanogaster* that have editing events detected in brains of other species varies across libraries since we required the sites are edited in both paired samples. In general, with divergence increases, the level of conserved editing sites decreased, suggesting the editing events are evolutionary dynamic. Notably, for the 996 editing sites with conserved events in both *D*. *melanogaster* and *D*. *simulans*, and the 451 editing sites with conserved events in both *D*. *melanogaster* and *D*. *pseudoobscura*, we found 416 (41.8%) and 78 (17.3%) of them are located outside the coding regions, respectively (Tables 2 and 3), which is consistent with a recent study [84] and suggests a possible functional role for these sites, such as influencing alternative splicing [26–29], microRNA targeting [30–32], or other cellular processes related to RNAs [14].

Comparing the editing sites with conserved and non-conserved events revealed two interesting features. First, in each brain library, the editing levels are significantly higher in the sites with evolutionarily conserved editing events than in the remaining sites (the mean level in a library is 0.340±0.008 vs. 0.252 ± 0.009 in the *D*. *melanogaster/D*. *simulans* comparison, and 0.323±0.012 vs. 0.187±0.009 in the *D*. *melanogaster/D*. *pseudoobscura* comparison; *P* < 0.01 in each comparison, KS tests, S10 Fig). Second, the *N* sites are significantly enriched in the editing sites that are evolutionarily conserved: 72.9% (494 out of 678) *N* sites compared to 35.0% (502 out of 1436) of the remaining sites that have evolutionarily conserved events between *D*. *melanogaster* and *D*. *simulans* (*P* < 10^−10^, Fisher’s exact test), and 47.9% (325 out of 678) *N* sites compared to 8.77% (126 out of 1436) of the remaining sites that have evolutionarily conserved events between *D*. *melanogaster* and *D*. *pseudoobscura* (*P* < 10^−10^, Fisher’s exact test), suggesting the nonsynonymous editing events are generally maintained and regulated by different evolutionary forces compared to the other sites.

There are 84 editing sites that have editing events detected in both *D*. *melanogaster* and *D*. *pseudoobscura* but without editing events confidently identified in *D*. *simulans*. Nevertheless, this does not necessarily mean these sites are not edited in *D*. *simulans* (for 60 of these sites we did not find the orthologous sites in *D*. *simulans*, and for the 24 remaining sites, 10 of them have low level of editing but are undistinguishable from sequencing errors; S13 Table). Sampling bias frequently causes the sites with low expression or low editing levels to yield no editing signals in the sequencing libraries. Therefore, next we only focused on the sites with high sequencing coverage to explore the possible gain and loss patterns of editing events. We obtained 87 editing sites that have minimal editing level of 0.05 in *D*. *melanogaster* and have at least 200 raw reads (across all the libraries) in both *D*. *simulans* and *D*. *pseudoobscura*. We found 52 sites with editing events reliably detected in all the three species. For each of the remaining 35 sites, in case no editing event was discovered at a site in a sample *m* in *D*. *simulans* (*or D*. *pseudoobscura*), we calculate *P*_*m*_(*D*_0_), the probability that this observation happens by sampling bias or because the editing signal was abolished by sequencing error (*ε*), given a depth of *C*_*m*_ and an assumed editing level *l*_*m*_ at that site (Materials and Methods). We assumed the orthologous sites in the other species have the same editing level as in *D*. *melanogaster* and calculated the joint probability *P*(*D*_0_) that a site was edited despite zero edited allele was detected in all the libraries. After correcting for multiple testing, at FDR of 0.05, we found 20 sites with editing present in both *D*. *melanogaster* and *D*. *simulans* but absent in *D*. *pseudoobscura*, and 3 sites with editing specifically present in *D*. *melanogaster*. The most parsimonious interpretation is that the brain editome in *Drosophila* is expanding during evolution (Fig 2). We did not find any convincing case that editing was detected in both *D*. *melanogaster* and *D*. *pseudoobscura* but was absent in *D*. *simulans*, suggesting that the established editing events, at least for the set we examined here, are well maintained by natural selection during evolution.

### Signals of adaptation in brain editomes of *Drosophila*

In contrast to previous observations that nonsynonymous editing events were generally non-adaptive in mammals [67, 68] and in *Drosophila* [50], our analysis revealed the nonsynonymous editing events in *Drosophila* brains were predominantly adaptive. The ratio of nonsynonymous (*N*) to synonymous (*S*) editing sites (*N/S*) in different brain samples of *D*. *melanogaster* ranges from 4.79 with 95% CI (3.85, 6.20) to 6.25 (4.70, 8.67), all of which is significantly higher than the ratio expected under neutrality (3.80) that was calculated similarly as previously described [67] (Materials and Methods; *P* < 0.03 in each library, Fisher’s exact tests; Table 1). In other words, in the brains of *D*. *melanogaster*, the rate of nonsynonymous A-to-I editing is significantly higher than the rate of synonymous editing. Given the observed and expected *N/S* ratios under neutrality (randomness), a conservative estimation is that 20.7% [with 95% CI (1.3%, 38.7%)] of the *N* sites in the brains of *D*. *melanogaster* might be adaptive (Table 1). Moreover, we obtained higher *N/S* ratios in each brain library when we increased the cutoff of editing level (S11 Fig). Our analysis is essentially the same as the classical *dN/dS* (the number of nonsynonymous changes *per* nonsynonymous site over the number of synonymous changes *per* synonymous site) test of DNA sequences in molecular evolution [85], and provides compelling evidence that the nonsynonymous editing events in *Drosophila* brains are overall beneficial and favored by natural selection.

We observed significantly negative correlations between the sequencing coverage (*C*) and editing level (*l*) in each brain library or when we pooled the library together (*P* < 10^−10^ in each case, S12 Fig). These patterns do not necessarily mean that lowly expressed sites have higher editing levels, but rather suggest that the sites of lower sequencing coverage have stronger sampling bias: editing events in such sites are either not detected or detected with over-estimated editing levels. Furthermore, although the *S* and *N* editing sites have comparable coverage (Fig 1D), the *S* sites are generally edited at lower levels compared to the *N* sites (Fig 1D). Therefore sampling bias would affect *S* sites more severe than the *N* sites, which potentially causes over-estimation of the *N/S* ratios in the above analysis. However, to what extent the *N/S* ratio is over-estimated due to sampling bias remains unclear. Our “joint probability method” in detecting editing across multiple libraries allows the editing sites with low coverage or with low editing levels in a single library to be efficiently identified with the aid of information from other libraries (such sites are usually filtered out if only based on information of a single library), and the full list of editing sites across libraries enable us to test whether and how our observed *N/S* ratios are affected by sampling bias. We conducted simulations with two different methods, both of which considered the observed distribution of sequencing coverage and editing levels among sites.

**Fig 3:**
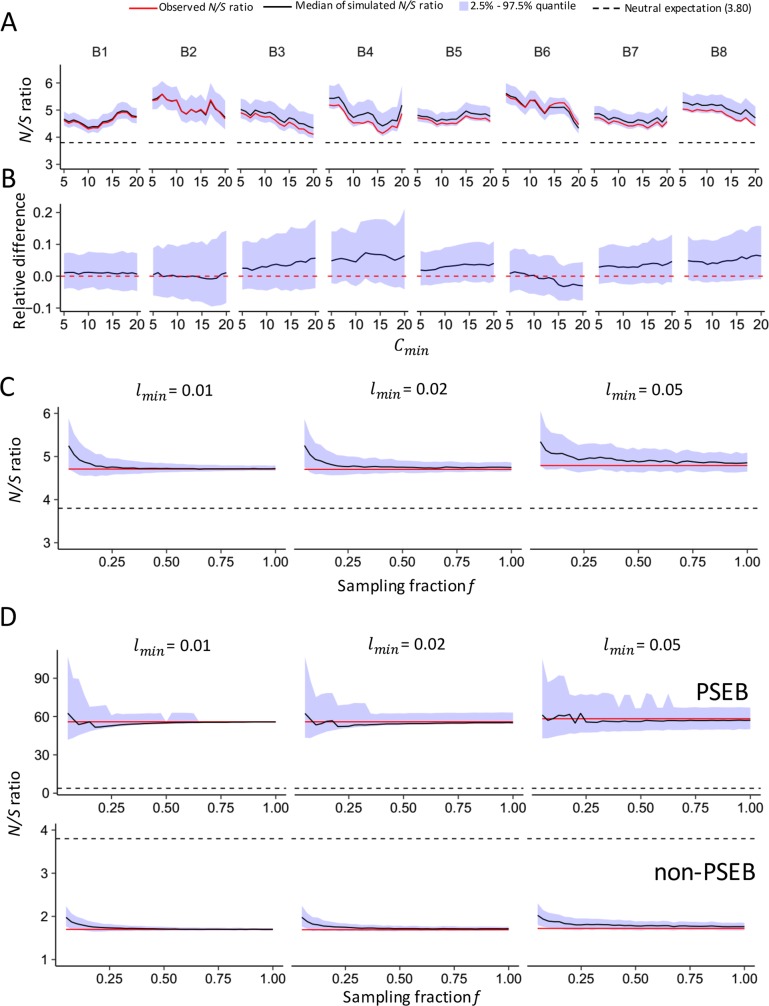
Evaluating the effect of detection bias on *N/S* ratio estimation. (A) The simulated and observed *N/S* ratios with increasing cutoffs of sequencing coverage (*C*_*min*_). The *x-*axis is the cutoff of coverage (*C*_*min*_) and the *y*-axis is the simulated (median in black, and the range from 2.5% to 97.5% quantile is in blue) and observed (red) *N/S* ratio. The *N/S* ratio under neutral evolution (3.80) is indicated with dashed lines. *l*_*min*_ is 0.02 here. (B) The relative difference of the simulated vs. the observed *N/S* ratio with increasing *C*_*min*_. Each plot is corresponding to the upper one in (A). (C) The simulated (median in black and the range from 2.5% to 97.5% quantile in blue) and observed (red) *N/S* ratios (the *y*-axis) when pooling all the brain libraries together and randomly sampling a fraction (*f*, from 0.05 to 1, the *x*-axis) of reads. Results with the cutoff of editing level, *l*_*min*_ = 0.01, 0.02 and 0.05 are presented. The *N/S* ratio under neutral expectation (3.80) is indicated with dashed lines. (D) The simulations of the editing sites in PSEB (upper panel) and non-PSEB genes (lower panel) as in (C).

In the first method, we focused on all the high-confidence sites present in a library that had sequencing coverage *C* ≥ *C*_*min*_ and editing level *l* ≥ *l*_*min*_. In each round of simulation, for a site *j* that had an observed depth *C*_*j*_, we randomly sampled *C*_*j*_ reads (with replacement) from all the sequenced reads covering that site and calculated the simulated editing level *l*_*sj*_ with the obtained reads of the edited allele, we counted the site only if *l*_*sj*_ ≥ *l*_*min*_, and then we pooled all the counted sites together and calculated the *N/S* ratio for this round of simulation. For each brain library, we tried different combinations of *C*_*min*_ (ranging from 5 to 20, with *C*_*min*_ = 20 roughly accounting for 50% of all the editing sites in a library) and *l*_*min*_ (0.01, 0.02 and 0.05) values, and performed the simulations for 1,000 replicates. At different *l*_*min*_ cutoffs, both the observed and simulated *N/S* ratios were generally higher at lower *C*_*min*_ values; and the median simulated *N/S* ratio is usually higher than the observed one, but the extent of elevation is very modest, in most cases much smaller than 10% (see Fig 3A and 3B for all the eight brain libraries at *l*_*min*_ of 0.02, and S13 Fig for other *l*_*min*_ values). Similar results were obtained when we pooled all the libraries together and performed the simulations (S14 Fig). These results suggest that detection bias of editing sites would slightly increase the observed *N/S* ratio, however, the over-estimation caused by such a bias is very modest compared to the large difference between the observed and the expected *N/S* ratio under neutral evolution (Fig 3B). Importantly, the observed *N/S* ratio is significantly higher than the neutral expectation even after we adjusted the bias (S14 Table).

In the second method, we pooled all the eight libraries together and randomly sampled (with replacement) a fraction (*f*) out of the total reads, and after that, we calculated the *N/S* ratio for the sites that have simulated editing levels *l*_*sj*_ ≥ *l*_*min*_. We tried different combinations of *f* (from 0.05 to 1 with a step size of 0.025) and *l*_*min*_ (0.01, 0.02 and 0.05) values, and performed the simulations for 1000 replicates. In agreement with the first method, the simulated *N/S* ratios were higher at lower depth (smaller *f* values). For example, when *f* is set at 0.05, which is less than half the size of a library we sequenced, the *N/S* ratio would be elevated by roughly 10% due to sampling bias (Fig 3C). However, the simulated *N/S* ratios approached to the observed *N/S* ratio (calculated based on the pooled libraries) rapidly with increasing *f* (Fig 3C). In summary, the simulations revealed that *N/S* ratios tend to be overestimated at lower sequencing coverage, however, the degree of over-estimation was usually small given our sequencing depth. Our conclusion that the *N/S* ratios in the brain libraries are significantly higher than the neutral expectation is not affected by the possible detection bias.

**Fig 4:**
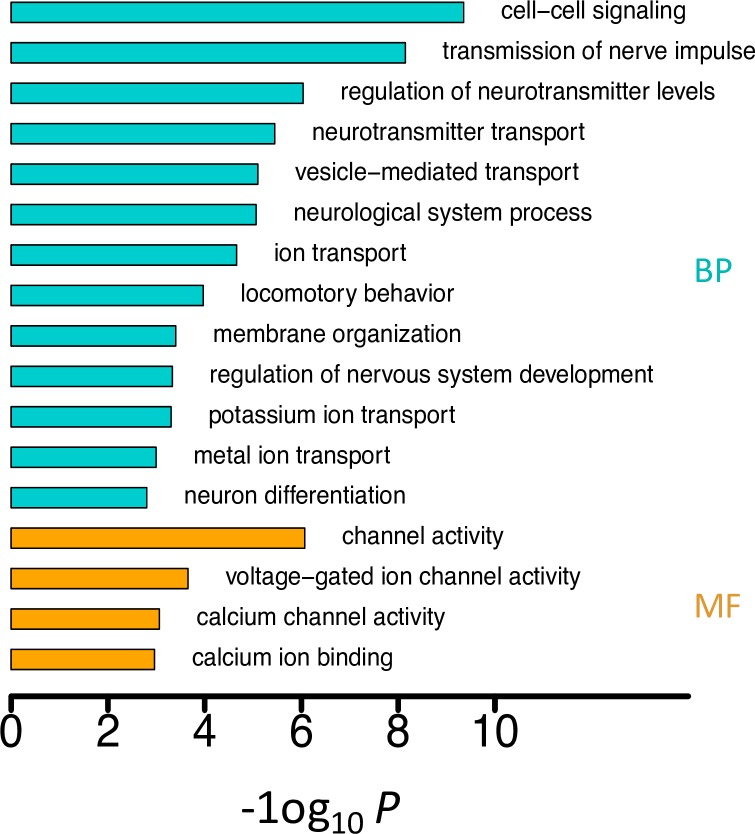
The Gene Ontology (GO) enrichment analysis on the PSEB genes. MF: molecular function; BP: biological process.

It is notable that several X-linked genes such as *cac*, *CG42492* and *Sh* harbor multiple *N* editing sites (12, 10 and 3 respectively) while very few *S* editing sites (1, 1, and 0 respectively). In fact, the *N/S* ratio is substantially higher for the X-linked than the autosomal genes in all the eight brain libraries of *D*. *melanogaster*, indicating the signal of adaptation is generally stronger for the X-linked genes in *Drosophila* brains (S15A Fig). This observation is essentially congruent with the fast-X evolution observed for nonsynonymous DNA mutations under positive selection, by which the X-linked advantageous effect is more readily manifested compared to the autosomal counterparts [86, 87]. Furthermore, we identified a set of brain-expressed gene candidates that had *N* editing sites favored by natural selection, which were termed “PSEB” (Positively Selected Editing in Brains) gene set (we required each gene to have a *N/S* editing ratio > 5; totally 223 genes met this criteria and 49 of them were X-linked; S15 Table). There are 683 high-confidence editing sites in the PSEB genes in brains of *D*. *melanogaster*, including 447 *N* and only 8 *S* sites, yielding a *N/S* ratio of 55.9. 80 (35.9%) of the PSEB genes are overlapping with the type III genes that have editing events positively selected by *Yu et*. *Al*. [50]. The *N* editing sites in the PSEB genes, which are significantly enriched in chemical and electrical neurotransmission pathways (Fig 4), are very likely the targets of positive selection. Notably, the expression levels of the PSEB genes are significantly higher than the non-PSEB genes (S16 Fig), which suggests that the higher *N/S* ratios in the PSEB genes are not likely caused by sampling bias of the *S* sites. Moreover, we ranked all the genes with editing events in each brain library with increasing expression level (RPKM) and equally divided those genes into “Highly” and “Lowly” expressed groups. In both groups, the *N/S* ratios are significantly higher for the editing sites in the PSEB compared to non-PSEB genes (S16 Table). Furthermore, we conducted random sampling simulations and confirmed that neither the higher *N/S* ratios in the PSEB genes nor lower *N/S* ratios in the non-PSEB genes was caused by detection bias (Fig 3D). Notably, the editing levels of the PSEB sites are higher than the remaining sites in all the brain libraries, and four of them are statistically significant (S17 Fig), which further suggests that editing in these sites are favored by natural selection.

We observed higher *N/S* ratios in the sites that have editing events commonly observed across *Drosophila* species, partially caused by the bias that synonymous adenosine sites are less constrained during evolution. However, after we contrasted the observed *N/S* ratios to the expected values (4.18) calculated from the evolutionarily conserved adenosine sites (Materials and Methods), we still detected signals of adaptation in the sites with conserved editing events between *D*. *melanogaster* and *D*. *simulans*: the *N/S* ratio ranges from 7.11 (4.97, 10.95) to 8.73(5.90, 14.29) across libraries, and all ratios are significantly higher than the neutrally expected ratio (*P* < 0.005 in each comparison, Fisher’s exact tests; Table 2). We also observed higher *N/S* ratios for the conserved editing sites in X chromosome compared to those in the autosomes (S15B Fig). Importantly, the adaptation signals are primarily detected in the PSEB genes, and considerably lower *N/S* ratios (ranging from 2.73 to 3.60) were observed in the non-PSEB genes (S11 Table), suggesting the conserved *N* sites in the non-PSEB genes are unlikely favored by natural selection. Analogous results were obtained for the conserved editing sites between *D*. *melanogaster* and *D*. *pseudoobscura* (S12 Table). Altogether, our results suggest that the *N* editing sites in the PSEB genes are favored and maintained by natural selection while the *S* editing sites, which are putatively neutral, might degenerate during long-term evolution, which generates even higher *N/S* ratios in the sites with editing events conserved between species [50, 68].

**Fig 5:**
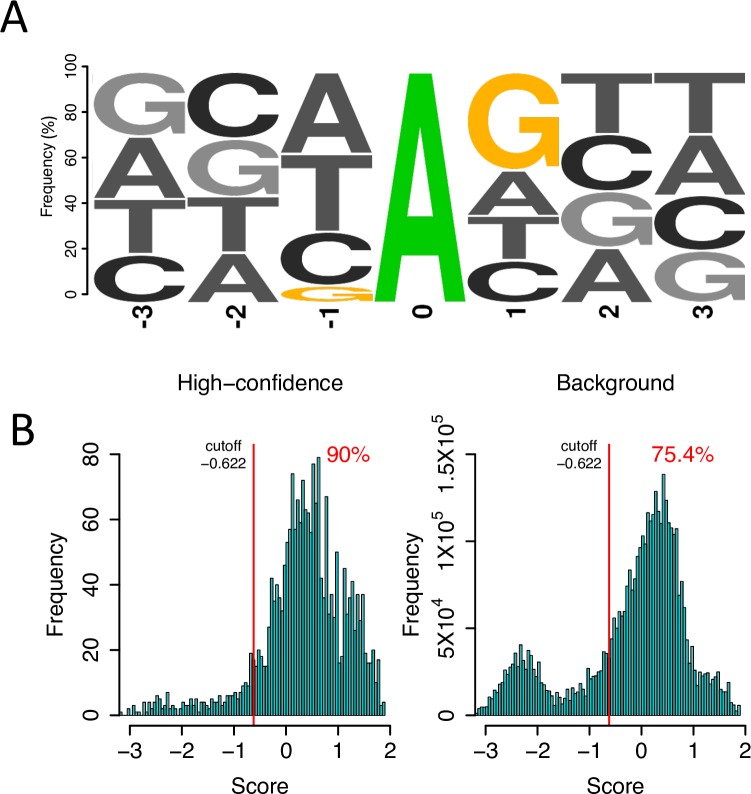
The effect of local nucleotide contexts on editing in brains of *D*. *melanogaster*. (A) A 7-mer motif centered with the high-confidence editing sites. (B) The score cutoff that specified the top 90% quantile of the high-confidence editing sites. (-0.622) corresponds to the top 75.4% of all the 7-mer sequences centered with adenosine in the genes with editing events.

Next we evaluated the effect of local nucleotide contexts on the comparison of observed vs. expected *N/S* ratios. For each of the 2,114 high-confidence editing sites, we extracted the upstream and downstream 3 nucleotides (we also used other number of nucleotides and obtained similar results), counted the number of nucleotide at each position (S17 Table), and developed a position probability matrix (S18 Table). Consistent with observations in primates that local nucleotide contexts affect the editing efficiencies [60, 88], we also found that G immediately upstream a focal editing site was generally not favored, the nucleotide immediately downstream the editing site was slightly biased toward G, and other flanking nucleotides were generally not important, although the overall patterns of preferences were weak in *D*. *melanogaster* (Fig 5A; we presented the frequencies of the tri-nucleotides centered with the editing sites and background adenosines in S19 Table). We scanned mRNAs of the edited genes with the position probability matrix and scored each 7-mer sequence that was centered with adenosine (Materials and Methods). We chose the score cutoff that specified the top 90% quantile of the high-confidence editing sites (-0.622), and found 75.4% of the background 7-mer sequences were above this score cutoff (Fig 5B). For the background adenosine sites with scores above the cutoff, the expected *N/S* ratio was 3.11 (even lower than 3.80, which was the expected *N/S* ratio based on all the adenosine sites), significantly lower than the observed *N/S* ratio (4.62) for all the high-confidence editing sites that have scores above the threshold (*P* < 0.0001, Fisher’s exact test). Therefore, our conclusions based on the comparisons between the observed and expected *N/S* ratios are not affected after considering effect of the local nucleotides contexts on editing.

### Editing in brains preferentially increases mutation sequence space of evolutionarily conserved genes

**Fig 6:**
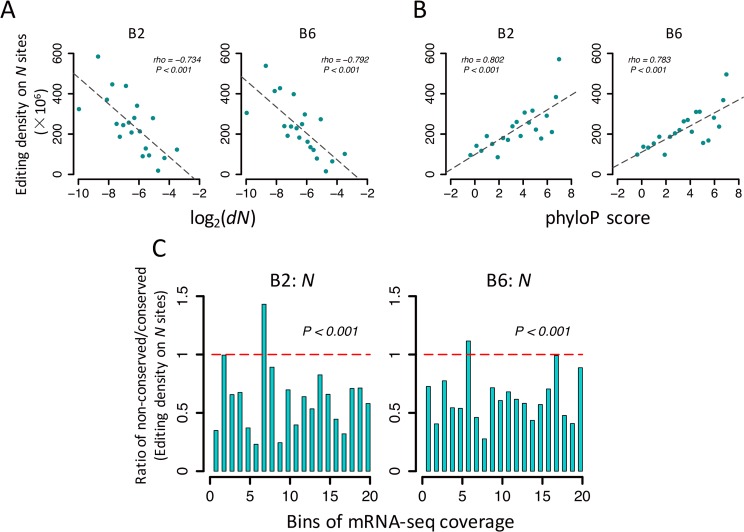
A-to-I editing increases mutation sequence space of evolutionarily conserved genes. (A) The editing density in the *N* sites is significantly inversely correlated with the *dN* value (between *D*. *melanogaster* and *D*. *simulans*) of the host genes. The genes expressed in brains are ranked with increasing *dN* values and divided into 20 bins (the *x*-axis, and lower *dN* means evolutionarily more conserved). The left and right panel is for 1- to 5-day female (B2) and male (B6) brains of *D*. *melanogaster*, respectively (Table 1). In each bin, the editing density (*y*-axis) is calculated by dividing the observed number of editing sites with the total number of adenosine sites that cause amino acid changes if edited. (B) The editing density in the *N* sites is significantly positively correlated with the phyloP score of the sites. All the nonsynonymous adenosine sites (cause amino acid changes if edited; ≥ 5X sequencing coverage) are ranked with increasing phyloP scores and grouped into 20 bins (*x*-axis, and higher phyloP score means evolutionarily more conserved). (C) The editing density of the *N* sites is significantly lower in the non-conserved compared to conserved sites after controlling mRNA-Seq coverage. All the nonsynonymous adenosine sites (cause amino acid changes if edited; ≥ 5X) are ranked with increasing sequencing coverage and binned into 20 categories (*x*-axis). Within each bin, we further divided the sites into two equal-sized subgroups based on the phyloP scores. The *y*-axis is the editing density of the non-conserved relative to the conserved subgroup in each bin.

We calculated the divergence of all the protein-coding genes between *D*. *melanogaster* and *D*. *simulans* (Materials and Methods), and we found PSEB genes have significantly lower *dN* and *dS* values compared to the non-PSEB genes expressed in brains of *D*. *melanogaster* (*dN* is 0.0107 ± 0.0012 vs. 0.0193 ± 0.0003 for PSEB vs. non-PSEB, and *dS* is 0.1025 ± 0.0025 vs. 0.1262 ± 0.0005 for PSEB vs. non-PSEB; *P* < 10^−8^ in both comparison, KS test), possibly due to the anti-correlation between expression levels and evolutionary rate [88–91] since the PSEB genes are usually expressed at higher levels (S16 Fig). These observations are consistent with the hypothesis that A-to-I editing increases the mutation sequence space of protein-coding genes. Next we ask whether we can detect higher editing densities in the evolutionarily conserved genes. We ranked all the protein-coding genes expressed in *Drosophila* brains with increasing *dN* values between *D*. *melanogaster* and *D*. *simulans* and grouped the genes into 20 bins. The editing density of the *N* sites (≥5X coverage) was significantly inversely correlated with the *dN* value in each bin (*rho* ranges from -0.824 to -0.734, *P* < 0.001 in each library, S20 Table; see Fig 6A for 1- to 5-day female and male brains). We observed similar patterns when we ranked all the possible nonsynonymous adenosine sites (≥ 5X coverage was required) with increasing phyloP scores (higher scores mean higher conservation levels) and grouped them into 20 bins: in each library, the density of *N* sites is significantly positively correlated with the median phyloP score of that bin (*rho* ranges from 0.711 to 0.832; *P* < 0.001 in each library, S21 Table; Fig 6B for 1- to 5-day female and male brains). Analogous but weaker correlations were observed for the *S* sites when we grouped the genes with *dN* value (*rho* ranges from -0.560 to -0.402 for each individual library, *P* < 0.1 in each library, S20 Table) or grouped the synonymous editing sites with phyloP scores (*rho* ranges from 0.519 to 0.738; *P* < 0.05 in each library, S21 Table). However, one potential pitfall of our analysis is that the conserved genes (with low *dN*) or sites (with high phyloP scores) usually have higher expression levels [88–91], which would lead to detection bias as higher editing densities were found in genes (or sites) with higher sequencing coverage (S4A and S4B Fig). To exclude such a possibility, in each library, we ranked all the nonsynonymous adenosine sites with increasing sequencing coverage (≥ 5X was required) and grouped them into 20 bins. Within each bin, we further divided the sites into two equal-sized subgroups based on the phyloP scores. In each library, the editing density in the nonsynonymous adenosine sites is significantly higher in the conserved subgroup compared to the non-conserved subgroup (*P* < 0.001 in each comparison, paired *t* test, Fig 6C and S18 Fig). Analogous patterns were observed for the synonymous editing sites as well (S19 Fig). Therefore, the elevated occurrences of A-to-I editing events in the evolutionarily conserved genes (or sites) in *Drosophila* are not likely caused by detection bias due to gene expression levels (or bias of sequencing coverage). Altogether, our results further support the adaptation hypothesis of RNA editing [12–14] since the sequence space of the evolutionarily conserved genes is generally inaccessible through DNA mutations, while RNA editing provides an epigenetic approach to expand proteomic diversity temporally and spatially.

### Beneficial nonsynonymous editing in the populations of *D*. *melanogaster*

So far our analysis revealed prevalent beneficial editing sites in brains of *Drosophila* and the majority of them were enriched in the PSEB genes. Next we asked whether we can observe similar patterns in whole bodies of *D*. *melanogaster* adults. We deep sequenced the poly(A)-tailed transcriptomes of female and male adults from five strains of *D*. *melanogaster* that were collected from five continents [79] (Materials and Methods). We sequenced 15.4–28.6M reads that were mapped on the reference genome in each library (S1 Table), and the median sequencing coverage on an exonic site in a library ranges from 17 to 31 reads in female, and ranges from 11 to 21 in male adults (S20 Fig). We masked all the SNPs in these five and other related strains which were sequenced previously [79], so that we only focused on the DNA sites that were exclusively adenosines across the five strains of *D*. *melanogaster*. For each site with A-to-G difference in a strain *k*, we calculated the probability that this site was edited *P*_*k*_(*E*_1_), and then we calculated the joint probability that this site was edited in at least one strain *P*(*E*_1_). We analyzed female and male adults independently and required each site to have at least 10 raw reads in each library. At FDR of 0.05, we identified 910 candidate editing sites in female and 1,458 candidates in male adults in exons. We obtained a false positive rate of 4.17% (26 out of 624) in female and 2.99% (29 out of 969) in male adults with the *w*^*1118*^ vs. *Adar*^*5G1*^ mutant analysis. After removing the false positive sites in *Adar*^*5G1*^, we obtained 875 exonic editing sites in female (S22 Table) and 1,422 exonic sites in male adults (S23 Table) (719 overlapped between female and male adults).

We obtained mixed results in detecting signals of adaptation when we compared the observed *N/S* ratios to the expected under neutrality (3.80) in both female and male adults (compared to the neutral expectation, the observed *N/S* ratio was significantly higher in strain B12 and N10, significantly lower in T07 and ZW155, and no significant difference was observed in I17, Table 4). As observed in brains, the *N/S* ratio was significantly higher in PSEB genes compared to neutral expectation in all the five strains (*P* < 0.001 in each comparison; Table 4). In contrast, in all the five strains the observed *N/S* ratio was significantly lower in the non-PSEB genes compared to neutral expectation (*P* < 0.001 in each comparison; Table 4). The patterns held when we separated the genes with editing events into “Highly” and “Lowly” expressed groups based on their expression levels (S24 Table). We also conducted simulations by randomly sampling the reads covering each site with increasing cutoffs of coverage (*C*_*min*_) or editing level (*l*_*min*_), and our simulation results constantly confirmed these observed patterns (S21 Fig). Our results indicate the *N/S* ratio is much higher in editing sites in the PSEB genes while strong purifying selection is acting on editing sites in the non-PSEB genes.

**Table 4:** The editing sites detected in female and male adults in five strains of *D*. *melanogaster*.

Strain	Gender	Total sites	PSEB				non-PSEB				Total			
			*N*	*S*	*N/S*	*P* value	*N*	*S*	*N/S*	*P* value	*N*	*S*	*N/S*	*P* value
B12	F	628	218	17	12.8	1.8×10^−8^	127	62	2.05	1.5×10^−4^	345	79	4.37	0.28
I17	F	530	165	7	23.6	6.4×10^−10^	101	65	1.55	7.8×10^−8^	266	72	3.69	0.84
N10	F	464	138	4	34.5	6.0×10^−10^	107	55	1.95	9.5×10^−5^	245	59	4.15	0.62
T07	F	524	146	4	36.5	1.2×10^−10^	133	65	2.05	7.4×10^−5^	279	69	4.04	0.69
ZW155	F	548	149	10	14.9	6.6×10^−7^	155	87	1.78	7.3×10^−8^	304	97	3.13	0.10
All female sites		875	235	20	11.8	2.8×10^−8^	251	131	1.92	9.8×10^−10^	486	151	3.22	0.08
B12	M	1102	349	39	8.95	2.7×10^−8^	305	132	2.31	3.8×10^−6^	654	171	3.82	1.00
I17	M	831	254	18	14.1	1.7×10^−3^	191	112	1.71	1.0×10^−10^	445	130	3.42	0.28
N10	M	824	252	17	14.8	7.6×10^−11^	195	114	1.71	9.2×10^−11^	447	131	3.41	0.28
T07	M	947	293	26	11.3	1.2×10^−9^	218	143	1.52	6.2×10^−16^	511	169	3.02	0.01
ZW155	M	1057	304	30	10.1	1.0×10^−8^	297	171	1.74	5.9×10^−15^	601	201	2.99	0.004
All male sites		1422	387	49	7.9	1.7×10^−7^	441	235	1.88	4.92×10^−17^	828	284	2.92	1.6×10^−4^

In total 875 exonic editing sites in female and 1,422 exonic sites in male adults were identified. In each library (*k*), we report the number of sites that meet the following criteria: 1) editing level > 0.01; 2) mRNA-seq coverage ≥ 10, 3) edited G alleles ≥ 2, and 4) *P*_*k*_*(E*_*0*_*)* < 0.05.

We uncovered significant but not high correlations in editing levels between strains in female (pairwise Pearson’s *r* was 0.718 ±0.018, S25 Table) and male adults (*r* was 0.804 ±0.013, S26 Table), suggesting that editing levels were variable across strains of *D*. *melanogaster* as previously observed [41]. Notably, we found a considerable number of sites at which editing events was readily detected in certain strains while absent in the other strains. We hypothesize that editing in such sites might be polymorphic in the populations, although reliably identifying such sites is challenging. We took a multiple-step procedure to identify the polymorphic editing sites from all the sites we detected (875 in female and 1,422 in male adults). First, we filtered the sites that have editing events detected in *D*. *simulans* or *D*. *pseudoobscura*. Second, we required a site to have editing reliably detected in at least one strain *k* with *P*_*k*_(*E*_1_) > 0.999 and to have no editing detected in at least one strain *m*. Third, we calculated *P*_*m*_(*D*_0_), the probability that the editing was not detected at depth *C*_*m*_ due to sampling bias or sequencing error in the strain *m*. Finally we calculated the joint probability *P*(*D*_0_) if no editing was observed in multiple strains at that site (Materials and Methods). For each site, a required parameter in calculating *P*_*m*_(*D*_0_) in the strain *m* which has no editing detected is the authentic editing level in that strain. We used two levels of stringency to calculate *P*_*m*_(*D*_0_). Level I: at each site, we assumed editing level is identical across all the strains, and the averaged level from the strains with reliably editing signals was used. Level II: we assumed the editing level is 0.05 in the strain with no editing detected if the averaged level from the detected strains is greater than 0.05, and the averaged level was used if it was smaller than 0.05. We conducted the analysis in females and males independently. Finally under Level I we obtained 165 and 179 candidate polymorphic editing sites in females and males respectively (57 sites overlapped). The averaged editing level for a site (based on the strains with reliable editing events) is 0.125±0.010 in females and 0.095±0.008 in males (we obtained 117 and 125 candidate sites under Level II in females and males respectively, and 44 sites were overlapping; S27 and S28 Table). Interestingly, among these putatively polymorphic editing sites, we did not find signal of adaptation in editing of the PSEB genes (in females the *N/S* ratio was 4.67 and 3.5 under Level I and II respectively; and in males the *N/S* ratio was 3 and 2.25 under Level I and II respectively; Table 5). Moreover, we observed even lower *N/S* ratios in editing of the non-PSEB genes (1.47 and 1.48 under Level I and II respectively in females; 1.96 and 1.74 under Level I and II respectively in males; Table 5). We observed similar patterns when we individually examined the *N* and *S* sites with respect to the number of strains in which such editing events were detected (S29 Table).

**Table 5:** The *N/S* ratios for the polymorphic and fixed editing sites detected in female and male adults in five strains of *D*. *melanogaster*.

	Female					Male				
	Polymorphic		Fixed	*P* value (fixed vs. polymorphic)		Polymorphic		Fixed	*P* value (fixed vs. polymorphic)	
	Level I	Level II		Level I	Level II	Level I	Level II		Level I	Level II
All sites										
*N*	89	66	109			114	77	248		
*S*	54	42	13			55	43	40		
*N/S* ratio	1.65	1.57	8.38	1.7×10^−6^	4.2×10^−6^	2.07	1.79	6.20	2.6×10^−5^	8.2×10^−6^
*P* value	3.9×10^−6^	2.3×10^−5^	0.015			4.0×10^−4^	1.7×10^−4^	0.02		
Sites in PSEB genes										
*N*	14	7	88				18	9	171		
*S*	3	2	2			6	4	5		
*N/S* ratio	4.67	3.50	44.0	0.034	0.047	3.00	2.25	34.2	7.7×10^−4^	0.002
*P* value	1	1	1.6×10^−6^			0.616	0.326	8.3×10^−11^		
Sites in non-PSEB genes											
*N*	75	59	21			96	68	77		
*S*	51	40	11			49	39	35		
*N/S* ratio	1.47	1.48	1.91	0.84	0.83	1.96	1.74	2.20	1	0.67
*P* value	4.8×10^−7^	9. ×10^−6^	0.042			3.0×10^−4^	1.9×10^−4^	2.5×10^−3^		

The *N/S* ratio expected under neutral evolution in *D*. *melanogaster* (3.80) was used to compare the observed *N/S* ratio in the polymorphic sites; and the *N/S* ratio expected under neutral evolution for *D*. *melanogaster* / *D*. *simulans* conserved adenosines (4.18) was used to compare the observed *N/S* ratio in the fixed sites. In the comparisons between fixed and polymorphic editing sites, the background difference was adjusted.

In contrast, we observed significantly higher *N/S* ratios in the editing sites of PSEB genes that were fixed in *D*. *melanogaster*. To reliably detect the sites with editing events fixed in the populations of *D*. *melanogaster*, we first identified the sites at which the probability of editing in each strain *P*_*k*_*(E*_*1*_*)* > 0.95 (*k* was B12, I17, N10, T07 and ZW155; female and male adults were studied separately). Due to the small number of strains we used, we further sequenced the transcriptomes of female and male adults of *D*. *simulans* and required the orthologous sites to be edited in the same gender of *D*. *simulans*. We obtained 181 sites (101 in PSEB and 80 in non-PSEB genes, S30 Table) fixed in female and 373 editing sites (194 in PSEB and 179 in non-PSEB genes, S31 Table) fixed in male adults (171 overlapped between female and males). The editing levels are higher in the fixed sites compared to the polymorphic ones: (the averaged editing level *per* site is 0.149±0.014 in female and 0.197±0.011 in male adults). In Table 5 we show the fixed editing sites have significantly higher *N/S* ratios in PSEB genes (44 in females and 34.2 in males) compared to the neutral backgrounds (4.18, calculated with the adenosine sites that are in the genes expressed in adults and conserved between *D*. *melanogaster* and *D*. *simulans*). Furthermore, in the PSEB genes the *N/S* ratio is significantly higher in the fixed editing sites compared to the sites showing polymorphic editing patterns in females and males after adjusting the background difference (Table 5). Nevertheless, strong signals of purifying selection were observed in the fixed editing sites in the non-PSEB genes (Table 5). We also did not find significant difference in the *N/S* ratio between the fixed and polymorphic editing sites in the non-PSEB genes in both female and male adults (Table 5).

Previous studies have demonstrated that mutations influence mRNA secondary structures and hence the efficiency of RNA editing in natural populations of *D*. *melanogaster* [41, 92]. Here we ask whether we can find SNPs associated with the variations in editing levels across the five strains of *D*. *melanogaster*. To increase the statistical power, we only focused on the 58 editing sites that are polymorphic (under Level I) in both female and males (Materials and Methods). Finally, we found 39 out of the 58 sites are associated with SNPs that are within 10kb flanking the editing sites, and on average each editing site is associated with 22.4 ± 3.7 SNPs, and the nearest distance between a SNP and the editing site is 770 ± 140 nts (S32 Table). Meanwhile, we conducted the same analysis on the 171 sites that have editing events fixed in both females and males, however, we did not observe any of those sites have editing levels associated with SNPs under the same criteria. These comparisons suggest cis-regulatory elements affect the levels or status of editing across strains, as previously observed [41, 84, 92].

### The effect of temperature on A-to-I editing levels in brains of *Drosophila*

**Fig 7:**
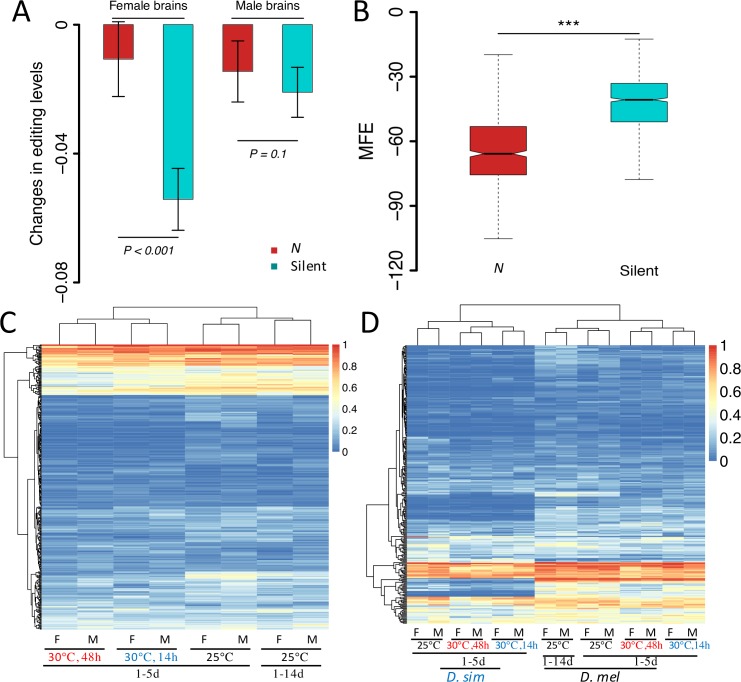
The effect of temperature on editing levels in brains of *Drosophila*. (A) The changes of editing levels in *N* and silent (*S* and UTRs) sites in female and male brains under elevated temperature (stressed at 30°C for 48 hours). (Error bar represents the s.e. of the level changes for editing sites in each category). (B) The flanking sequences (100 nts at each side) have significantly lower MFE (Kcal/mol) for the *N* sites compared to the silent sites. (C) Clustering the brain libraries of *D*. *melanogaster* based on the editing levels of 391 high-confidence editing sites that have at least 20 raw reads in each brain library. Note flies of the same accommodation conditions always cluster together. (D) Clustering the brain libraries of *D*. *melanogaster* and *D*. *simulans* based on the editing levels of 289 high-confidence editing sites that have at least 20 raw reads in each brain library. Note species divergence plays a more important role than temperature in clustering the samples.

Compared to hard-wiring a particular codon change in the genome, A-to-I editing may give the flexibility to quickly respond to environmental stress and adjust the activity of final protein product accordingly [15]. For example, the A-to-I editing level can be regulated by external stimuli [93, 94], energy and nutrient [95], and hypoxic conditions [96, 97]. Notably, temperature increases would reduce the thermo-stabilities of mRNA secondary structures and down-regulate the expression level of *Adar* in *Drosophila*, both of which would reduce the global editing efficiency [16]. The editing levels of *Adar* and a handful of other genes (totally 54 sites) have been examined under elevated temperatures [16]. Nevertheless, how temperature affects the editing sites at the genome-wide level remains unclear. Herein, we compared the editing levels in the brains of 1- to 5-day-old fly adults constantly raised at 25°C with those in the brains of flies raised at 25°C but treated at 30°C for 48 hours (*i*.*e*., B2 vs. B4 for female, and B6 vs. B8 for male brains of *D*. *melanogaster* in Table 1). The changes of editing levels were significantly positively correlated between *D*. *melanogaster* and *D*. *simulans* (S22 Fig; see Table 2 for information of *D*. *simulans*), consistent with previous observation that editing level changes under higher temperature were evolutionarily conserved[16]. The editing levels in general decreased under elevated temperatures for all functional categories of editing events, however, the decrease in editing levels was considerably smaller for the nonsynonymous compared to the silent sites (Fig 7A). Interestingly, when we folded the flanking sequence of each editing event (100 nts at each side), we uncovered the nonsynonymous editing sites were located in more stable secondary structures than all the silent events (*P* < 10^−16^, Kolmogorov-Smirnov test, Fig 7B). This comparison suggests that *Drosophila* has evolved mechanisms to maintain or even enhance the levels of some nonsynonymous editing sites under elevated temperature, which might be important for temperature adaptation since they expanded proteomic diversities.

We observed high correlations in editing levels between pairwise brain libraries (Pearson’s *r* ranges from 0. 849 to 0.933, S33 Table), and interestingly, the highest correlation coefficients were usually observed between brains of flies that were maintained at the same accommodation conditions but not of the same gender. The pattern was more pronounced when we clustered the samples based on the editing levels of the high-confidence sites (totally 391 sites that had at least 20 raw reads in each brain library): Females and males of 1–14 day old (B1 and B5, see Table 1 for annotations) clustered together; females and males of 1–5 day old that were constantly raised at room temperature (B2 and B6), treated at 30°C for 14 hours (B3 and B7), and treated at 30°C for 48 hours (B4 and B8) always clustered together (Fig 7C; similar patterns were observed when the coverage cutoff was set 15 or 25 raw reads, S23A Fig). Moreover, we did not find any site with editing level significantly different between female and male brains of *D*. *melanogaster* under the same accommodation conditions (*i*.*e*., B1 vs. B5, B2 vs. B6, B3 vs. B7 or B4 vs. B8) after multiple testing corrections. Therefore, our results indicate that temperature plays a more important role than gender effect in shaping the global brain editomes. However, we found the species effect is generally stronger than the temperature effect on the editing levels when we clustered the samples of *D*. *melanogaster* and *D*. *simulans* with 289 sites that have at least 20 raw reads in each library (Fig 7D) or when we clustered the samples of *D*. *melanogaster* and *D*. *pseudoobscura* with 152 sites that have at least 20 raw reads in each library (S23B Fig): the samples of the same species always clustered first, then the temperature conditions, and the gender effect was still very weak.

Gene expression plasticity is a strategy organisms evolved for adapting to new environments [97]. Yet it remains elusive whether (and how) RNA editing and gene expression plasticity coordinately participate in temperature stress responses. We detected hundreds of genes that were significantly differentially expressed in the brains of *D*. *melanogaster* that were constantly raised at 25°C compared to those raised at 25°C and treated at 30°C for 48 hours (B2 vs. B4 for female, and B6 vs. B8 for male brains). The down-regulated genes under elevated temperatures were enriched in the “oxidative phosphorylation” pathway in both female and male brains, while the up-regulated genes were enriched in the “ATP binding,” “translation” and “response to temperature” functional categories in female brains and in the “ATP binding” and “regulation of transcription” pathways in male brains (S34 and S35 Tables), which suggested gene expression plasticity was involved in the temperature stress responses but in a sexually dimorphic manner [98, 99]. Interestingly, in both female and male brains of *D*. *melanogaster*, the changes in editing levels of the nonsynonymous sites were weakly but significantly positively correlated with the changes in expression levels of the host genes under various cutoffs of expression levels (S24 Fig), suggesting these correlations were not artifacts caused by gene expression cutoffs. For example, a nonsynonymous A-to-I editing site (chrX:1781840) in *Adar* mRNA causes a Ser (AGU) to Gly (IGU) change (abbreviated as S>G change), and a previous study [16] and our data both indicated that the editing level of this S>G change (S5A Fig) and expression level (S25 Fig) of *Adar* mRNA were reduced in both female and male brains of *D*. *melanogaster* under elevated temperatures. In contrast, for synonymous sites we did not observe significant correlations between changes in editing levels and gene expression levels (S24 Fig). Altogether, these results suggest that the nonsynonymous editing events might interplay with gene expression changes in temperature adaptation, although detailed mechanisms remain to be further explored.

## Discussion

**Fig 8:**
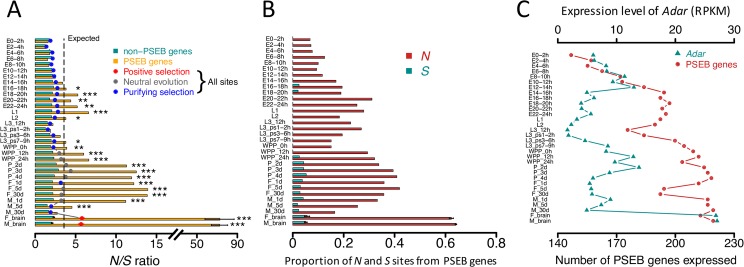
The expression profiles of PSEB genes and the expression pattern of *Adar* determine the overall *N/S* ratio of the editome in different developmental stages or tissues. (A) The *N/S* ratios (*x*-axis) for the all the editing sites (dots), the editing sites in PSEB genes (orange) or non-PSEB genes (cyan) in different developmental stages of *D*. *melanogaster* in the modENCODE Project. Asterisks indicate different *N/S* ratios between PSEB and non-PSEB editing sites: *, *P* < 0.05; **, *P* < 0.01; ***, *P* < 0.001. The overall observed *N/S* ratios were compared to the expected *N/S* ratio under neutral evolution (3.80): red dots, positive selection; grey dots: neutral evolution; blue dots, purifying selection. (B) The proportions of *N* and *S* sites that are contributed by PSEB genes in different development stages. (C) The expression level of *Adar* (RPKM) and the number of PSEB genes expressed during the development of *D*. *melanogaster*.

By extensively characterizing RNA editing sites in brains of *D*. *melanogaster* and two related *Drosophila* species, we identified a considerable number of *N* sites in *Drosophila* brains that were adaptive and maintained by natural selection during evolution. Our analysis revealed the *N/S* ratios in the editomes of *Drosophila* brains were significantly higher than the neutral expectation. In contrast, we did not observe such a pattern in the editing sites of different developmental stages or whole flies of *D*. *melanogaster* that were identified by the modENCODE Project [47] (Fig 8A) or re-analyzed by Ramaswami *et al*. [52] (S26 Fig). We also obtained mixed results when we compared the overall *N/S* ratios to the expected ratio under neutral evolution in female and male adults from five strains of *D*. *melanogaster* (Table 4). We found the significantly higher *N/S* ratios in the whole editomes of brains are mainly contributed by the *N* sites in the PSEB genes, which are favored by natural selection. The *N/S* ratio for editing sites in the PSEB genes is significantly higher than the neutral expectation in most developmental stages except in early embryos (0–16 hours) or larvae (Fig 8A), while *N/S* ratio for editing sites in the non-PSEB genes is lower than neutrality in all those samples (Fig 8A). Importantly, in brains, ~60% of the *N* sites were contributed by PSEB genes, while only ~5% of the *S* sites were from PSEB genes, which considerably elevated the overall *N/S* ratio in the brain editomes (Fig 8A). In contrast, in the late embryo, pupae and adults, although the *N/S* ratios for the PSEB sites were significantly higher than neutral expectation, less than 40% of the *N* sites were contributed by PSEB genes in each stage/tissue (Fig 8B), and hence, the signatures of adaptation in PSEB sites are masked by the non-PSEB sites when pooling all the editing sites together (Fig 8A). These patterns were constantly observed when we independently considered the “Highly” and “Lowly” expressed genes that have editing events in these samples (S27 Fig). We also observed a trend that the editing levels in the PSEB sites were increased during *Drosophila* development (S28 Fig). Furthermore, we observed a significant positive correlation between the expression level of *Adar* and the number of editing sites during *Drosophila* development in the modENCODE Project [47](Spearman’s *rho* = 0.413, *P* = 0.023; S29A Fig). And the expression level of *Adar* was also positively correlated with the cumulative editing levels (*i*.*e*., summing up the editing levels of all the sites, as following Ref. [63]) in the modENCODE samples (*rho* = 0.487, *P* = 0.006; S29B Fig). Importantly, the expression level of *Adar* is higher in brains compared to all the modENCODE developmental stages (Fig 8C), and accordingly, we observed the highest number of editing sites in brains compared to other developmental stages (S30 Fig). Notably, in early embryos (0–16 hours) and larvae, either *Adar* was lowly expressed, or only a few PSEB genes were expressed in these stages (Fig 8C), which putatively explains why we did not detect significantly higher *N/S* ratio for editing sites in PSEB genes in these stages (Fig 8A). Taken together, our results demonstrate that the expression level of *Adar*, together with the expression profiles of the PSEB genes that have editing sites favored by natural selection, are important in shaping the overall *N/S* ratios in the global editomes at different developmental stages (or tissues) of *D*. *melanogaster*.

We were able to predict secondary structures for ~74% of the exonic editing sites, including ~22% as long-range pseudoknots, which were often very challenging to find. Our results help understanding the molecular basis by which the editomes are regulated and maintained. First, our analysis reveals a considerable number of editing sites could be clustered due to promiscuous editing of multiple adenosines by ADAR simultaneously. Second, we found a significantly higher proportion of the *N* sites were located in stable hairpin structures of mRNAs than the silent sites (55.0% vs. 41.7%, *P* < 0.001, Fisher’s exact test), and similarly, the flanking sequences of the *N* sites have significantly lower MFE compared to those of the silent sites (Fig 7B). Since ADAR recognize double-stranded RNAs to exert editing, these findings provide the structural basis for the observed higher editing levels and excessive occurrences of the *N* sites.

The density of editing events was significantly higher in the evolutionarily conserved genes than in the non-conserved ones (Fig 6C, S18 Fig), which supports the hypothesis that editing increases the mutation sequence space. However, another competing explanation for this observation is that the secondary structures (stable hairpins or pseudo-knots) of mRNAs, which are ADAR substrates for RNA editing, constrain the evolutionary rates of these genes. Indeed, when we separately calculated the evolutionary rates between *D*. *melanogaster* and *D*. *simulans* for the CDS regions that formed secondary structures and harbored editing events in *Drosophila* brains (termed “structured”) and the remaining CDS regions after masking the secondary structures (“structure masked”), we uncovered both *dN* and *dS* were significantly lower in the “structured” than “non-structured” CDS regions (*P* < 10^−10^ for both *dN* and *dS* comparisons, S31 Fig; totally 172 genes were included in the comparisons). Nevertheless, the length of the “structured” CDS regions in general only account for 4.36 ± 0.24% of the full lengths of CDS regions, and we still observed significantly lower *dN* and *dS* values in the “structure masked” CDS regions compared to the genes without editing events (“Unedited”) in *Drosophila* brains (*P* < 10^−10^ for both *dN* and *dS* comparisons, S31 Fig). Hence, although the editing-associated secondary structures considerably constrain mRNA sequences, they generally have negligible impact on the evolutionary rates of the total CDS regions. In summary, our results support the hypothesis that A-to-I editing increases the proteomic diversity for the genes that are highly conserved due to functional constraints.

It is worth noting that compared to humans [67, 68] and macaques [59, 60], we observed strong signals of adaptation in the editing sites in *Drosophila*, especially in the brain editomes. Furthermore, the A-to-I editing events we identified in *Drosophila* were significantly enriched in evolutionarily conserved genes while the editing sites in human coding regions showed an opposite pattern [67]. The different observations between *Drosophila* and primates might be shaped by the difference in the underlying molecular mechanisms and selective forces. First, there are two catalytically active ADAR enzymes (ADAR1 and ADAR2) in primates and both enzymes are expressed in many tissues, which potentially cause promiscuous editing events that might be neutral or deleterious [59, 60]. Second, the targets of editing are mainly repetitive non-coding sequences in primates [13]. Although the coding editing events conserved between human and mouse are adaptive [68], the majority of the editing events in coding regions might be solely by-products of the over-activity of ADARs and hence selected against [67]. In contrast, there is only one *Adar* locus in *Drosophila* [37, 38], which is predominately expressed in the nervous system and preferentially edits pre-mRNAs of neural genes [39]. Third, the effective population size is much larger for *D*. *melanogaster* than primates, which makes natural selection more efficient in the former than in the latter [100]. Therefore, the adaptive editing events, once originated, will be more effectively spread and fixed in *Drosophila* than in primates. On the other hand, the detrimental effects incurred by RNA editing, will be more efficiently selected against in *D*. *melanogaster* than in primates.

Besides providing proteomic diversity, our results also suggest that mRNA editing interplayed with gene expression plasticity to fine-tune gene expression activity under temperature stress responses, which supports previous hypothesis that RNA editing might be a driving force for environmental adaptation [15, 16]. Interestingly, we also found several editing events in *D*. *melanogaster* that compensated for the G-to-A DNA mutation in the *D*. *melanogaster* lineage after splitting with its sibling species (S32 Fig), which suggests that RNA editing events are advantageous because they reverse the deleterious effects caused by G-to-A DNA mutations as previously proposed [67, 101]. Taken together, our evolutionary analyses, combined with our functional genomic studies, shed new light on the molecular mechanisms and functional consequences of RNA editing in *Drosophila*.

## Materials and methods

### Female and male brains of three *Drosophila* species

Flies were grown in 12 hour light: 12 hour dark cycles at 25°C. The ISO-1 strain of *D*. *melanogaster*, the sim4 strain of *D*. *simulans*, and one lab strain of *D*. *pseudoobscura* were gifts from Dr. Andrew G. Clark’s lab at Cornell University. In the temperature stress experiments, 1- to 5-day-old flies were transferred from 25°C incubators to 30°C incubators and treated for 14 hours or 48 hours, and the humidity and light conditions were maintained at the same levels. The 1- to 5-day-old and 1- to 14-day-old flies were separately sexed, and the brains were dissected in RNAlater solution (Ambion). We also separated the heads and bodies of the female or male adults of the sim4 strain of *D*. *simulans* with a fine sieve and extracted the total RNAs from heads and bodies of each gender.

### RNA extraction, library construction and RNA sequencing

Total RNA was extracted using TRIzol reagent (Invitrogen) according to the manufacturer's protocol. Poly(A)+ mRNA was isolated from 15 μg total RNA with oligodT25 DynaBeads (Thermo Fisher). Next, the mRNA was fragmented and size selected from 40 nts to 80 nts by 15% TBU gels. Following 3' dephosphorylation, 3' ligation with a 3' adaptor, 5' phosphorylation and 5' ligation with a 5' adaptor, and size-selected mRNA fragments were reverse transcribed with SuperScript III (Invitrogen). The sequence of the 5' adaptor was 5'GUUCAGAGUUCUACAGUCCGACGAUC3' and the 3' adaptor was 5'TGGAATTCTCGGGTGCCAAGG3'. All cDNA was amplified by 14 PCR cycles with Phusion High-Fidelity DNA polymerase (NEB) with the TruSeq index adapters, and the products within the correct size ranges were collected from 20% TBE gels for the quality tests (Fragment Analyzer, Agilent Technologies) and sequencing (Platform: Illumina HiSeq 2500; read length: 50 bp, single-end).

### Sequence processing and analysis

The 3' adaptor sequences were clipped by the Cutadapt program [102]. The remaining reads were aligned to the reference genome of *D*. *melanogaster* (r6.04), *D*. *simulans* (r1.4) or *D*. *pseudoobscura* (r3. 2) using STAR [103] (mapping statistics were summarized in S1 Table). The genome sequences and annotations of the three *Drosophila* species were downloaded from FlyBase (www.flybase.org).

### Identification of editing sites in brains of *D*. *melanogaster*

First, for each brain library, we employed the GATK RNA-Seq variant calling pipeline [78] to detect the A-to-I editing events, where the transcriptomic base is Adenosine and the sequencing read variant base is Guanine (Inosine). We were able to identify 1,531 editing sites at this preliminary stage. To retrieve the editing sites that were potentially excluded by the pretty strict GATK pipeline, we pooled all the editing sites in four previous studies [47–50] and the GATK candidates altogether, constructing a list of 5,925 candidate sites. Second, for each candidate site in a brain library *k*, we discarded the reads with mapping quality lower than 10 and the reads with mismatches other than A-to-G, and extracted the sequencing coverage (*C*_*k*_) and the number of edited allele that shows A-to-G difference (*L*_*k*_) with SAMtools [104] and calculate the probability that a site is edited in a library *k* given the observed sequencing data *P*_*k*_(*E*_1_) = 1 − *P*_*k*_(*E*_0_), where *P*_*k*_(*E*_0_) is the probability the A-to-G difference is solely caused by sequencing error with a rate of *ε*. We define $P_{k}(E_{0})=\sum_{i=L_{k}}^{C_{k}}(\begin{array}{c} C_{k}\\ i \end{array})∙\varepsilon^{i}∙{\left(1-\varepsilon \right)}^{C_{k}-i}$. The Illumina HiSeq platform generally has an error rate *ε*_0_ ranging from 0.2% to 0.6% [105–107] and we used *ε*_0_ = 0.5% in the analysis. As we focus on the sites with solely A-to-G but not A-to-C or A-to-T mismatches, the error rate of A-to-G would be scaled to $\varepsilon =\frac{\varepsilon_{0}/3}{1-(2/3)\varepsilon_{0}}\approx 0.00167$. The probability that a site is edited in none of the *n* libraries is thus $P(E_{0})=\prod_{k=1}^{n}P_{k}(E_{0})$, where *n* is the number of brain libraries, and accordingly, the probability that this site is edited in at least one of these libraries is *P*(*E*_1_) = 1 − *P*(*E*_0_). For each of the 5,261 sites that have sequencing coverage in our brain libraries of *D*. *melanogaster*, we calculated *P*(*E*_1_) and *P*(*E*_0_), and corrected for multiple testing with Benjamini & Hochberg method [108]. The procedures are summarized in Fig 1A. The functional annotations of the editing sites were conducted with the software SnpEff [109].

### Estimating the false positive rate of editing sites

The transcriptome data for heads of the *Adar*^*5G1*^ mutant and paired wild type *w*^*1118*^ of *D*. *melanogaster* were downloaded from NCBI SRA under accession numbers SRR629970 and SRR629969 [51]. We mapped the transcriptome data to the reference genome of *D*. *melanogaster* using STAR [103] and extracted the A and G alleles with SAMtools [104]. To estimate the false positive rates of the editing sites we detected, we first examined the number of the editing sites we identified in *D*. *melanogaster* that also had editing events detected in the heads of *w*^*1118*^ strain (*N1*), and then we counted the number of these shared sites that also showed A-to-G difference in the *Adar*^*5G1*^ mutant (*N2*). The false positive rate is estimated by *N2*/*N1* as conducted previously [50, 51].

### Pre-mRNA secondary structure prediction

We folded all the transcripts in genes expressed in brains of *D*. *melanogaster* with RNALfold [110] and identified the exonic editing sites that were located in the stable local hairpin structures (*z* score < -1.5, *ΔG* < -15 kcal/mol, and the stem length > 50 nts). To obtain the expected numbers of editing sites in the hairpin structures by randomness, we randomly sampled the same number of exonic editing sites under study with replacement for 1000 replicates and counted the median and 2.5% and 97.5% quantiles in the simulations. For each introinc editing site, we folded the flanking sequences for each site (100 nts at each side) with RNALfold and examined whether this intronic site was located in the stable hairpin structures with the same criteria. For each of the editing events detected in *D*. *melanogaster*, the flanking sequences (100 nts at each side) were folded In the long-range pseudoknot searches, the full-length pre-mRNA or flanking sequences (2,000 nts at each flank, totally 4,000 nts for long pre-mRNAs) were folded with RNAfold [110] and putative long-range pseudoknots were parsed if the pairing region in each stem of the pseudoknot was > 40 nts and the distance between the two pairing stems was > 100 nts. We also fold the flanking sequences for each editing site (100 nts at each side) and calculated the MFE (kcal/mol) with RNAfold [110].

### Evolutionary divergence analysis

The protein and CDS sequences of *D*. *melanogaster* (r6.04), *D*. *simulans* (r1.4) and *D*. *pseudoobscura* (r3.2) were downloaded from FlyBase. The reciprocal best orthologous genes were obtained based on pairwise BLASTP [111] between *D*. *melanogaster* and *D*. *simulans* (r1.4), and between *D*. *melanogaster* and *D*. *pseudoobscura*. The protein sequences of the orthologous genes were aligned with the clustalw [112] program, and the CDS alignments were produced with the tranalign [113] program based on the corresponding protein alignments. The yn00 program in the PAML [114] package was employed to calculate the *dN* and *dS* values for each gene between *D*. *melanogaster* and *D*. *simulans*. The phyloP score for each site of *D*. *melanogaster* were downloaded from UCSC Genome Browser (genome.ucsc.edu).

### Evolutionarily conserved editing events in *Drosophila* brains

For each editing site in *D*. *melanogaster*, we employed two complementary approaches to search for the orthologous sites in *D*. *simulans* and *D*. *pseudoobscura*. First, we used liftOver [83] to convert the genomic coordinates of the orthologous sites in coding and non-coding regions between *D*. *melanogaster* and *D*. *simulans*, or between *D*. *melanogaster* and *D*. *pseudoobscura* as previously conducted [51] (termed “g_align” approach). The pairwise genome alignments between *D*. *melanogaster* and *D*. *simulans*, and between *D*. *melanogaster* and *D*. *pseudoobscura* were downloaded from UCSC Genome Browser (genome.ucsc.edu) and used to identify the evolutionarily conserved adenosine sites. Second, we parsed the genomic co-ordinates with the pairwise CDS alignments between *D*. *melanogaster* and *D*. *simulans*, and between *D*. *melanogaster* and *D*. *pseudoobscura* (termed “c_align” approach). The g_align approach efficiently identified both the coding and non-coding orthologous sites, and the c_align approach is powerful in identifying orthologous sites in coding regions between distantly-related species. Among the 2,114 high-confidence editing sites in brains of *D*. *melanogaster*, we identified 1,499 orthologous sites in *D*. *simulans* that were also adenosines and had sequencing coverage in at least one brain library of *D*. *simulans* (1,443 by g_align, 577 by c_align, and 521 by both), and 892 sites in *D*. *pseudoobscura* that were also adenosines and had sequencing coverage in at least one brain library of *D*. *pseudoobscura* (707 by g_align, 527 by c_align, and 342 by both). To exclude SNPs in the RNA editing characterization, we also deep sequenced the genomic DNA of the same strains of *D*. *simulans* (the median coverage per site is 46) and *D*. *pseudoobscura* (the median coverage per site is 47) that were used for RNA-editing detection. We mapped the DNA reads on the reference genomes with BWA[115], excluded reads with mapping quality lower than 10, and called the SNPs with SAMtools (313,133 SNPs in *D*. *simulans* and 489,828 SNPs in *D*. *pseudoobscura*). After masking the SNPs, for each site in each species, we calculated *P*(*E*_1_), the joint probability that this site is edited in brains. At FDR of 0.05, we identified 996 sites edited in *D*. *simulans* (947 by g_align, 495 by c_align and 446 by both), and 451 sites edited in *D*. *pseudoobscura* (340 by g_align, 326 by c_align, and 215 by both). Our experimental designs (brain samples of the same gender and the same age under the same accommodation conditions for different species) and the combinations of g_align and c_align approaches enabled us to identify more evolutionarily conserved editing events compared to Yu *et al*. [50] which mainly focused on the conserved editing events in the coding regions.

### Estimating the probability that a site with no editing event detected is truly not edited

For an editing site with coverage *C*_*m*_ and editing level *l*_*m*_ in a library *m*, no edited (G) allele would be detected if the edited RNA molecules were not sampled or all the edited signals were abolished by sequencing errors. Therefore, we estimated the probability of observing zero edited reads at this site in a sample *m* as $P_{m}(D_{0})=\sum_{i=0}^{C_{m}}(\begin{array}{c} C_{m}\\ i \end{array})∙l_{m}^{i}∙{\left(1-l_{m}\right)}^{C_{m}-i}∙\varepsilon^{i}∙{\left(1-\varepsilon \right)}^{C_{m}-i}$, where *ε* is the scaled sequencing error rate (0.00167). Then the joint probability that this site is edited but not detected in *n* libraries would be $P(D_{0})=\prod_{m=1}^{n}P_{m}(D_{0})$.

### Expected *N/S* ratio under neutral evolution

The expected *N/S* ratio for the editing events under neutral evolution was calculated with a similar procedure described previously [67]. Briefly, for each adenosine site in the CDS regions of the genes that have at least one editing event in brains of *D*. *melanogaster*, we tested whether it cause an amino acid change (nonsynonymous, *N*) or not (synonymous, *S*) when edited. To obtain the expected *N/S* ratios for the editing events that are evolutionarily conserved, we conducted similar analysis only on the conserved adenosine sites between *D*. *melanogaster* and *D*. *simulans*, or between *D*. *melanogaster* and *D*. *pseudoobscura*, as previously conducted between human and mouse [68]. The *N/S* ratio expected under neutral evolution is 3.80 for the editing sites in *D*. *melanogaster*, 4.18 for the editing sites with events conserved between *D*. *melanogaster* and *D*. *simulans*, and 7.11 for the editing sites with events conserved between *D*. *melanogaster* and *D*. *pseudoobscura*. We obtained the 95% confidence intervals by random sampling the same number of observed editing sites with replacement and calculated *N/S* ratios from the simulated data for 1000 replicates.

### Assessing the effect of detection bias on *N/S* ratio estimation with simulations

The detailed procedures of the two methods in evaluating the effect of detection bias on *N/S* ratio are fully described in TEXT. The processes of these simulations (and other simulations and statistical tests in this study) were performed using R (www.r-project.org).

### Motif associated with the focal editing sites

For each of the 2,114 high-confidence editing sites, we extracted the upstream and downstream 3 nucleotides flanking this site, counted the number of nucleotide at each position of the 7-mer, and developed a position probability matrix. Sequence logo for this motif was generated with WebLogo (http://weblogo.berkeley.edu). In order to get a well-controlled set of genomic background sites, we scanned the mRNA regions for other adenosine sites in genes where the 2,114 editing events were detected. Then we scored both the 2,114 high-confidence editing sites and the background adenosine sites with the same position probability matrix. For a given sequence N_-3_N_-2_N_-1_AN_1_N_2_N_3_ (N_i_ ∈ (*A*,*T*,*C*,*G*)), the score for this sequence was calculated as $\sum_{i}{log}_{2}\frac{P_{i}(N_{i})}{0.25}$, where *P*_*i*_(*N*_*i*_) is the probability of observing base *N*_*i*_ at position *i* (-3,-2,-1,1,2,3) based on the position probability matrix. We chose the score cutoff that specified the bottom 10% quantile of the high-confidence editing sites (-0.622). With that score cutoff, we chose the sites with scores above this cutoff (-0.622) in the high-confidence editing sites and background adenosine sites, and calculated the *N/S* ratio in each dataset.

### The A-to-I editing events in female and male adults from five strains of *D*. *melanogaster*

The 1–14 day old female and male adults from five strains (B12, I17, N10, T07 and ZW155) of *D*. *melanogaster* were sexed, the poly-A tailed mRNAs were isolated from females and males independently with the procedures described above. The libraries were prepared with NEBNext® Ultra™ Directional RNA Library Prep Kit for Illumina and the sequencing was carried out at Illumina HiSeq 2500 (read length: 100 bp, paired-end). These strains, kindly provided by Dr. Andrew G. Clark at Cornell University, were originally collected from five continents [79]: Beijing, China (abbreviated B); Ithaca, NY USA (abbreviated I); Netherlands, Europe (abbreviated N); Tasmania, Australia (abbreviated T); and Zimbabwe, Africa (abbreviated Z). These flies were grown in 12 hour light:12 hour dark cycles at 25°C. The A-to-G editing sites in the exonic regions were called with the “joint probability” method as above described, and the SNPs in these five strains and other 79 related strains based on the whole-genome re-sequencing information [79] were masked in the down-stream analysis. Finally we identified 875 editing sites in female and 1,422 exonic sites in male adults.

To identify the editing sites with putative polymorphic events across these five strains, we first filtered the sites that have editing events detected in brains of *D*. *simulans* or *D*. *pseudoobscura*, or whole files of *D*. *simulans*. And then we required a site to have editing reliably detected in at least one strain *k* with *P*_*k*_*(E*_*1*_*)* > 0.999 and to have no editing detected in at least one strain. Next we calculated *P*_*m*_*(D*_*0*_*)*, the probability that the editing was not detected at depth *C*_*m*_ due to sampling bias or sequencing error in a strain *m* as above described. Finally, we calculated the joint probability *P(D*_*0*_*)* if no editing was observed in multiple strains at that site. Under Level I in calculating *P*_*m*_*(D*_*0*_*)*, we assumed the strain *m* that has no editing event detected have the same editing level at that site as in the other strains which had reliably editing event detected (the mean value was used). Under Level II, we used 0.05 as the editing level in calculating *P*_*m*_*(D*_*0*_*)* if the mean editing level from the other strain is >0.05. To detect the editing events that were fixed across these five strains, we employed two complementary approaches: First we identified the sites at which the probability of editing in each strain *P*_*k*_*(E*_*1*_*)* > 0.95 (*k* was B12, I17, N10, T07 and ZW155; we studied female and male adults separately); and then we sequenced the transcriptomes of female and male adults of *D*. *simulans* (the sim4 strain) and required the orthologous sites to be edited in the same gender of *D*. *simulans*.

To identify the SNPs that are associated with the variation of the editing levels in female or male adults across these strains, we only focused on the editing sites that are polymorphic in both female and males (totally 58 sites) or fixed in both females and males (171 sites). For each site in females, we retrieved the SNPs within 10kb regions that flanked the focal editing sites in the five strains and conducted the association test between the editing level in each strain and the genotypes of a SNP (we assumed the reference allele as 0 and the alternative allele as 1). We also performed the same analysis in male adults. We required the reference and alternative allele of a SNP to be associated with editing levels of the same site in the same direction in females and males and *P* < 0.05 in both tests.

### Sanger sequencing

Total RNA from the female or male brains was prepared independently using Sanger sequencing procedures. The total RNA was treated with RNase-free DNase I (Invitrogen) to remove genomic DNA. Reverse transcription was performed using random primers, and cDNA was amplified using target-specific primers (the primers sequences are presented in S36 Table). The final PCR products were sequenced with the Sanger method at the Ruibiotech Sequencing Company.

### Quantitative RT-PCR

qRT-PCR was performed with SYBR Green Master Mix (Thermo Fisher) in a 20 μL reaction volume and monitored on a StepOnePlus Real-Time PCR System (Thermo Fisher). *rp49* was used as the internal control. The primers for the real-time PCR assay are listed in S37 Table.

### Gene expression analysis

The raw NGS reads for each gene were counted with the htseq-count program [116], gene expression levels were normalized, and differentially expressed genes were detected with the edgeR package [117]. The RPKM for each gene or half-gene was calculated with CuffLinks [118]. All of the gene ontology analyses were performed by DAVID [119] and all the brain-expressed genes were used as background list.

### Data access

The sequence data in this study have been submitted to the NCBI Sequence Read Archive (SRA, http://www.ncbi.nlm.nih.gov/sra) under accession number SRP074828 and SRP068882. All other relevant data are within the paper and SI files.

## Supporting information

S1 Table Summary of mRNA-seq libraries in brains and whole adults of *Drosophila*. *D*. *mel*, *D*. *melanogaster; D*. *sim*, *D*. *simulans; D*.*pse*, *D*. *pseudoobscura*. F: female; M: male. All flies are raised at 25°C. 30°C, 14h: the flies was raised at 25°C, and treated at 30°C for 14 hours; 30°C, 48h: the flies were raised at 25°C and treated at 30°C for 48 hours.
(PDF)

S2 Table The A-to-I editing events identified in this study and the overlapping status with editing sites detected in previous studies. (XLSX)

S3 Table Number of editing sites of Class I and II detected in this study at different cutoffs. The FDR is calculated based on the multiple test correction of Joint *P(E*_*0*_*)* with Benjamini & Hochberg method. For each site, we counted the number of brain libraries in which editing was detected at that site. The other criteria in defining Class I and II sites are the same as described in TEXT.
(PDF)

S4 Table The number of editing sites overlapped between previous studies and this study. (PDF)

S5 Table A-to-I editing sites in PSEB and non-PSEB genes in female and male brains of *D. melanogaster*. The criteria in identifying editing site in each single library and the annotation are described in Table 1.
(PDF)

S6 Table Gene Ontology (GO) analysis on genes with editing events in male brains of *D. melanogaster* (552 genes). (PDF)

S7 Table Gene Ontology (GO) analysis on genes with editing events in female brains *D. melanogaster* (534 genes). (PDF)

S8 Table The top 50 genes that have the highest number of A-to-I editing sites in brains of *D. melanogaster*. (PDF)

S9 Table 996 orthologous sites with conserved editing events between *D. melanogaster* and six brain libraries of *D. simulans*. Orthologous sites with deep sequencing coverage (raw reads ≥ 200 in all the libraries) but without editing detected are also presented.
(XLSX)

S10 Table The 451 orthologous sites with conserved editing events between *D. melanogaster* and six brain libraries of *D. pseudoobscura*. Orthologous sites with deep sequencing coverage (raw reads ≥ 200 in all the libraries) but without editing detected are also presented.
(XLSX)

S11 Table Detailed information about the RNA editing sites with events observed in brain of *D. melanogaster* and the matched brain sample of *D. simulans*. The criteria in identifying editing site in each single library and the annotation are described in Table 2. Please note, for each site, we require the editing events to be present in the matched brain samples of *D*. *melanogaster* and *D*. *simulans*.
(PDF)

S12 Table Detailed information about the RNA editing sites with events observed in brain of *D. melanogaster* and the matched brain sample of *D. pseudoobscura*. The criteria in identifying editing site in each single library and the annotation are described in Table 3. Please note, for each site, we require the editing events to be present in the matched brain samples of *D*. *melanogaster* and *D*. *pseudoobscura*.
(PDF)

S13 Table Orthologous sites that have edited events detected in brains of *D. melanogaster* and *D. pseudoobscura* but not have adequate evidence of editing in brains of *D. simulans*. (PDF)

S14 Table A-to-I editing sites (adjusted by over-estimation) in female and male brains of *D. melanogaster*. In each library and category, we adjust the numbers of *N* sites by dividing them with the over-estimation ratios (the median simulated *N/S* ratio divided by the observed *N/S* ratio) obtained from simulation. The numbers of adjusted N sites (*N’*) are given.
(PDF)

S15 Table Numbers of high-confidence editing sites in the coding regions of 223 PSEB genes (see the appended EXCEL table). (XLS)

S16 Table Number of *N* and *S* editing sites in Highly and Lowly expressed PSEB and non-PSEB genes in brains of *D. melanogaster*. (PDF)

S17 Table Position Frequency Matrix for the 7-mer nucleotides centered with the high-confidence editing sites (totally 2,114 sites). (PDF)

S18 Table Position Probability Matrix for the 7-mer nucleotides centered with the high-confidence editing sites (totally 2,114 sites). (PDF)

S19 Table The frequencies of the tri-nucleotides centered with the high-confidence editing sites and background adenosines in the edited genes. The score cutoff is -0.6216; the number of background adenosines is 4045312. The percentage of the triplets in each category is in the parenthesis.
(PDF)

S20 Table The correlation coefficients between the *dN* values (between *D. melanogaster* and *D. simulans*) and the editing density in nonsynonymous (*N*) and synonymous (*S*) sites in each binned fraction. (PDF)

S21 Table The correlation coefficients between the phyloP score and the editing density in nonsynonymous (*N*) and synonymous (*S*) sites in each binned fraction. (PDF)

S22 Table The 875 exonic editing sites in female adults from five strains of *D. melanogaster*. (XLSX)

S23 Table The 1,422 exonic editing sites in male adults from five strains of *D. melanogaster*. (XLSX)

S24 Table Number of *N* and *S* editing sites in Highly and Lowly expressed PSEB and non-PSEB genes in female and male adults from five strains of *D. melanogaster*. (PDF)

S25 Table Correlation coefficients (Pearson’s *r*) in editing level between female adults of different strains. (PDF)

S26 Table Correlation coefficients (Pearson’s *r*) in editing level between male adults of different strains. (PDF)

S27 Table The candidate editing sites with events polymorphic in female adults (165 under Level I and 117 under Level II). (XLSX)

S28 Table The candidate editing sites with events polymorphic in male adults (179 under Level I and 125 under Level II). (XLSX)

S29 Table The number of *N* and *S* editing sites in the five strains of *D. melanogaster*. Fn is the number of strains of *D*. *melanogaster* that has *N* and *S* editing events detected in those strains [only editing events with *P(E*_*1*_*)* > 0.999 were counted].
(PDF)

S30 Table The editing sites with events detected in female adults of the five strains of *D. melanogaster* and female adults of *D. simulans*. (XLSX)

S31 Table The editing sites with events detected in male adults of the five strains of *D. melanogaster* and female adults of *D. simulans*. (XLSX)

S32 Table The SNPs (within 10kb flanking the focal editing sites) that are associated with the polymorphic editing events in both female and male adults in the five strains of *D. melanogaster*. (XLSX)

S33 Table Correlation coefficients (Pearson’s *r*) in editing level between brain libraries of *D. melanogaster*. Only the high-confidence sites with ≥ 10 raw reads in each library are used in the pairwise comparisons.
(PDF)

S34 Table Gene Ontology (GO) analysis on the up- and down-regulated genes in female brains of *D. melanogaster* under elevated temperature (30°C for 48 hours). (PDF)

S35 Table Gene Ontology (GO) analysis on the up- and down-regulated genes in male brains of *D. melanogaster* under elevated temperature (30°C for 48 hours). (PDF)

S36 Table Primer sequences for Sanger sequencing (5'-3'). (PDF)

S37 Table Primer sequences for qPCR of *Adar* (5'-3'). (PDF)

S1 Fig Distribution of mRNA-seq coverage of exonic region in brain libraries of *Drosophila*. (PDF)

S2 Fig Percentage of different types of DNA-RNA differences and SNPs in *D. melanogaster*. (**A**) Percentages of sites with different types of DNA-RNA differences detected in the female and male brains of *D*. *melanogaster* across eight libraries (the error bars represent the s.e. across eight libraries).
(**B**) Percentages of the sites of different types of SNPs (reference>alternative allele) from the global populations of *D*. *melanogaster*.
(PDF)

S3 Fig Sequencing coverage and editing levels of the editing sites in eight brains libraries of *D. melanogaster*. (A) Sequencing coverage (*y*-axis, at log2 scale) of five classes of editing sites (***, *P* < 0.001).
(B) Editing level of five classes of editing sites (***, *P* < 0.001).
(C) The percentage of Class I and Class II editing sites with respect to the number of brain libraries in which editing events are detected (*x*-axis). The number and percentage of editing sites are given above the bars.
(D) The sequencing coverage of common and novel editing sites in each brain library (*P* < 0.05 in each of the eight libraries, KS tests).
(E) The percentage of common sites with respect to the number of brain libraries in which editing events are detected (*x*-axis). The number and percentage of editing sites are given above the bars.
(F) The percentage of novel sites with respect to the number of brain libraries in which editing events are detected (*x*-axis). The number and percentage of editing sites are given above the bars.
(PDF)

S4 Fig Factors influencing the occurrences of A-to-I editing in the brains of *D. melanogaster*. (A) The density of editing sites is significantly positively correlated with expression level of host genes (RPKM) in each brain library. The genes expressed in brains were ranked with increasing RPKM and divided into 20 bins (the *x*-axis). In each bin, the density of editing sites (or level-weighted density) (*y*-axis) is calculated by dividing the observed number of editing sites (or weighting each site with its editing level) with the total number of adenosine sites (per million). There is a significantly positive correlation between the density of editing sites (or level-weighted density) and the gene expression level (*P* < 0.05 in each library).
(B) The density of editing sites is significantly positively correlated with mRNA-seq coverage in each brain library. All the expressed adenosine sites (≥ 5X coverage) are ranked with increasing sequencing coverage and binned into 20 categories (*x*-axis). There is a significantly positive correlation between the density of editing sites (or level-weighted density) and the mRNA-seq coverage (*P* < 0.05 in each library).
(C) The density of editing sites is significantly positively correlated with the relative distance to the transcriptional start site in each brain library. After dividing all the adenosine sites (≥ 5X coverage) into 20 equal bins along their positions in pre-mRNAs (*x*-axis), the editing density in each bin is significantly positively correlated with the relative distance to the transcriptional start sites (*P* < 0.005 in each library).
(D) mRNA-Seq coverage slightly increases towards 3' ends of mRNAs. In each brain library, after dividing the adenosine sites (≥ 5X coverage) into 20 equal bins along their positions in pre-mRNAs (*x*-axis), the median value of mRNA-Seq coverage (*y*-axis) increases along the relative position of that bin (*P* < 0.01 in each library).
(E) The density of editing is significantly higher in the rear half-gene compared to front half-gene of pre-mRNAs. Each gene is split into two equal parts (at least one half-gene had RPKM ≥ 1), and all the half-genes are grouped into 20 bins by these RPKM values. The density of editing sites is significantly higher for sites in the rear half than front half of pre-mRNAs (*P* < 0.05, for all the libraries; paired *t* tests). The ratio of the rear/front half-gene in each bin is given (*y*-axis). The red dash line indicates the ratio of 1.
(F) The density of editing sites is significantly higher for adenosine sites in the rear half than front half of pre-mRNAs. All the adenosine sites (≥ 5X coverage) are ranked with increasing coverage and binned into 20 groups. The density of editing sites is significantly higher for sites in the rear half than front half of pre-mRNAs (*P* < 0.001 in each library; paired *t* tests). The ratio of the rear/front half sites of pre-mRNAs in each bin is given (*y*-axis). The red dash line indicates the ratio of 1.
(PDF)

S5 Fig Illustration of editing sites located in secondary structures. (A) Nonsynonymous editing event (S>G) in *Adar* is located in a hairpin structure in *D*. *melanogaster*. The editing event was verified with Sanger sequencing of the female and male brains at 25°C and 30°C. Note that the editing level was reduced at 30°C in both the female and male brains. The nonsynonymous editing event (S>G) in *Adar* is located in a hairpin structure that is conserved in *D*. *simulans* and *D*. *pseudoobscura*.
(B) A-to-I editing sites in the stable hairpin structures of the pre-mRNAs of *rtp*, *DIP1*, *rdgA*, *CG43897* and *CG42540*.
(C) Sanger verification of the editing events in the hairpin structure of *rtp*, *DIP1*, *rdgA*, *CG43897* and *CG42540*. The editing sites are indicated by a blue arrow in the chromatograms above the Sanger traces.
(D) A-to-I editing events in the 3' UTR of *Adar* are located in a long-range pseudoknot in *D*. *melanogaster*, *D*. *simulans* and *D*. *pseudoobscura*. Seven editing events in the 3' UTR of *Adar* are located in a long-range pseudoknot in *D*. *melanogaster*. Six of these events are evolutionarily conserved in the long-range pseudoknot in *D*. *simulans*, and five are evolutionarily conserved in a long-range pseudoknot in *D*. *pseudoobscura*.
(PDF)

S6 Fig A-to-I editing events in long-range pseudoknots in *D. melanogaster*. (A) Abundant A-to-I editing sites are located in the stems of a long-range pseudoknot formed by an intron of *nrm* in *D*. *melanogaster*. Verification of the editing events in introns of *nrm* by Sanger sequencing the cDNA from female brains (B) and genomic DNA of *D*. *melanogaster* (C). (D) Editing events located in the stems of pre-mRNA long-range pseudoknots of *B52*, *nAchRbeta1*, *CG8034* and *roX1*.
(PDF)

S7 Fig Editing events detected in brains of *D. melanogaster* are enriched in clusters. By clustering the editing events with distances smaller than 100 nucleotides (nts), we identified a total of 1,320 editing events that form 413 clusters. The *y-axis* is the number of clusters with different number of editing sites.
(PDF)

S8 Fig A-to-I editing events clustered in the CDS of *NaCP60E* and intron of *CaMKII* in female brains of *D. melanogaster* (25°C). (A) Seven editing events are clustered in a hairpin structure in the CDS of *NaCP60E*. Editing sites are colored in red. All of these editing events were verified by Sanger sequencing the cDNA and genomic DNA.
(B) Twenty editing events in the intron of *CaMKII* (editing sites are colored in red).
(C) Verification of the 20 editing events in the intron of *CaMKII* by Sanger sequencing the cDNA (left) and genomic DNA (right).
All of the editing events are indicated by a blue arrow in the chromatograms above the Sanger traces.
(PDF)

S9 Fig Distribution of DNA-seq coverage (*x*-axis) of *D*. *simulans* (A) and *D*. *pseudoobscura* (B) used in this study.
(PDF)

S10 Fig The editing levels (*y*-axis) in brains of *D. melanogaster* are significantly higher in the sites with editing events conserved between *D. melanogaster* and *D. simulans* (*D.mel/D.sim*) or between *D. melanogaster* and *D. pseudoobscura* (*D.mel/D.pse*) compared to the sites without conserved editing events detected (Non-conserved). *, *P* < 0.05; * *, *P*< 0.01; * * *, *P* < 0.001, KS test.
(PDF)

S11 Fig The *N/S* ratios in each brain library of *D. melanogaster* with different editing level cutoffs. The editing level cutoffs used are 0, 0.1, 0.2, 0.3, 0.4 and 0.5. The *N/S* ratio under neutral evolution (3.80) is indicated with red dashed lines. (* * *, *P* < 0.001, Fisher’s exact tests).
(PDF)

S12 Fig Significantly negative correlations between the sequencing coverage (*C*) and editing level (*l*) in each brain library or pooled library (*P* < 0.001 in each case, Spearman’s correlation). (PDF)

S13 Fig The simulated and observed *N/S* ratios with increasing cutoffs of sequencing coverage (*C*_*min*_) for high-confidence editing sites in each brain library of *D. melanogaster*. The *x-*axis is the cutoff of coverage (*C*_*min*_) and the *y*-axis is the simulated (median in black, and the range from 2.5% to 97.5% quantile is in blue) and observed (red) *N/S* ratio. The *N/S* ratio under neutral evolution (3.80) is indicated with dashed lines. The cutoff of editing level, *l*_*min*_ = 0.01 at top and 0.05 at bottom.
(PDF)

S14 Fig The simulated and observed *N/S* ratios with increasing cutoffs of sequencing coverage (*C*_*min*_) for the 2,114 high confidence sites when pooling all the brain libraries of *D. melanogaster* together. Top: the *x-*axis is the cutoff of coverage (*C*_*min*_) and the *y*-axis is the simulated (median in black, and the range from 2.5% to 97.5% quantile is in blue) and observed (red) *N/S* ratio. The *N/S* ratio under neutral evolution (3.80) is indicated with dashed lines. Left: *l*_*min*_ = 0.01; Middle: *l*_*min*_ = 0.02; Right: *l*_*min*_ = 0.05. The corresponding relative differences (the simulated/observed *N/S* ratio) for each simulation is given at the bottom panel.
(PDF)

S15 Fig Comparison of *N/S* ratio of X-linked and autosomal editing sites. (A) The observed *N/S* ratios for the X-linked and autosomal editing sites in all brain libraries of *D*. *melanogaster*. The expected *N/S* ratios for X chromosome and autosomes are presented in lines. For both X-linked and autosomal editing sites, the observed *N/S* ratios are significantly higher than neutral expectation (*, *P* < 0.05; * *, *P*< 0.01; ***, *P* < 0.001; Fisher’s exact tests).
(B) *N/S* ratios for the X-linked and autosomal editing sites with events observed in brains of both *D*. *simulans* and the matched sample of *D*. *melanogaster*. The expected *N/S* ratios are presented in lines. For both X-linked and autosomal editing sites, the observed *N/S* ratios are significantly higher than neutral expectation (*, *P* < 0.05; * *, *P*< 0.01; ***, *P* < 0.001; Fisher’s exact tests).
(PDF)

S16 Fig Boxplots showing the expression levels (RPKM) of PSEB are significantly higher than the non-PSEB genes in each of eight brain libraries of *D. melanogaster* (***, *P* < 0.001; KS tests). (PDF)

S17 Fig Boxplots showing the editing levels of PSEB and non-PSEB genes in each of eight brain libraries of *D. melanogaster*. (PDF)

S18 Fig The editing density in the nonsynonymous adenosine sites is significantly lower in the non-conserved (low phyloP scores) than the conserved (high phyloP scores) DNA sites after controlling mRNA-Seq coverage. All the nonsynonymous adenosine sites (cause amino acid changes if edited; ≥ 5X coverage) are ranked with increasing sequencing coverage and binned into 20 categories (*x-axis*). Within each bin, the sites are divided into two equal-sized subgroups based on the phyloP scores. The *y-axis* is the editing density of the non-conserved relative to the conserved subgroup in each bin (*P* < 0.001 in each comparison, paired *t* test).
(PDF)

S19 Fig The editing density in the synonymous adenosine sites is significantly lower in the non-conserved (low phyloP scores) than the conserved (high phyloP scores) DNA sites after controlling mRNA-Seq coverage. All the synonymous adenosine sites (do not cause amino acid changes if edited; ≥ 5X coverage) are ranked with increasing sequencing coverage and binned into 20 categories (*x-axis*). Within each bin, the sites are divided into two equal-sized subgroups based on the phyloP scores. The *y-axis* is the editing density of the non-conserved relative to the conserved subgroup in each bin (*P* < 0.001 in each comparison, paired *t* test).
(PDF)

S20 Fig Distribution of mRNA-seq coverage of exonic region in female and male adults from five *D. melanogaster* strains. (PDF)

S21 Fig The simulated and observed *N/S* ratios with increasing cutoffs of sequencing coverage (*C*_*min*_) for editing sites in each library of the five strains of *D. melanogaster*. The *x-*axis is the cutoff of coverage (*C*_*min*_) and the *y*-axis is the simulated (median in black, and the range from 2.5% to 97.5% quantile is in blue) and observed (red) *N/S* ratio. The *N/S* ratio under neutral evolution (3.80) is indicated with dashed lines. The corresponding relative differences (the simulated/observed *N/S* ratio) for each simulation is given at the bottom panel. The editing level cutoffs *l*_*min*_ = 0.01 and 0.05 are used in the simulations.
(PDF)

S22 Fig The changes of editing levels in all sites under elevated temperature are significantly positively correlated between brains of *D. melanogaster* and *D. simulans*. (PDF)

S23 Fig Clustering the brain libraries based on editing levels. (A). Clustering the brain libraries of *D*. *melanogaster* based on the editing levels of high-confidence editing sites that have at least 15 (left) or 25 (right) raw reads in each brain library. Note flies of the same accommodation conditions always cluster together.
(B). Clustering the brain libraries of *D*. *melanogaster* and *D*. *pseudoobscura* based on the editing levels of 152 high-confidence editing sites that have at least 20 raw reads in each brain library. Note species divergence plays a more important role than temperature in clustering the samples.
(PDF)

S24 Fig The changes in editing levels of the *N* sites are weakly but significantly positively correlated with the changes in expression levels of the host genes under elevated temperature for 48 hours. In contrast, no significant patterns are observed for the *S* sites (RPKM cutoff of 1, 3, 5, or 10 was used).
(PDF)

S25 Fig Expression of *Adar* was down-regulated after increasing the temperature from 25°C to 30°C as detected with qPCR. The error bars are s.e..
(PDF)

S26 Fig The *N/S* ratios in different developmental stages of *D. melanogaster*. The *N/S* ratios (*x*-axis) for all the editing sites in different developmental stages of *D*. *melanogaster* in the modENCODE Project re-analyzed by Ramaswami *et al*. The overall observed *N/S* ratios are compared to the expected *N/S* ratio under neutral evolution (3.80): *, *P* < 0.05; **, *P* < 0.01; ***, *P* < 0.001.
(PDF)

S27 Fig The *N/S* ratios of editing sites in PSEB and non-PSEB genes in different developmental stages of *D. melanogaster*, stratified by gene expression levels. The *N/S* ratios (*x*-axis) for editing sites in PSEB genes (red) or non-PSEB genes (blue) that are Highly expressed (Left panel) or Lowly expressed (Right panel) in our brain data and different developmental stages of *D*. *melanogaster* in the modENCODE Project. Asterisks indicate significant difference in *N/S* ratios between PSEB and non-PSEB editing sites: *, *P* < 0.05; **, *P* < 0.01; ***, *P* < 0.001.
(PDF)

S28 Fig Heatmap showing the editing levels of editing sites in PSEB and non-PSEB genes in different developmental stages of *D. melanogaster* in the modENCODE Project. (PDF)

S29 Fig Relationship between the expression level of *Adar* and the number or cumulative level of editing sites. (A) The expression level of *Adar* is significantly positively correlated with the number of editing sites detected in our brain data and different developmental stages of *D*. *melanogaster* in the modENCODE Project. Spearman’s correlation coefficient *rho* was calculated and displayed in the plot.
(B) The expression level of *Adar* is significantly positively correlated with the cumulative editing level of editing sites detected in our brain data and different developmental stages of *D*. *melanogaster* in the modENCODE Project. Spearman’s correlation coefficient *rho* was calculated and displayed in the plot.
(PDF)

S30 Fig The number of editing sites detected in our brain data different developmental stages of *D. melanogaster* in the modENCODE Project. (PDF)

S31 Fig Boxplots of the *dN* and *dS* values between *D. melanogaster* and *D. simulans* for the genes that have (Edited) or do not have editing events in CDS regions (Unedited) in *Drosophila* brains. “Total” means the whole CDS regions; “Structured” means the CDS regions which form secondary structures and harbor editing events; and “Structure masked” means the remaining CDS regions which are outside secondary structures of mRNAs.
(PDF)

S32 Fig Editing events in *D. melanogaster* that compensated for the G-to-A DNA mutations. A-to-I RNA editing events in *D*. *melanogaster* that compensated for the G-to-A DNA mutation in the *D*. *melanogaster* lineage after splitting with its sibling species. The DNA sequences of *D*. *simulans* and *D*. *yakuba* are used as outgroups.
(PDF)
