# Supplementary material for: Adaptation of A-to-I RNA editing in Drosophila
Source: PLoS Genet. 2017 Mar 10;13(3):e1006648. doi: 10.1371/journal.pgen.1006648 (PMC5365144; doi:10.1371/journal.pgen.1006648)
Supplement: S1 Table — D. mel, D. melanogaster; D. sim, D. simulans; D.pse, D. pseudoobscura. F: female; M: male. All flies are raised at 25°C. 30°C, 14h: the flies was raised at 25°C, and treated at 30°C for 14 hours; 30°C, 48h: the flies were raised at 25°C and treated at 30°C for 48 hours. (PDF) [file pgen.1006648.s001.pdf]

Species	Sex	Age (day)	Temp	Tissue	Sites⩾5X coverage (million)	Total reads (million)	Mapping rate (%)	Unique mapping rate (%)
**Brains of three *Drosophila* species**								
D. mel	F	1-14	25°C	brain	18.43	22.36	93.15	78.33
D. mel	M	1-14	25°C	brain	18.37	23.31	92.43	69.69
D. mel	F	1-5	25°C	brain	13.10	15.78	88.20	63.31
D. mel	F	1-5	30°C,14h	brain	14.46	16.64	90.85	69.52
D. mel	F	1-5	30 °C, 48h	brain	14.97	18.48	89.79	66.95
D. mel	M	1-5	25°C	brain	15.68	17.67	91.45	66.15
D. mel	M	1-5	30°C, 14h	brain	16.45	17.89	91.48	64.60
D. mel	M	1-5	30°C, 48h	brain	17.13	16.59	90.18	74.32
D. sim	F	1-5	25°C	brain	11.00	15.32	56.66	54.92
D. sim	F	1-5	30°C, 14h	brain	12.67	13.58	66.80	64.81
D. sim	F	1-5	30°C, 48h	brain	15.47	21.77	61.95	59.84
D. sim	M	1-5	25°C	brain	14.59	18.12	65.31	63.12
D. sim	M	1-5	30°C, 14h	brain	17.19	24.37	67.35	65.18
D. sim	M	1-5	30°C, 48h	brain	17.08	24.08	63.51	61.37
D. sim	F	1-5	25°C	body	13.33	21.64	84.13	59.66
D. sim	F	1-5	25°C	head	15.41	17.13	85.76	76.29
D. sim	M	1-5	25°C	body	14.80	21.54	85.66	69.25
D. sim	M	1-5	25°C	head	16.04	17.72	87.42	80.13
D. pse	F	1-5	25°C	brain	12.62	15.67	69.55	56.52
D. pse	F	1-5	30°C, 14h	brain	13.76	15.71	74.20	57.56
D. pse	F	1-5	30°C, 48h	brain	15.92	18.18	75.30	62.40
D. pse	M	1-5	25°C	brain	16.34	25.59	61.87	48.03
D. pse	M	1-5	30°C, 14h	brain	18.36	22.37	79.48	65.94
D. pse	M	1-5	30°C, 48h	brain	17.70	19.99	78.78	68.02
**Adults of five strains of *D. melanogaster***								
B12	F	1-14	25°C	whole body	49.29	23.34	95.82	87.12
B12	M	1-14	25°C	whole body	57.32	26.94	96.82	91.12
I17	F	1-14	25°C	whole body	44.85	17.61	96.83	90.81
I17	M	1-14	25°C	whole body	49.49	15.93	96.69	92.74
N10	F	1-14	25°C	whole body	42.33	18.07	97.74	91.80
N10	M	1-14	25°C	whole body	51.17	17.65	97.95	92.20
T07	F	1-14	25°C	whole body	44.68	22.54	97.44	93.02
T07	M	1-14	25°C	whole body	27.25	18.99	98.51	92.04
ZW155	F	1-14	25°C	whole body	44.17	29.49	96.85	91.34
ZW155	M	1-14	25°C	whole body	54.97	26.16	96.48	85.13
