# Supplementary material for: Adaptation of A-to-I RNA editing in Drosophila
Source: PLoS Genet. 2017 Mar 10;13(3):e1006648. doi: 10.1371/journal.pgen.1006648 (PMC5365144; doi:10.1371/journal.pgen.1006648)
Supplement: S3 Table — The FDR is calculated based on the multiple test correction of Joint P(E0) with Benjamini & Hochberg method. For each site, we counted the number of brain libraries in which editing was detected at that site. The other criteria in defining Class I and II sites are the same as described in TEXT. (PDF) [file pgen.1006648.s003.pdf]

Number of libraries / *Q* value	1	2	3	4	5
Class I					
0.05	1,762	1,760	1,702	1,578	1,424
0.01	1,736	1,734	1,692	1,575	1,422
0.001	1,703	1,702	1,679	1,574	1,422
0.0001	1,677	1,676	1,662	1,570	1,421
0.00001	1,638	1,638	1,632	1,557	1,419
Class I + II					
0.05	2,191	2,185	2,073	1,881	1,664
0.01	2,152	2,149	2,063	1,878	1,662
0.001	2,095	2,094	2,038	1,877	1,662
0.0001	2,041	2,040	2,006	1,873	1,661
0.00001	1,993	1,993	1,969	1,857	1,659
