# Supplementary material for: Adaptation of A-to-I RNA editing in Drosophila
Source: PLoS Genet. 2017 Mar 10;13(3):e1006648. doi: 10.1371/journal.pgen.1006648 (PMC5365144; doi:10.1371/journal.pgen.1006648)
Supplement: S4 Table — (PDF) [file pgen.1006648.s004.pdf]

Studies	Graveley *et al.* 2011	Yu *et al.* 2016	St.Laurant *et al.* 2013	Rodriguez *et al.* 2012	This study
Graveley *et al.* 2011	972 (100%)	345 (26.58%)	458 (12.79%)	317 (23.48%)	461 (21.81%)
Yu *et al.* 2016	345 (35.49%)	1298 (100%)	497 (13.88%)	361 (26.74%)	595 (28.15%)
St.Laurant *et al.* 2013	458 (47.12%)	497 (38.29%)	3581 (100%)	739 (54.74%)	1292 (61.12%)
Rodriguez *et al.* 2012	317 (32.61%)	361 (27.81%)	739 (20.64%)	1350 (100%)	776 (36.71%)
This study	461 (47.43%)	595 (45.84%)	1292 (36.08%)	776 (57.48%)	2114 (100%)
