# Supplementary material for: Adaptation of A-to-I RNA editing in Drosophila
Source: PLoS Genet. 2017 Mar 10;13(3):e1006648. doi: 10.1371/journal.pgen.1006648 (PMC5365144; doi:10.1371/journal.pgen.1006648)
Supplement: S5 Table — The criteria in identifying editing site in each single library and the annotation are described in Table 1. (PDF) [file pgen.1006648.s005.pdf]

Library	5' UTR	3' UTR	Intron	ncRNA	Intergenic	PSEB				non-PSEB			
						N	*S*	*N/S* ratio (95% CI)	*P* value	*N*	*S*	*N/S* ratio (95% CI)	*P* value
B1	10	243	261	27	121	245	6	40.8 (21.8, 124)	4.5×10^{-18}	148	76	1.95 (1.49, 2.61)	3.6×10^{-6}
B2	3	176	175	17	66	180	2	90.0 (35.4, 182)	4.4×10^{-16}	120	46	2.61 (1.91, 3.74)	0.021
B3	2	170	168	25	64	215	2	108 (42.4, 217)	1.8×10^{-19}	125	56	2.23 (1.66, 3.11)	8.8×10^{-4}
B4	4	146	150	27	54	210	4	52.5 (25.8, 213)	9.2×10^{-17}	120	49	2.45 (1.77, 3.45)	7.3×10^{-3}
B5	9	265	284	42	144	288	5	57.6 (28.3, 292)	4.9×10^{-23}	160	80	2.00 (1.55, 2.64)	4.4×10^{-6}
B6	4	208	200	37	87	235	3	78.3 (33.0, 238)	3.3×10^{-20}	129	56	2.30 (1.68, 3.20)	1.6×10^{-3}
B7	2	188	266	39	89	273	3	91.0 (38.4, 276)	7.3×10^{-24}	153	76	2.01 (1.54, 2.69)	8.8×10^{-6}
B8	7	189	223	37	97	255	4	63.8 (31.4, 258)	5.5×10^{-21}	148	65	2.28 (1.73, 3.10)	5.5×10^{-4}
Total	20	414	550	42	235	447	8	55.9 (31.5, 151)	8.8×10^{-35}	231	136	1.70 (1.38, 2.11)	7.0×10^{-13}
