# Supplementary material for: Adaptation of A-to-I RNA editing in Drosophila
Source: PLoS Genet. 2017 Mar 10;13(3):e1006648. doi: 10.1371/journal.pgen.1006648 (PMC5365144; doi:10.1371/journal.pgen.1006648)
Supplement: S6 Table — (PDF) [file pgen.1006648.s006.pdf]

| GO Term | Count | Percentage (%) | *P* value | *Q* value |
| --- | --- | --- | --- | --- |
| neurotransmitter secretion | 23 | 4.19 | $1.90\times10^{-9}$ | $3.09\times10^{-6}$ |
| synaptic vesicle | 17 | 3.10 | $2.64\times10^{-7}$ | $3.57\times10^{-4}$ |
| cell junction | 22 | 4.01 | $2.40\times10^{-11}$ | $3.24\times10^{-8}$ |
| postsynaptic membrane | 15 | 2.73 | $5.43\times10^{-8}$ | $7.33\times10^{-5}$ |
| potassium ion transport | 12 | 2.19 | $4.56\times10^{-8}$ | $7.45\times10^{-5}$ |
| calcium ion transmembrane transport | 9 | 1.64 | $5.28\times10^{-7}$ | $8.61\times10^{-4}$ |
