# Supplementary material for: Adaptation of A-to-I RNA editing in Drosophila
Source: PLoS Genet. 2017 Mar 10;13(3):e1006648. doi: 10.1371/journal.pgen.1006648 (PMC5365144; doi:10.1371/journal.pgen.1006648)
Supplement: S7 Table — (PDF) [file pgen.1006648.s007.pdf]

GO Term	Count	Percentage (%)	*P* value	*Q* value
neurotransmitter secretion	22	4.15	5.19×10^{-9}	8.44×10^{-6}
synaptic vesicle	16	3.02	9.45×10^{-7}	0.00127
potassium ion transport	11	2.08	3.81×10^{-7}	6.19×10^{-4}
postsynaptic membrane	14	2.64	2.80×10^{-7}	3.76×10^{-4}
calcium ion transmembrane transport	9	1.70	3.81×10^{-7}	6.20×10^{-4}
