# Supplementary material for: Adaptation of A-to-I RNA editing in Drosophila
Source: PLoS Genet. 2017 Mar 10;13(3):e1006648. doi: 10.1371/journal.pgen.1006648 (PMC5365144; doi:10.1371/journal.pgen.1006648)
Supplement: S8 Table — (PDF) [file pgen.1006648.s008.pdf]

Gene ID	Gene name	Total	*N*	*S*	5'UTR	3'UTR	Intron	Others
FBgn0264255	*para*	50	19	5	0	0	21	5
FBgn0264607	*CaMKII*	47	0	0	0	0	47	0
FBgn0050428	*CG30428*	46	0	0	0	0	0	46
FBgn0041605	*cpx*	37	0	0	0	37	0	0
FBgn0028875	*nAChRalpha5*	34	9	4	0	0	1	20
FBgn0262509	*nrm*	33	0	0	0	0	33	0
FBgn0260657	*CG42540*	32	1	1	0	20	10	0
FBgn0263111	*cac*	27	12	1	0	0	14	0
FBgn0085434	*NaCP60E*	25	18	5	0	2	0	0
FBgn0262593	*Shab*	25	13	5	0	0	7	0
FBgn0263354	*CG42784*	23	2	0	0	0	21	0
FBgn0264386	*Ca-alpha1T*	22	13	8	0	1	0	0
FBgn0032151	*nAChRalpha6*	20	7	1	0	0	12	0
FBgn0042174	*CR18854*	18	0	0	0	0	0	18
FBgn0085432	*pan*	18	0	0	0	0	16	2
FBgn0003380	*Sh*	17	3	0	0	0	8	6
FBgn0039536	*unc80*	16	14	2	0	0	0	0
FBgn0004242	*Syt1*	15	4	0	0	0	11	0
FBgn0260499	*qvr*	15	4	3	0	8	0	0
FBgn0019661	*roX1*	14	0	0	0	0	0	14
FBgn0026056	*Rlip*	14	10	0	0	4	0	0
FBgn0058263	*MFS17*	14	0	0	0	0	14	0
FBgn0261549	*rdgA*	14	0	0	0	0	14	0
FBgn0000038	*nAChRbeta1*	13	2	2	0	0	9	0
FBgn0000535	*eag*	13	10	3	0	0	0	0
FBgn0003429	*slo*	13	4	0	0	8	1	0
FBgn0004244	*Rdl*	13	4	2	0	2	5	0
FBgn0004607	*zfh2*	13	0	0	0	0	0	13
FBgn0024232	*gprs*	13	3	2	0	8	0	0
FBgn0262614	*pyd*	13	1	0	0	0	12	0
FBgn0024807	*DIP1*	12	0	0	0	0	12	0
FBgn0033403	*CG13739*	12	3	0	0	9	0	0
FBgn0039927	*CG11155*	12	0	0	0	0	10	2
FBgn0001991	*Ca-alpha1D*	11	5	2	0	0	4	0
FBgn0016696	*Pitslre*	11	0	0	0	0	11	0
FBgn0023535	*arg*	11	0	0	0	0	11	0
FBgn0035720	*CG10077*	11	1	1	0	8	1	0
FBgn0039920	*CG11360*	11	0	0	0	11	0	0
FBgn0259994	*CG42492*	11	10	1	0	0	0	0
FBgn0264489	*CG43897*	11	0	0	0	11	0	0
FBgn0004575	*Syn*	10	4	1	0	0	5	0
FBgn0010473	*tutl*	10	4	1	0	0	5	0
FBgn0011286	*RyR*	10	9	1	0	0	0	0
FBgn0013995	*Calx*	10	5	0	2	0	3	0
FBgn0026086	*Adar*	10	1	0	0	9	0	0
FBgn0053653	*Cadps*	10	1	0	0	0	9	0
FBgn0261041	*stj*	10	8	0	0	0	2	0
FBgn0024987	*ssx*	9	1	1	0	7	0	0
FBgn0030897	*Frq1*	9	1	2	0	6	0	0
FBgn0038659	*EndoA*	9	5	1	0	3	0	0
