# Supplementary material for: Adaptation of A-to-I RNA editing in Drosophila
Source: PLoS Genet. 2017 Mar 10;13(3):e1006648. doi: 10.1371/journal.pgen.1006648 (PMC5365144; doi:10.1371/journal.pgen.1006648)
Supplement: S12 Table — The criteria in identifying editing site in each single library and the annotation are described in Table 3. Please note, for each site, we require the editing events to be present in the matched brain samples of D. melanogaster and D. pseudoobscura. (PDF) [file pgen.1006648.s012.pdf]

Library	5’UTR	3’UTR	Intron	PSEB				non-PSEB			
				N	*S*	*N/S* ratio (95% CI)	*P* value	*N*	*S*	*N/S* ratio (95% CI)	*P* value
P2	0 (0%)	4 (2.3%)	12 (6.9%)	86	0	Inf	1.2×10^{-5}	56	13	4.31 (2.45, 8.86)	0.08
P3	0 (0%)	5 (2.9%)	14 (8.3%)	89	0	Inf	8.2×10^{-6}	58	14	4.14 (2.43, 8.00)	0.06
P4	0 (0%)	4 (2.7%)	10 (6.7%)	80	0	Inf	2.7×10^{-5}	63	14	4.50 (2.67, 8.63)	0.09
P6	0 (0%)	7 (3.4%)	19 (9.5%)	106	1	106 (34.7, 107)	1.2×10^{-5}	75	16	4.69 (2.79, 9.11)	0.09
P7	0 (0%)	7 (3.7%)	21 (7.9%)	106	1	106 (34.7, 107)	1.2×10^{-5}	84	21	4.00 (2.62, 7.08)	0.02
P8	0 (0%)	8 (4.2%)	15 (6.7%)	100	0	Inf	1.9×10^{-6}	81	19	4.26 (2.70, 7.33)	0.04
Total events	0 (0%)	15 (3.6%)	42 (7.6%)	107	24	4.46 (2.97, 7.73)	0.98	218	24	9.08 (6.33, 14.13)	0.90
