# Supplementary material for: Adaptation of A-to-I RNA editing in Drosophila
Source: PLoS Genet. 2017 Mar 10;13(3):e1006648. doi: 10.1371/journal.pgen.1006648 (PMC5365144; doi:10.1371/journal.pgen.1006648)
Supplement: S13 Table — (PDF) [file pgen.1006648.s013.pdf]

ID_dm6	ID_dsim1.4	Total Reads in six libraries of *D. simulans*	Total G alleles in six libraries of *D. simulans*
2L:14077162	2L:13831847	74	2
2L:2785536	2L:2742132	18	0
2L:8177946	2L:7960627	9	1
2R:16647491	2R:11268649	4	0
2R:24912004	2R:19252139	18	1
2R:6725344	2R:1426698	44	1
2R:8883745	2R:3444217	1	1
3L:20773610	3L:20122767	65	0
3R:11801272	3R:13764618	55	0
3R:19765660	3R:5893305	107	0
3R:25965631	3R:21638185	125	1
3R:29741419	3R:25239596	45	0
X:1491593	X:1047982	17	0
X:14999920	X:11494927	16	1
X:14999938	X:11494945	16	1
X:16475638	X:12581248	28	1
X:16475685	X:12581295	50	2
X:1785903	chrX_Mrandom_123:184	43	0
X:19729029	X:15115286	169	0
