# Supplementary material for: Adaptation of A-to-I RNA editing in Drosophila
Source: PLoS Genet. 2017 Mar 10;13(3):e1006648. doi: 10.1371/journal.pgen.1006648 (PMC5365144; doi:10.1371/journal.pgen.1006648)
Supplement: S14 Table — In each library and category, we adjust the numbers of N sites by dividing them with the over-estimation ratios (the median simulated N/S ratio divided by the observed N/S ratio) obtained from simulation. The numbers of adjusted N sites (N’) are given. (PDF) [file pgen.1006648.s014.pdf]

Library	Total				PSEB				non-PSEB			
	N'	*S*	*N'/S*	*P* value	*N'*	*S*	*N'/S*	*P* value	*N'*	*S*	*N'/S*	*P* value
B1	387	81	4.78 (3.82, 6.20)	0.03	240	6	40.0 (21.4, 122)	1.3×10^{-17}	148	76	1.95 (1.49,2.61)	3.6×10^{-6}
B2	300	48	6.25 (4.70, 8.67)	4.9×10^{-4}	180	2	90.0 (35.4, 182)	4.4×10^{-16}	120	46	2.61 (1.91,3.74)	0.02
B3	340	58	5.86 (4.53, 7.84)	9.2×10^{-4}	215	2	108 (42.4, 217)	1.8×10^{-19}	125	56	2.231 (1.66,3.11)	8.8×10^{-4}
B4	330	53	6.23 (4.80, 8.58)	2.9×10^{-4}	210	4	52.5 (25.8, 213)	9.2×10^{-17}	120	49	2.45 (1.77,3.45)	7.3×10^{-3}
B5	484	94	5.15 (4.16, 6.51)	3.4×10^{-3}	303	5	60.6 (29.80, 307)	1.9×10^{-24}	160	80	2 (1.53,2.64)	4.4×10^{-6}
B6	364	59	6.17 (4.79, 8.40)	1.8×10^{-4}	235	3	78.3 (33.0, 238)	3.3×10^{-20}	129	56	2.30 (1.72,3.20)	1.6×10^{-3}
B7	426	79	5.39 (4.32, 7.02)	2.0×10^{-3}	273	3	91.0 (38.4, 276)	7.3×10^{-24}	153	76	2.01 (1.54,2.69)	8.8×10^{-6}
B8	403	69	5.84 (4.62, 7.74)	3.7×10^{-4}	255	4	63.75 (31.4, 258)	5.5×10^{-21}	148	65	2.28 (1.73,3.10)	5.5×10^{-4}
Total	662	140	4.73 (3.98, 5.74)	9.9×10^{-3}	435	8	54.4 (30.6, 147)	1.2×10^{-33}	231	136	1.70 (1.38,2.11)	7.0×10^{-13}
