# Supplementary material for: Adaptation of A-to-I RNA editing in Drosophila
Source: PLoS Genet. 2017 Mar 10;13(3):e1006648. doi: 10.1371/journal.pgen.1006648 (PMC5365144; doi:10.1371/journal.pgen.1006648)
Supplement: S16 Table — (PDF) [file pgen.1006648.s016.pdf]

Library	Highly expressed PSEB			Highly expressed non-PSEB			*P* value	Lowly expressed PSEB			Lowly expressed non-PSEB			*P* value
	N	*S*	*N/S*	*N*	*S*	*N/S*		*N*	*S*	*N/S*	*N*	*S*	*N/S*	
B1	137	3	45.7	72	36	2.00	5.4×10^{-12}	108	3	36.0	76	40	1.90	1.2×10^{-10}
B2	91	1	91.0	61	27	2.26	6.9×10^{-9}	89	1	89.0	59	19	3.11	2.8×10^{-6}
B3	116	1	116	62	27	2.30	1.9×10^{-10}	99	1	99.0	63	29	2.17	9.4×10^{-10}
B4	107	2	53.5	71	27	2.63	3.1×10^{-8}	103	2	51.5	49	22	2.23	2.9×10^{-8}
B5	154	2	77.0	86	47	1.83	3.5×10^{-16}	134	3	44.7	74	33	2.24	2.1×10^{-10}
B6	124	2	62.0	64	33	1.94	7.9×10^{-12}	111	1	111.0	65	23	2.83	1.6×10^{-8}
B7	147	2	73.5	80	42	1.90	1.1×10^{-14}	126	1	126.0	73	34	2.15	2.2×10^{-12}
B8	137	3	45.7	80	38	2.11	8.0×10^{-12}	118	1	118.0	68	27	2.52	5.0×10^{-10}
Pool	250	7	35.7	130	77	1.69	1.8×10^{-23}	197	1	197.0	101	59	1.71	6.6×10^{-23}
