# Supplementary material for: Adaptation of A-to-I RNA editing in Drosophila
Source: PLoS Genet. 2017 Mar 10;13(3):e1006648. doi: 10.1371/journal.pgen.1006648 (PMC5365144; doi:10.1371/journal.pgen.1006648)
Supplement: S17 Table — (PDF) [file pgen.1006648.s017.pdf]

Base	-3	-2	-1	0	+1	+2	+3
A	605	567	752	2114	427	556	562
C	423	553	466	0	370	503	470
G	565	494	126	0	849	476	460
T	521	500	770	0	468	579	622
