# Supplementary material for: Adaptation of A-to-I RNA editing in Drosophila
Source: PLoS Genet. 2017 Mar 10;13(3):e1006648. doi: 10.1371/journal.pgen.1006648 (PMC5365144; doi:10.1371/journal.pgen.1006648)
Supplement: S18 Table — (PDF) [file pgen.1006648.s018.pdf]

Base	-3	-2	-1	0	+1	+2	+3
A	0.286	0.268	0.356	1.000	0.202	0.263	0.266
C	0.200	0.262	0.220	0.000	0.175	0.238	0.222
G	0.267	0.234	0.060	0.000	0.402	0.225	0.218
T	0.246	0.237	0.364	0.000	0.221	0.274	0.294
