# Supplementary material for: Adaptation of A-to-I RNA editing in Drosophila
Source: PLoS Genet. 2017 Mar 10;13(3):e1006648. doi: 10.1371/journal.pgen.1006648 (PMC5365144; doi:10.1371/journal.pgen.1006648)
Supplement: S19 Table — The score cutoff is -0.6216; the number of background adenosines is 4045312. The percentage of the triplets in each category is in the parenthesis. (PDF) [file pgen.1006648.s019.pdf]

Triplet	High-Confidence editing sites			Background adenosine sites		
	Total	Below cutoff	Above cutoff	Total	Below cutoff	Above cutoff
AAA	180 (8.5%)	0 (0.0%)	180 (9.5%)	558,721 (13.8%)	0 (0.0%)	558,721 (18.3%)
AAC	121 (5.7%)	1 (0.5%)	120 (6.3%)	256,067 (6.3%)	3,391 (0.3%)	252,676 (8.3%)
AAG	298 (14.1%)	0 (0.0%)	298 (15.7%)	242,634 (6.0%)	0 (0.0%)	242,634 (8.0%)
AAT	153 (7.2%)	0 (0.0%)	153 (8.0%)	385,798 (9.5%)	0 (0.0%)	385,798 (12.9%)
CAA	78 (3.7%)	31 (14.6%)	47 (2.5%)	323,064 (8.0%)	81,930 (8.2%)	241,134 (7.9%)
CAC	75 (3.5%)	43 (20.3%)	32 (1.7%)	202,234 (5.0%)	118,420 (11.9%)	83,814 (2.7%)
CAG	253 (12.0%)	0 (0.0%)	253 (13.3%)	207,003 (5.1%)	0 (0.0%)	207,003 (6.8%)
CAT	60 (2.8%)	10 (4.7%)	50 (2.6%)	237,360 (5.9%)	37,187 (3.7%)	200,173 (6.6%)
GAA	24 (1.1%)	24 (11.3%)	0 (0.0%)	266,220 (6.6%)	266,220 (26.8%)	0 (0.0%)
GAC	12 (0.6%)	12 (5.7%)	0 (0.0%)	131,482 (3.3%)	131,482 (13.2%)	0 (0.0%)
GAG	67 (3.2%)	67 (31.6%)	0 (0.0%)	165,208 (4.1%)	165,208 (16.8%)	0 (0.0%)
GAT	23 (1.1%)	23 (10.8%)	0 (0.0%)	187,980 (4.6%)	187,980 (18.9%)	0 (0.0%)
TAA	145 (6.9%)	0 (0.0%)	145 (7.6%)	295,446 (7.3%)	0 (0.0%)	295,446 (9.7%)
TAC	162 (7.7%)	1 (0.5%)	161 (8.5%)	156,917 (3.9%)	1,537 (0.2%)	155,380 (5.1%)
TAG	231 (10.9%)	0 (0.0%)	231 (12.1%)	130,218 (3.2%)	0 (0.0%)	130,218 (4.3%)
TAT	232 (11.0%)	0 (0.0%)	232 (12.2%)	298,960 (7.4%)	0 (0.0%)	298,960 (9.8%)
