# Supplementary material for: Adaptation of A-to-I RNA editing in Drosophila
Source: PLoS Genet. 2017 Mar 10;13(3):e1006648. doi: 10.1371/journal.pgen.1006648 (PMC5365144; doi:10.1371/journal.pgen.1006648)
Supplement: S20 Table — (PDF) [file pgen.1006648.s020.pdf]

Library	Gender	Age (d)	Temp	rho(*N*)	*P*(*N*)	rho(*S*)	*P*(*S*)
B1	F	1-14	25°C	-0.824	3.84×10^{-6}	-0.56	1.02×10^{-2}
B2	F	1-5	25°C	-0.734	3.45×10^{-4}	-0.497	2.58×10^{-2}
B3	F	1-5	30°C, 14h	-0.758	1.65×10^{-4}	-0.42	6.51×10^{-2}
B4	F	1-5	30°C, 48h	-0.768	1.14×10^{-4}	-0.402	7.88×10^{-2}
B5	M	1-14	25°C	-0.795	3.60×10^{-5}	-0.557	1.07×10^{-2}
B6	M	1-5	25°C	-0.792	4.18×10^{-5}	-0.437	5.42×10^{-2}
B7	M	1-5	30°C, 14h	-0.774	9.10×10^{-5}	-0.476	3.39×10^{-2}
B8	M	1-5	30°C, 48h	-0.765	1.27×10^{-4}	-0.45	4.63×10^{-2}
