# Supplementary material for: Adaptation of A-to-I RNA editing in Drosophila
Source: PLoS Genet. 2017 Mar 10;13(3):e1006648. doi: 10.1371/journal.pgen.1006648 (PMC5365144; doi:10.1371/journal.pgen.1006648)
Supplement: S21 Table — (PDF) [file pgen.1006648.s021.pdf]

Library	Gender	Age (d)	Temp	rho(*N*)	*P*(*N*)	rho(*S*)	*P*(*S*)
B1	F	1-14	25°C	0.792	4.18×10^{-5}	0.738	3.03×10^{-4}
B2	F	1-5	25°C	0.802	2.60×10^{-5}	0.618	4.45×10^{-3}
B3	F	1-5	30°C, 14h	0.832	2.99×10^{-7}	0.519	2.06×10^{-2}
B4	F	1-5	30°C, 48h	0.711	6.31×10^{-4}	0.609	5.22×10^{-3}
B5	M	1-14	25°C	0.786	5.53×10^{-5}	0.702	7.88×10^{-4}
B6	M	1-5	25°C	0.783	6.31×10^{-5}	0.62	4.34×10^{-3}
B7	M	1-5	30°C, 14h	0.732	3.60×10^{-4}	0.684	1.20×10^{-3}
B8	M	1-5	30°C, 48h	0.753	1.91×10^{-4}	0.58	8.36×10^{-3}
