# Supplementary material for: Adaptation of A-to-I RNA editing in Drosophila
Source: PLoS Genet. 2017 Mar 10;13(3):e1006648. doi: 10.1371/journal.pgen.1006648 (PMC5365144; doi:10.1371/journal.pgen.1006648)
Supplement: S24 Table — (PDF) [file pgen.1006648.s024.pdf]

Strain	Highly expressed PSEB			Highly expressed non-PSEB			*P* value	Lowly expressed PSEB			Lowly expressed non-PSEB			*P* value
	N	*S*	*N/S*	*N*	*S*	*N/S*		*N*	*S*	*N/S*	*N*	*S*	*N/S*	
Female														
B12	83	6	13.83	76	40	1.90	1.3×10^{-6}	136	11	12.36	64	35	1.83	7.4×10^{-8}
I17	74	2	37.00	59	31	1.90	8.8×10^{-8}	95	6	15.83	54	34	1.59	2.7×10^{-8}
N10	50	1	50.00	68	30	2.27	1.1×10^{-5}	94	4	23.50	55	30	1.83	5.3×10^{-8}
T07	55	2	27.50	79	34	2.32	1.9×10^{-5}	99	5	19.80	70	37	1.89	2.7×10^{-8}
ZW155	55	2	27.50	93	33	2.82	1.7×10^{-4}	111	6	18.50	73	44	1.66	6.8×10^{-10}
Pool	83	5	16.60	127	61	2.08	2.9×10^{-7}	152	15	10.13	124	70	1.77	5.3×10^{-10}
Male														
B12	147	19	7.74	164	91	1.80	1.6×10^{-8}	209	23	9.09	153	69	2.22	2.2×10^{-8}
I17	114	9	12.67	90	61	1.48	1.1×10^{-10}	147	14	10.50	121	63	1.92	6.1×10^{-9}
N10	98	11	8.91	103	64	1.61	1.0×10^{-7}	158	10	15.80	111	57	1.95	7.0×10^{-11}
T07	113	12	9.42	118	72	1.64	1.1×10^{-8}	186	15	12.40	116	81	1.43	1.1×10^{-15}
ZW155	128	11	11.64	138	82	1.68	1.1×10^{-10}	188	20	9.40	164	78	2.10	3.2×10^{-9}
Pool	135	17	7.94	201	121	1.66	7.4×10^{-10}	252	32	7.88	240	114	2.11	2.2×10^{-10}
