# Supplementary material for: Adaptation of A-to-I RNA editing in Drosophila
Source: PLoS Genet. 2017 Mar 10;13(3):e1006648. doi: 10.1371/journal.pgen.1006648 (PMC5365144; doi:10.1371/journal.pgen.1006648)
Supplement: S25 Table — (PDF) [file pgen.1006648.s025.pdf]

Strain	B12	I17	N10	T07
I17	0.771			
N10	0.722	0.789		
T07	0.698	0.768	0.777	
ZW155	0.645	0.691	0.668	0.656
