# Supplementary material for: Adaptation of A-to-I RNA editing in Drosophila
Source: PLoS Genet. 2017 Mar 10;13(3):e1006648. doi: 10.1371/journal.pgen.1006648 (PMC5365144; doi:10.1371/journal.pgen.1006648)
Supplement: S26 Table — (PDF) [file pgen.1006648.s026.pdf]

Strain	B12	I17	N10	T07
I17	0.832			
N10	0.819	0.835		
T07	0.826	0.845	0.850	
ZW155	0.774	0.758	0.741	0.755
