# Supplementary material for: Adaptation of A-to-I RNA editing in Drosophila
Source: PLoS Genet. 2017 Mar 10;13(3):e1006648. doi: 10.1371/journal.pgen.1006648 (PMC5365144; doi:10.1371/journal.pgen.1006648)
Supplement: S29 Table — Fn is the number of strains of D. melanogaster that has N and S editing events detected in those strains [only editing events with P(E1) > 0.999 were counted]. (PDF) [file pgen.1006648.s029.pdf]

	non-PSEB					PSEB				
	F1	F2	F3	F4	Total	F1	F2	F3	F4	Total
Male: Level I										
N	73	17	3	1	94	10	2	5	0	17
S	27	17	2	1	47	6	0	0	0	6
Male: Level II										
N	54	9	2	1	66	7	1	0	0	8
S	22	13	2	0	37	4	0	0	0	4
Female: Level I										
N	54	12	3	2	71	10	4	0	0	14
S	40	6	1	2	49	3	0	0	0	3
Female:Level II										
N	44	9	2	0	55	5	2	0	0	7
S	32	4	1	1	38	2	0	0	0	2
