# Supplementary material for: Adaptation of A-to-I RNA editing in Drosophila
Source: PLoS Genet. 2017 Mar 10;13(3):e1006648. doi: 10.1371/journal.pgen.1006648 (PMC5365144; doi:10.1371/journal.pgen.1006648)
Supplement: S33 Table — Only the high-confidence sites with ≥ 10 raw reads in each library are used in the pairwise comparisons. (PDF) [file pgen.1006648.s033.pdf]

Library	B1	B2	B3	B4	B5	B6	B7
B2	0.900						
B3	0.894	0.887					
B4	0.850	0.850	0.906				
B5	0.933	0.905	0.888	0.859			
B6	0.915	0.900	0.905	0.867	0.921		
B7	0.883	0.870	0.918	0.903	0.887	0.902	
B8	0.855	0.849	0.897	0.918	0.865	0.880	0.922
