# Supplementary material for: Adaptation of A-to-I RNA editing in Drosophila
Source: PLoS Genet. 2017 Mar 10;13(3):e1006648. doi: 10.1371/journal.pgen.1006648 (PMC5365144; doi:10.1371/journal.pgen.1006648)
Supplement: S34 Table — (PDF) [file pgen.1006648.s034.pdf]

Up-regulated genes			
GO terms	Count	Percentage (%)	*P* value
translation	103	6.30	2.2×10^{-12}
ATP binding	160	9.79	2.0×10^{-5}
mitotic spindle elongation	30	1.83	1.1×10^{-5}
Golgi apparatus part	28	1.71	9.5×10^{-5}
mRNA metabolic process	53	3.24	3.6×10^{-4}
biopolymer glycosylation	19	1.16	7.4×10^{-4}
response to temperature stimulus	19	1.16	0.04
Down-regulated genes			
GO terms	Count	Percentage (%)	*P* value
oxidative phosphorylation	69	3.93	3.2×10^{-27}
mitochondrial ATP synthesis coupled electron transport	46	2.62	9.7×10^{-23}
