# Supplementary material for: Adaptation of A-to-I RNA editing in Drosophila
Source: PLoS Genet. 2017 Mar 10;13(3):e1006648. doi: 10.1371/journal.pgen.1006648 (PMC5365144; doi:10.1371/journal.pgen.1006648)
Supplement: S35 Table — (PDF) [file pgen.1006648.s035.pdf]

Up-regulated genes			
GO terms	Count	Percentage (%)	*P* value
ATP binding	192	9.97	3.7×10^{-8}
zinc ion binding	265	13.77	5.5×10^{-14}
regulation of nuclear mRNA splicing via spliceosome	27	1.40	8.9×10^{-6}
regulation of transcription	173	8.99	4.1×10^{-6}
Down-regulated genes			
Gene terms	Count	Percentage (%)	*P* value
oxidative phosphorylation	80	4.15	4.4×10^{-38}
