# Supplementary material for: Adaptation of A-to-I RNA editing in Drosophila
Source: PLoS Genet. 2017 Mar 10;13(3):e1006648. doi: 10.1371/journal.pgen.1006648 (PMC5365144; doi:10.1371/journal.pgen.1006648)
Supplement: S36 Table — (PDF) [file pgen.1006648.s036.pdf]

Primer ID	Oligo sequence
NaCP60E-F	CAGGAAGAGGAGATCGGCAT
NaCP60E-R	GCCGGAGAATGCTAGAGTGA
CaMKII-F	TCACCATTACAGGCTTGCAA
CaMKII-R	AGTTTGGCGTATGATTGGGC
nrm-F	AAGGTTTGAATAAGTCGCCTACC
nrm-R	CTTTAGAGCGTTAGAGTGGGC
Adar-F	TGCGTAGTATTTTGCGATTGG
Adar-R	TGGCATTCTTAAGCACGCAA
Adar-UTR-F	GCTGGCTGAAGAAACCCATT
Adar-UTR-R	GCAACGCATCGCATTCATTC
Rtp-F	CTGCCGCTTTATGAGACGAC
Rtp-R	GTGCGCCTAGTCCGAATTAG
DIP-F	TGTTTGCAGAGAGGTTCGAG
DIP-R	AGCGGGTTAATCGAAAGTGC
rdgA-F	CATTAACACGCTGAGAAATGCA
rdgA-R	CATGGCATCAAACCTACCCG
CG43967-F	CCCAGCACATTTTCGGCTAA
CG43967-R	GCACGGGATCTGCGTTAAAT
CG42540-1-F	GCTTTACGACCAGAGGATGC
CG42540-1-R	ATTTTGGTTCAACGACGGCC
CG42540-2-F	TCAGCTTATCGTTTGTGCCG
CG42540-2-R	AACTTTTGCCACAGACCGAC
CR18854-F	TGAAGATGGCCAAGGTACGT
CR18854-R	TTGTATGGACCAGGGTGAGC
roX1-F	GGCTTTACCGCTCTCTTTCG
roX1-R	TCCGAAGTAGCGAAAGTGGT
