# Supplementary material for: Adaptation of A-to-I RNA editing in Drosophila
Source: PLoS Genet. 2017 Mar 10;13(3):e1006648. doi: 10.1371/journal.pgen.1006648 (PMC5365144; doi:10.1371/journal.pgen.1006648)
Supplement: S37 Table — (PDF) [file pgen.1006648.s037.pdf]

Primer ID	Oligo sequence
rp49-qF	GCGCTGTTCGCAAGTGT
rp49-qR	TGGGCAGTATCCATTGAGTTT
adar-qF	GCCTCAGATACACGCGGATA
adar-qR	TGCCCGCTAATACCTTTCGA
