# Supplementary figures and images for: Adaptation of A-to-I RNA editing in Drosophila
Source: PLoS Genet. 2017 Mar 10;13(3):e1006648. doi: 10.1371/journal.pgen.1006648 (PMC5365144; doi:10.1371/journal.pgen.1006648)

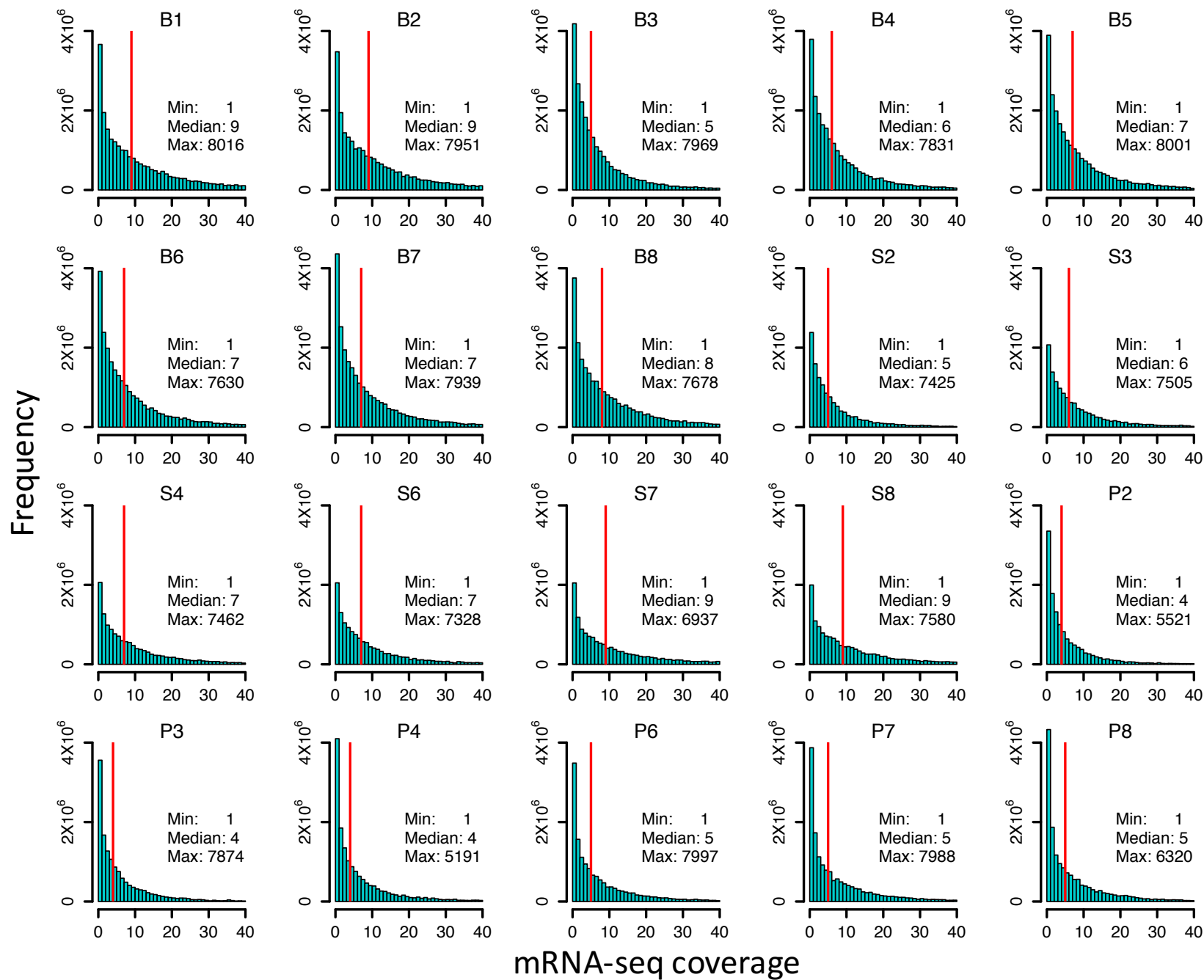

Supplement: S1 Fig — (PDF) [file pgen.1006648.s038.pdf]

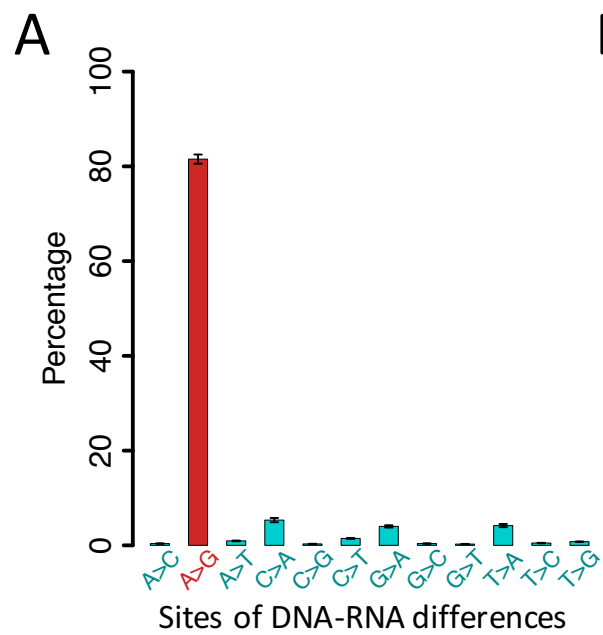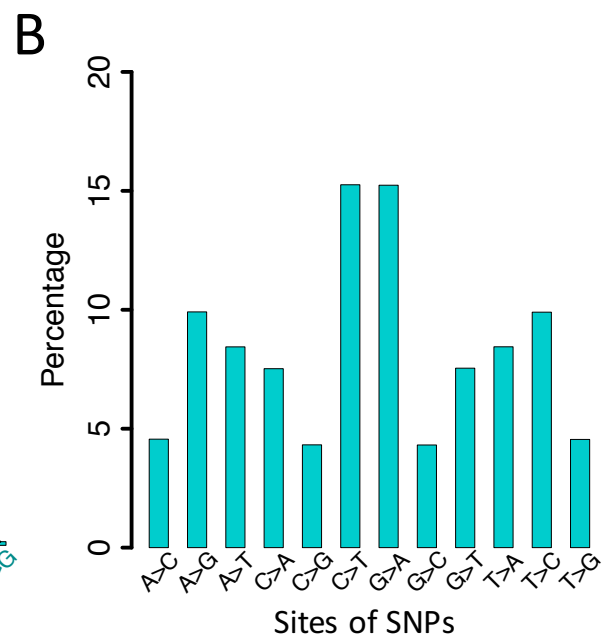

Supplement: S2 Fig — (A) Percentages of sites with different types of DNA-RNA differences detected in the female and male brains of D. melanogaster across eight libraries (the error bars represent the s.e. across eight libraries). (B) Percentages of the sites of different types of SNPs (reference>alternative allele) from the global populations of D. melanogaster. (PDF) [file pgen.1006648.s039.pdf]

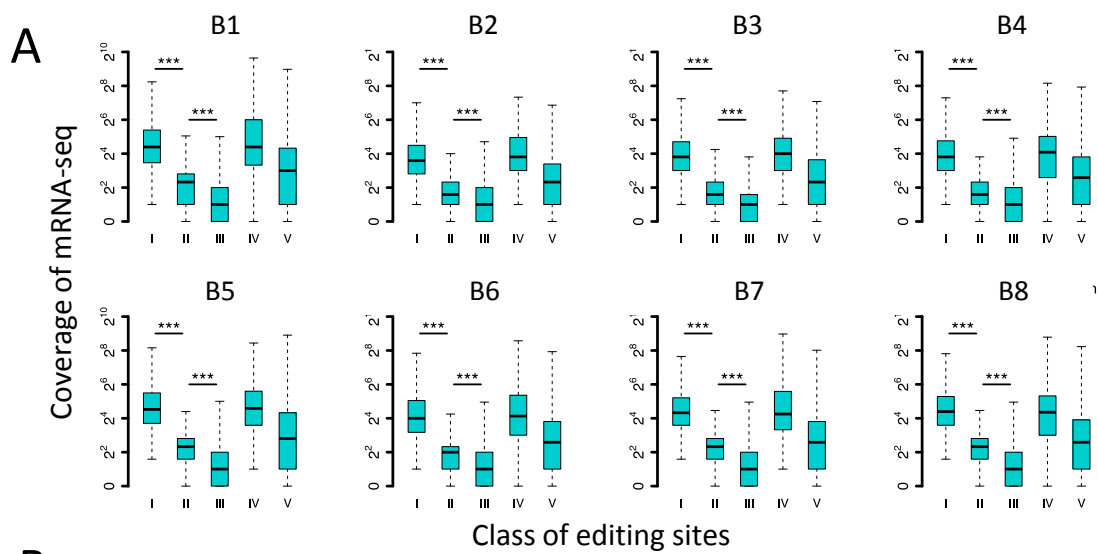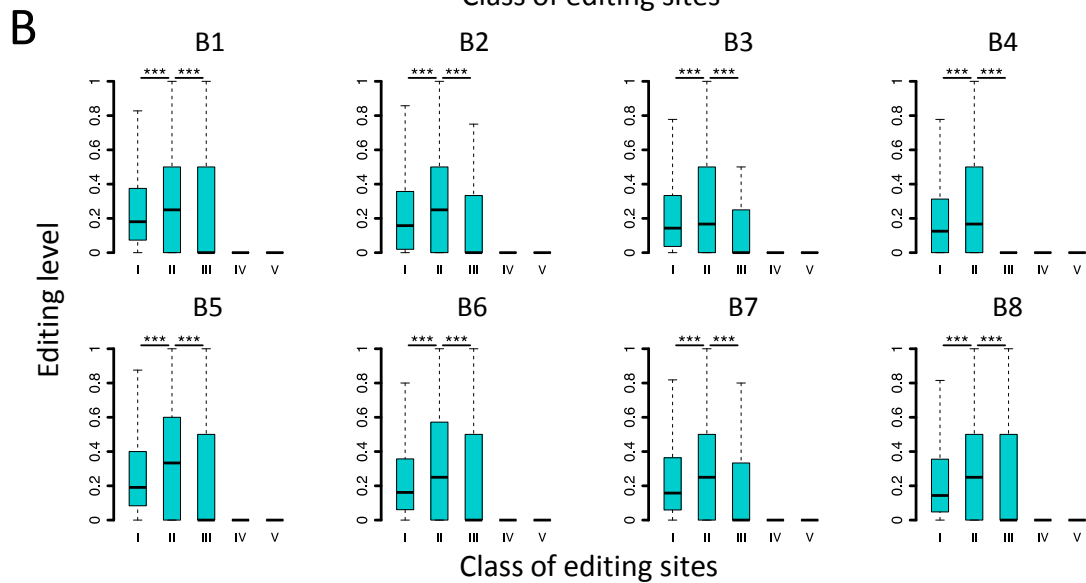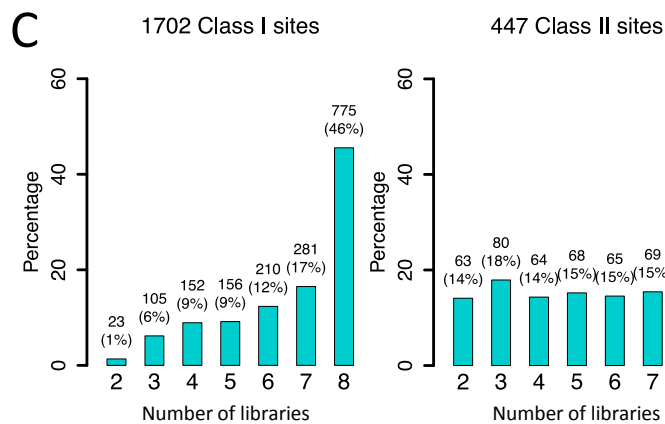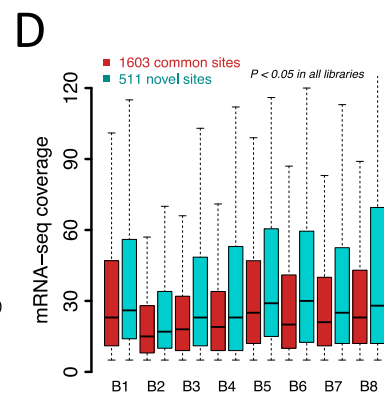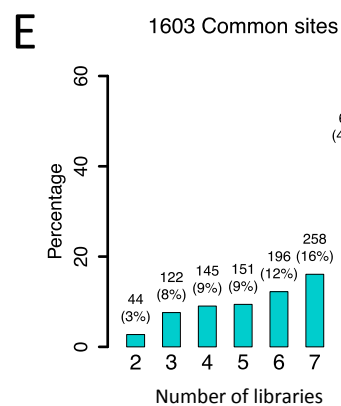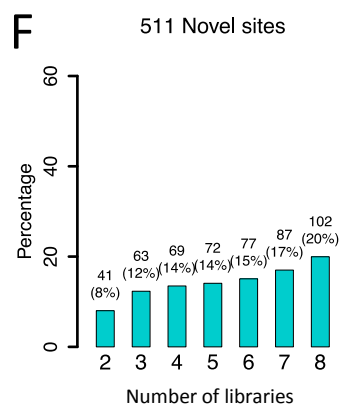

Supplement: S3 Fig — (A) Sequencing coverage (y-axis, at log2 scale) of five classes of editing sites (***, P < 0.001). (B) Editing level of five classes of editing sites (***, P < 0.001). (C) The percentage of Class I and Class II editing sites with respect to the number of brain libraries in which editing events are detected (x-axis). The number and percentage of editing sites are given above the bars. (D) The sequencing coverage of common and novel editing sites in each brain library (P < 0.05 in each of the eight libraries, KS tests). (E) The percentage of common sites with respect to the number of brain libraries in which editing events are detected (x-axis). The number and percentage of editing sites are given above the bars. (F) The percentage of novel sites with respect to the number of brain libraries in which editing events are detected (x-axis). The number and percentage of editing sites are given above the bars. (PDF) [file pgen.1006648.s040.pdf]

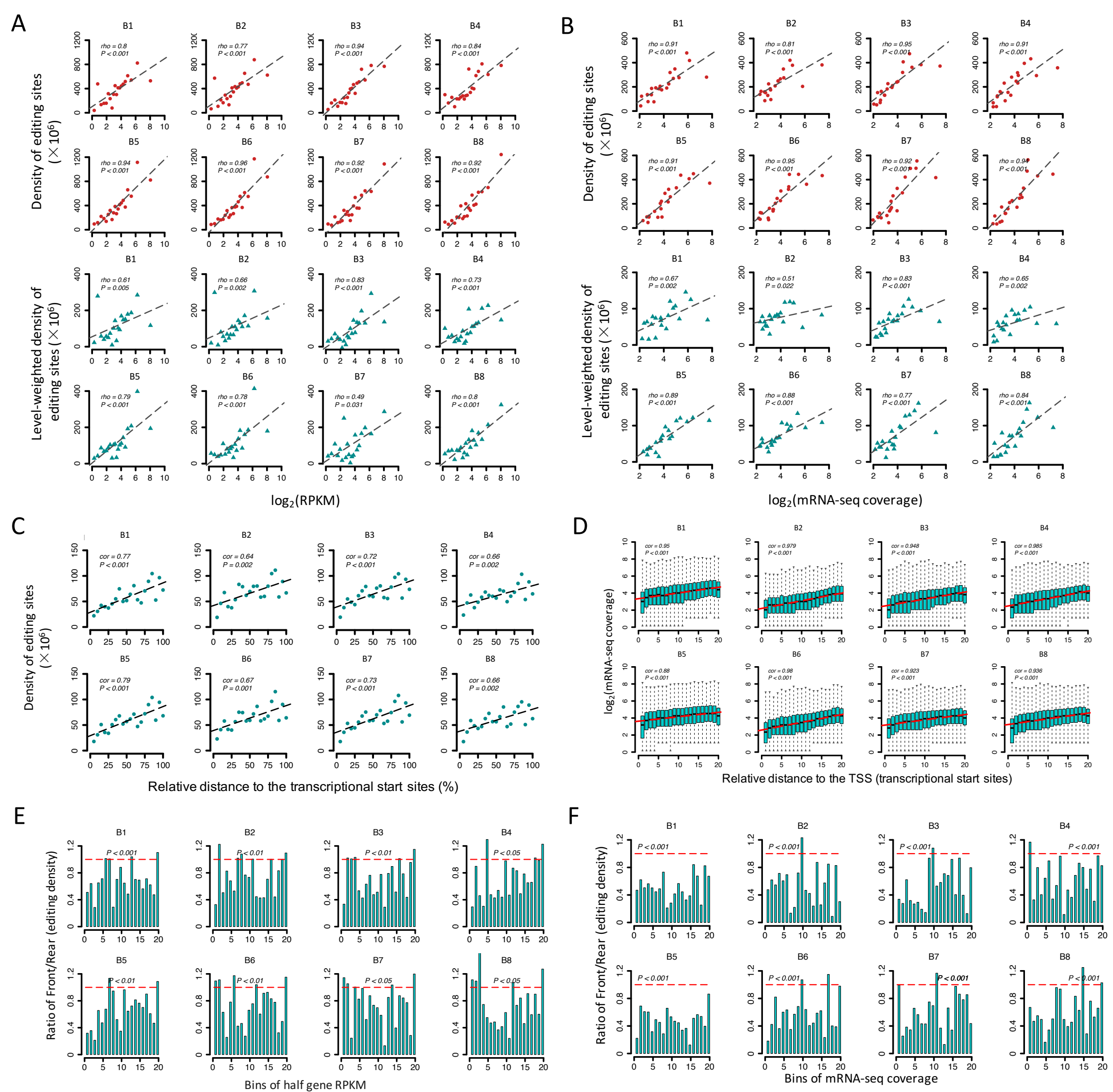

Supplement: S4 Fig — (A) The density of editing sites is significantly positively correlated with expression level of host genes (RPKM) in each brain library. The genes expressed in brains were ranked with increasing RPKM and divided into 20 bins (the x-axis). In each bin, the density of editing sites (or level-weighted density) (y-axis) is calculated by dividing the observed number of editing sites (or weighting each site with its editing level) with the total number of adenosine sites (per million). There is a significantly positive correlation between the density of editing sites (or level-weighted density) and the gene expression level (P < 0.05 in each library). (B) The density of editing sites is significantly positively correlated with mRNA-seq coverage in each brain library. All the expressed adenosine sites (≥ 5X coverage) are ranked with increasing sequencing coverage and binned into 20 categories (x-axis). There is a significantly positive correlation between the density of editing sites (or level-weighted density) and the mRNA-seq coverage (P < 0.05 in each library). (C) The density of editing sites is significantly positively correlated with the relative distance to the transcriptional start site in each brain library. After dividing all the adenosine sites (≥ 5X coverage) into 20 equal bins along their positions in pre-mRNAs (x-axis), the editing density in each bin is significantly positively correlated with the relative distance to the transcriptional start sites (P < 0.005 in each library). (D) mRNA-Seq coverage slightly increases towards 3' ends of mRNAs. In each brain library, after dividing the adenosine sites (≥ 5X coverage) into 20 equal bins along their positions in pre-mRNAs (x-axis), the median value of mRNA-Seq coverage (y-axis) increases along the relative position of that bin (P < 0.01 in each library). (E) The density of editing is significantly higher in the rear half-gene compared to front half-gene of pre-mRNAs. Each gene is split into two equal parts (at [file pgen.1006648.s041.pdf]

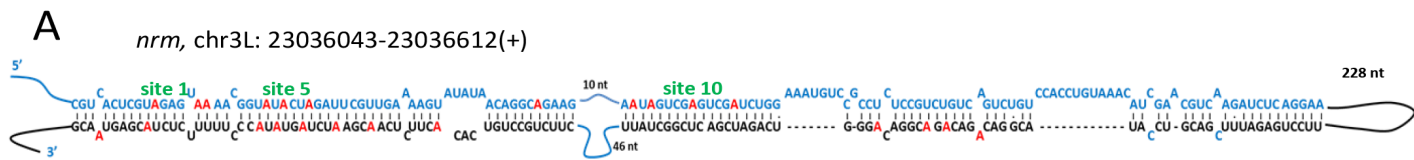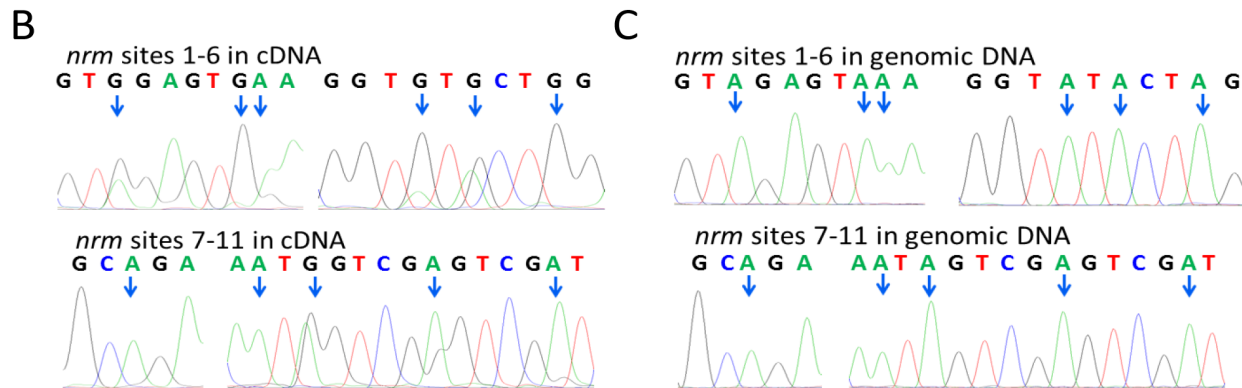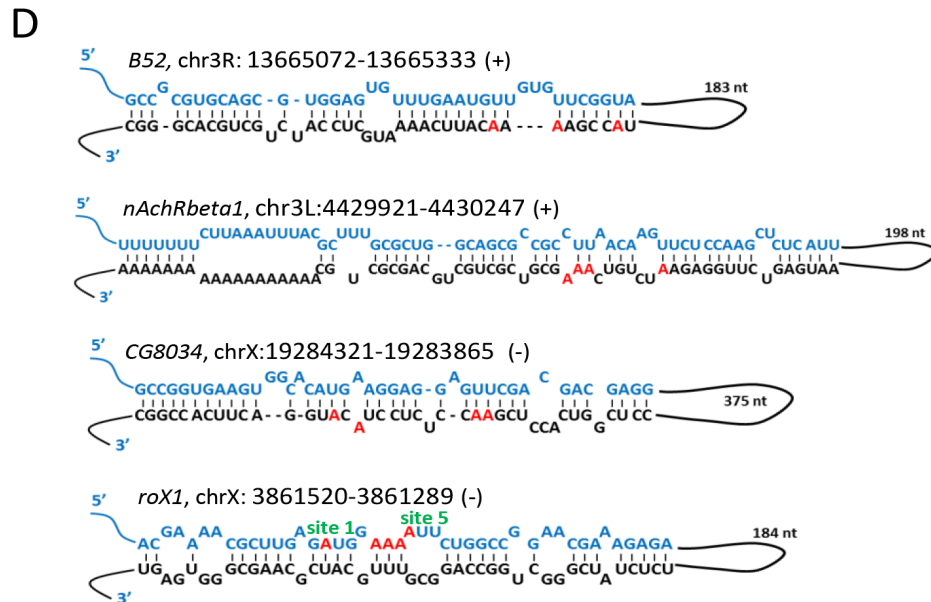

Supplement: S6 Fig — (A) Abundant A-to-I editing sites are located in the stems of a long-range pseudoknot formed by an intron of nrm in D. melanogaster. Verification of the editing events in introns of nrm by Sanger sequencing the cDNA from female brains (B) and genomic DNA of D. melanogaster (C). (D) Editing events located in the stems of pre-mRNA long-range pseudoknots of B52, nAchRbeta1, CG8034 and roX1. (PDF) [file pgen.1006648.s043.pdf]

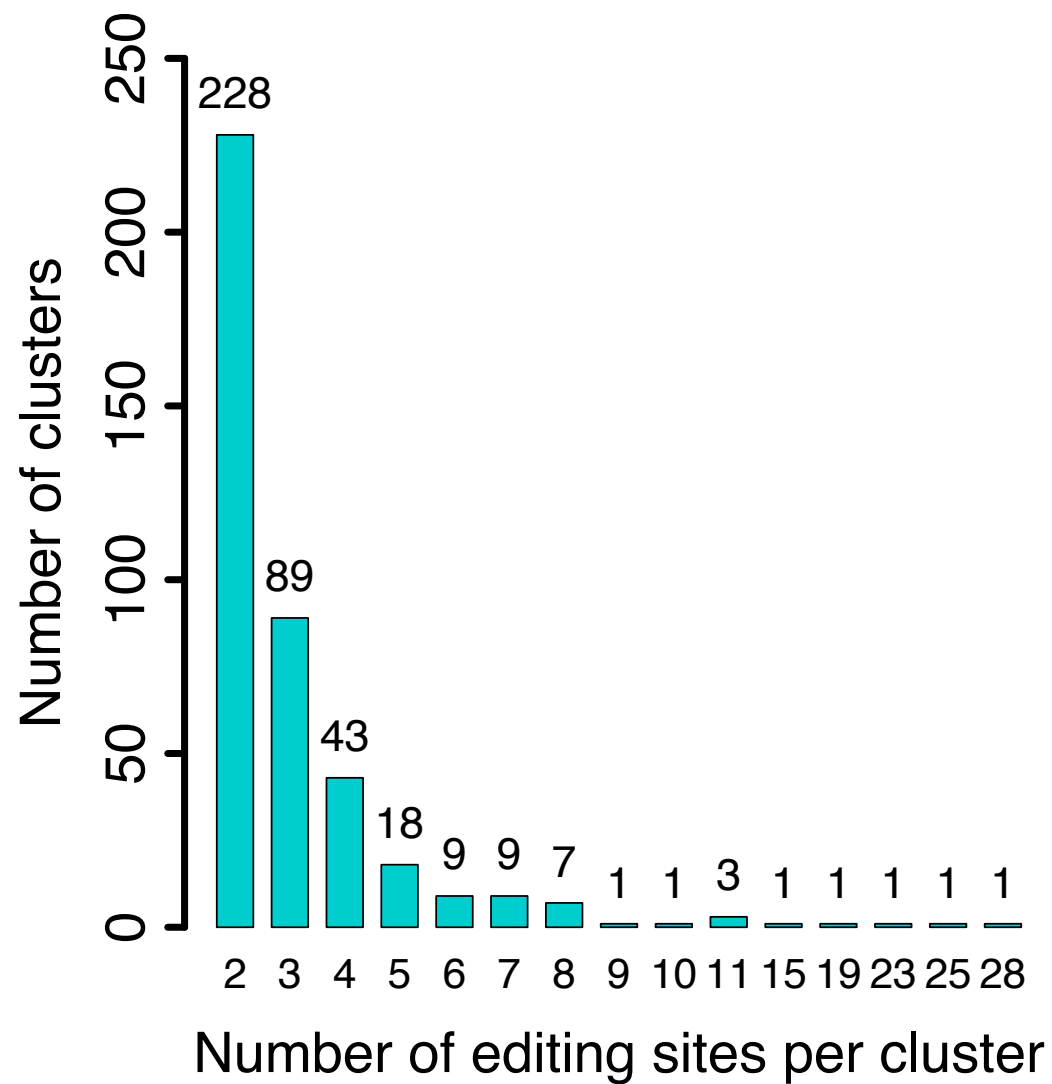

Supplement: S7 Fig — By clustering the editing events with distances smaller than 100 nucleotides (nts), we identified a total of 1,320 editing events that form 413 clusters. The y-axis is the number of clusters with different number of editing sites. (PDF) [file pgen.1006648.s044.pdf]

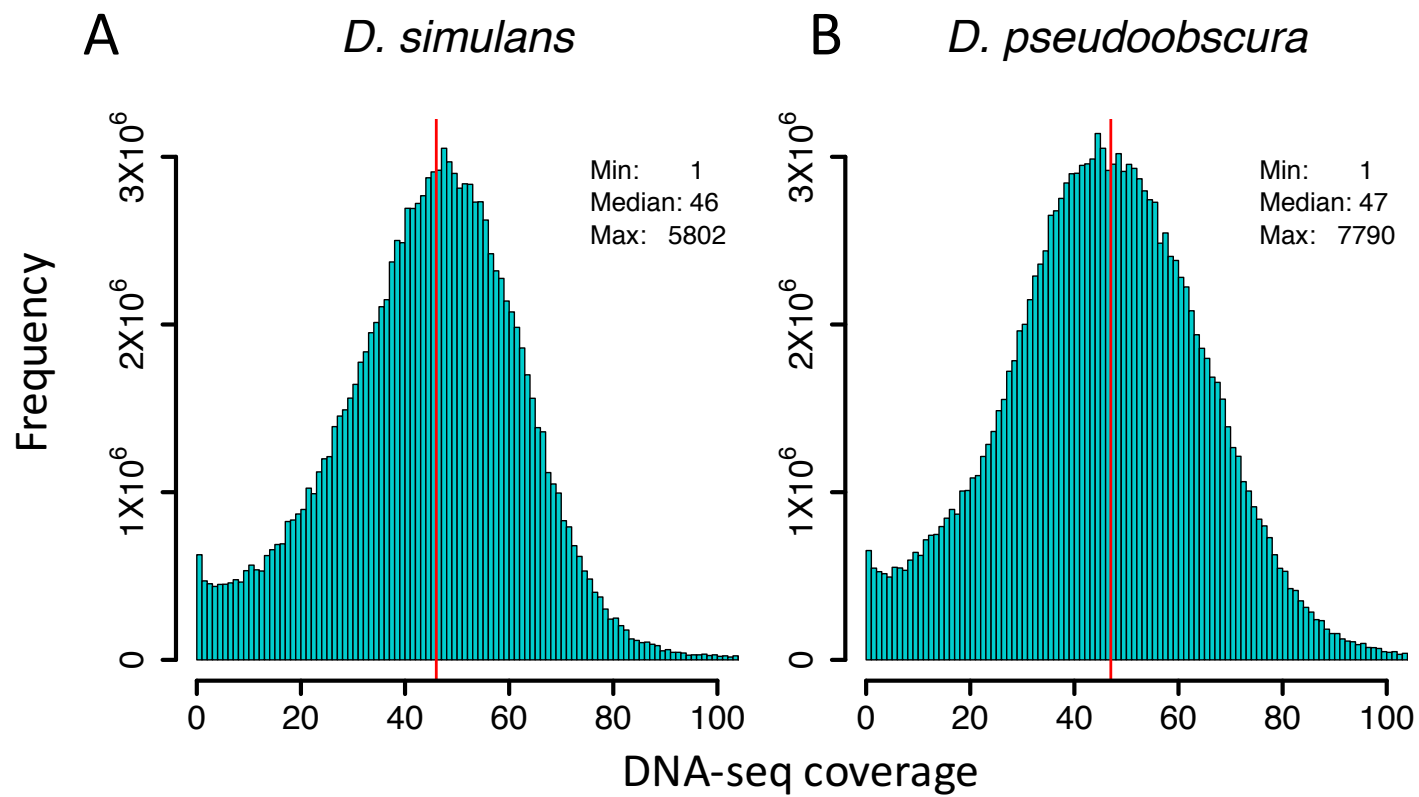

Supplement: S9 Fig — Distribution of DNA-seq coverage (x-axis) of D. simulans (A) and D. pseudoobscura (B) used in this study. (PDF) [file pgen.1006648.s046.pdf]

■ Conserved  
■ Non-conserved

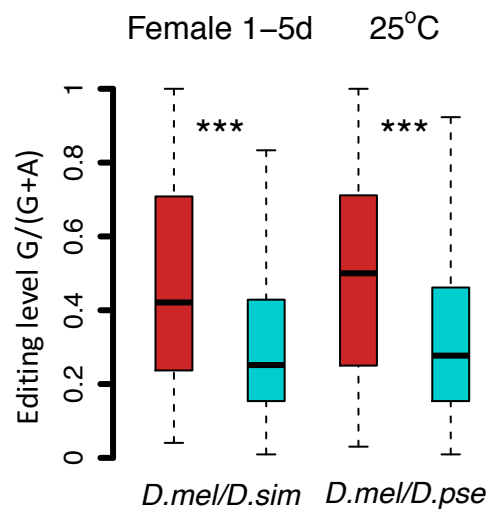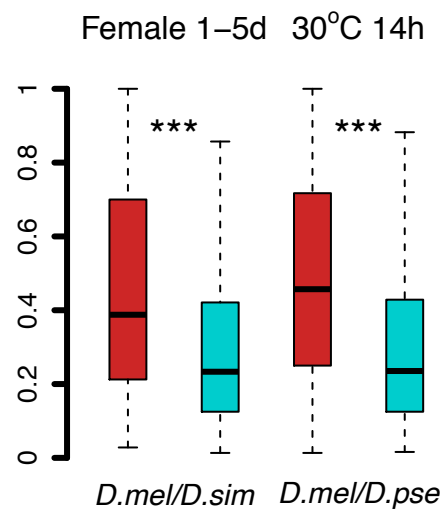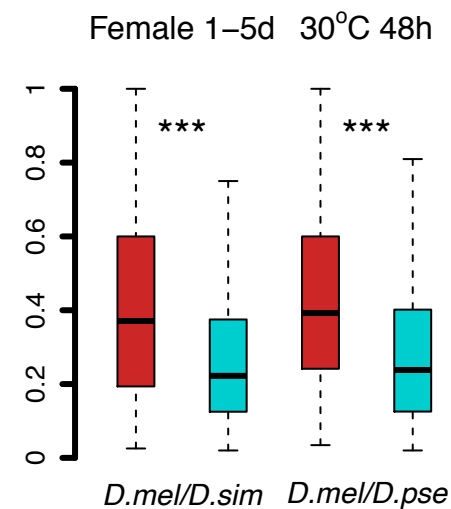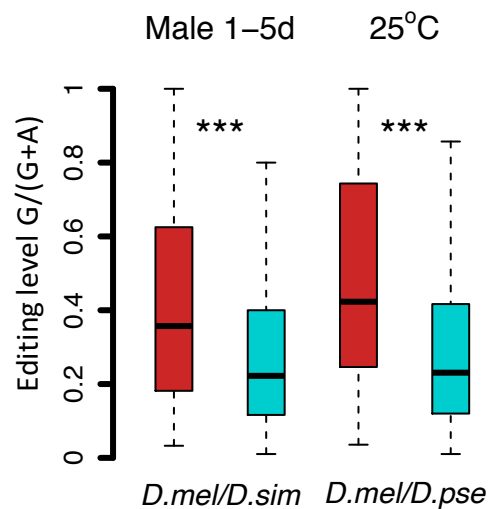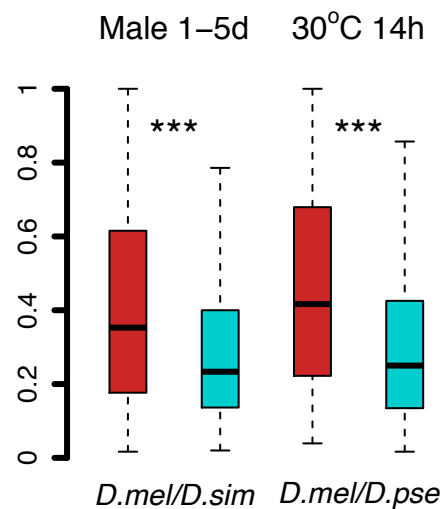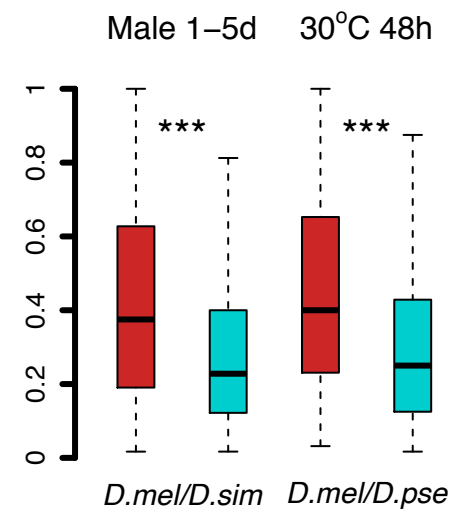

Supplement: S10 Fig — *, P < 0.05; * *, P< 0.01; * * *, P < 0.001, KS test. (PDF) [file pgen.1006648.s047.pdf]

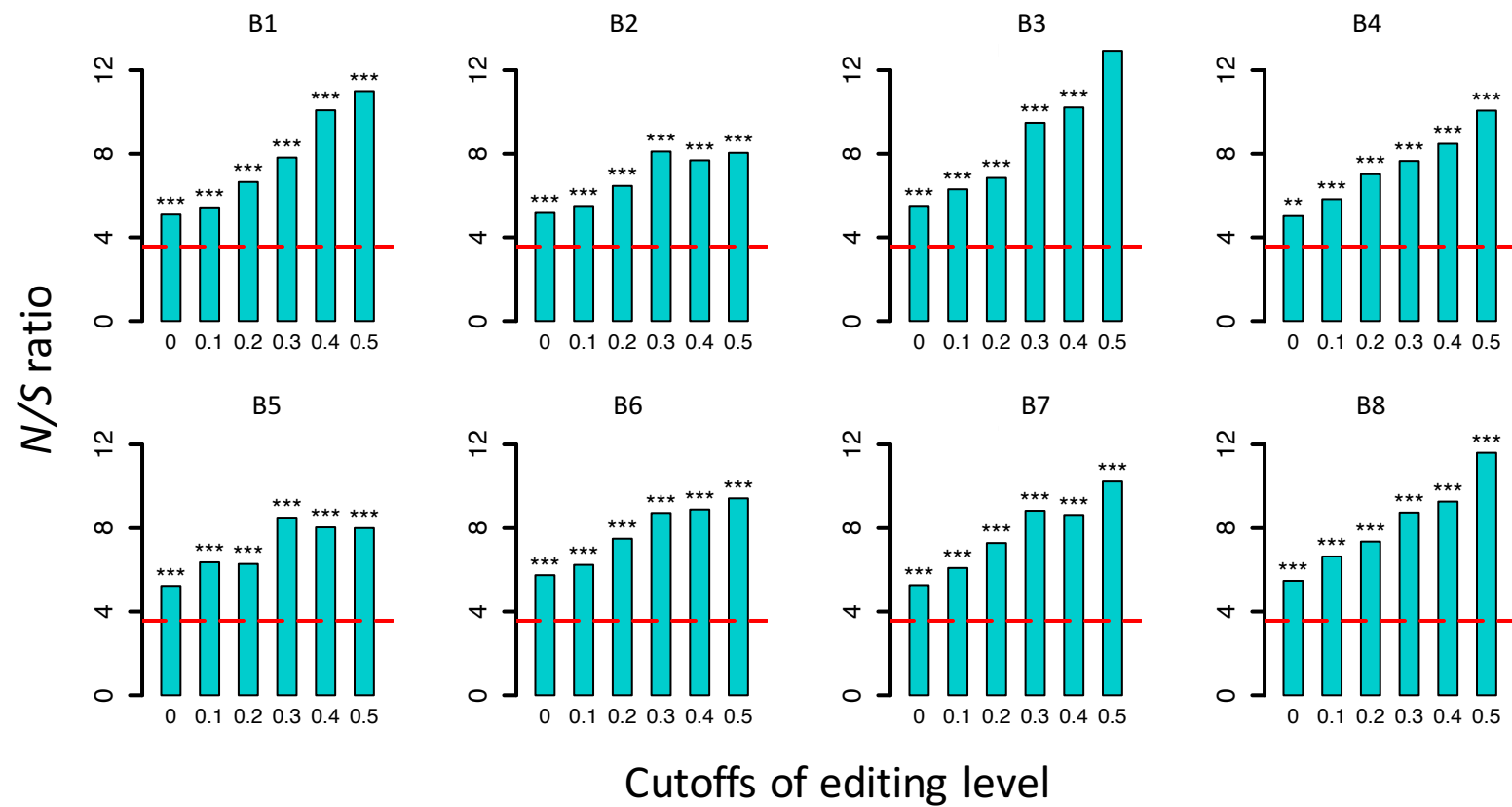

S11 Fig

Supplement: S11 Fig — The editing level cutoffs used are 0, 0.1, 0.2, 0.3, 0.4 and 0.5. The N/S ratio under neutral evolution (3.80) is indicated with red dashed lines. (* * *, P < 0.001, Fisher’s exact tests). (PDF) [file pgen.1006648.s048.pdf]

Editing level G/(G+A)

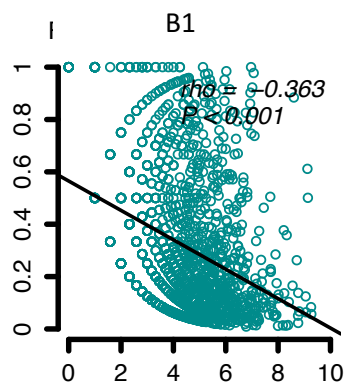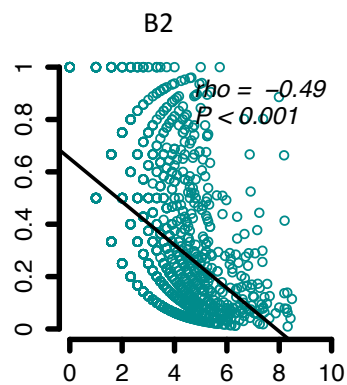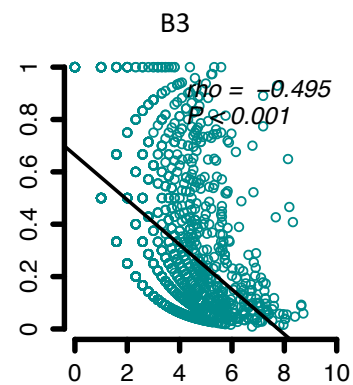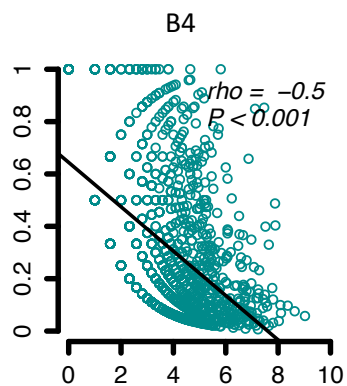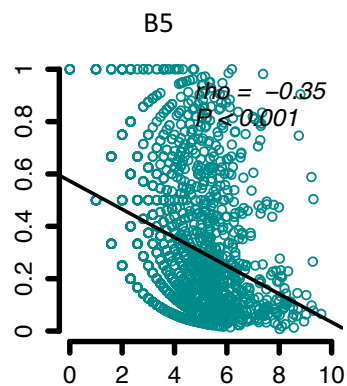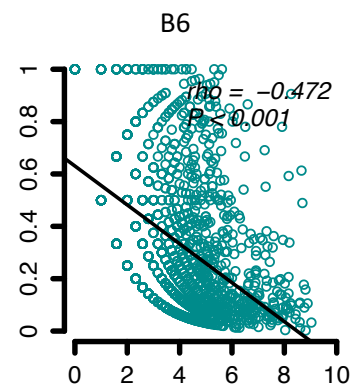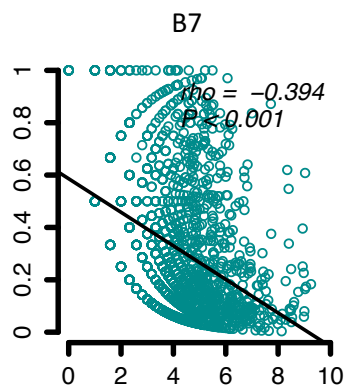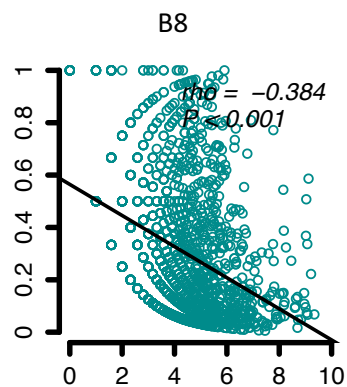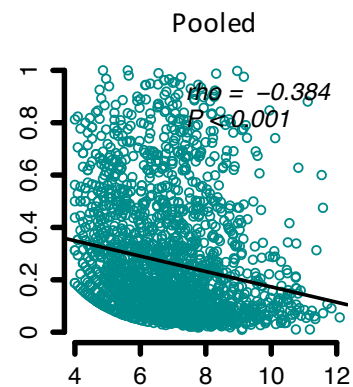

$\log_2(\text{mRNA-seq coverage})$

Supplement: S12 Fig — (PDF) [file pgen.1006648.s049.pdf]

— Observed  $N/S$  ratio    — Median of simulated  $N/S$  ratio    ■ 2.5% - 97.5% quantile    - - - Neutral expectation (3.80)

B1      B2      B3      B4      B5      B6      B7      B8

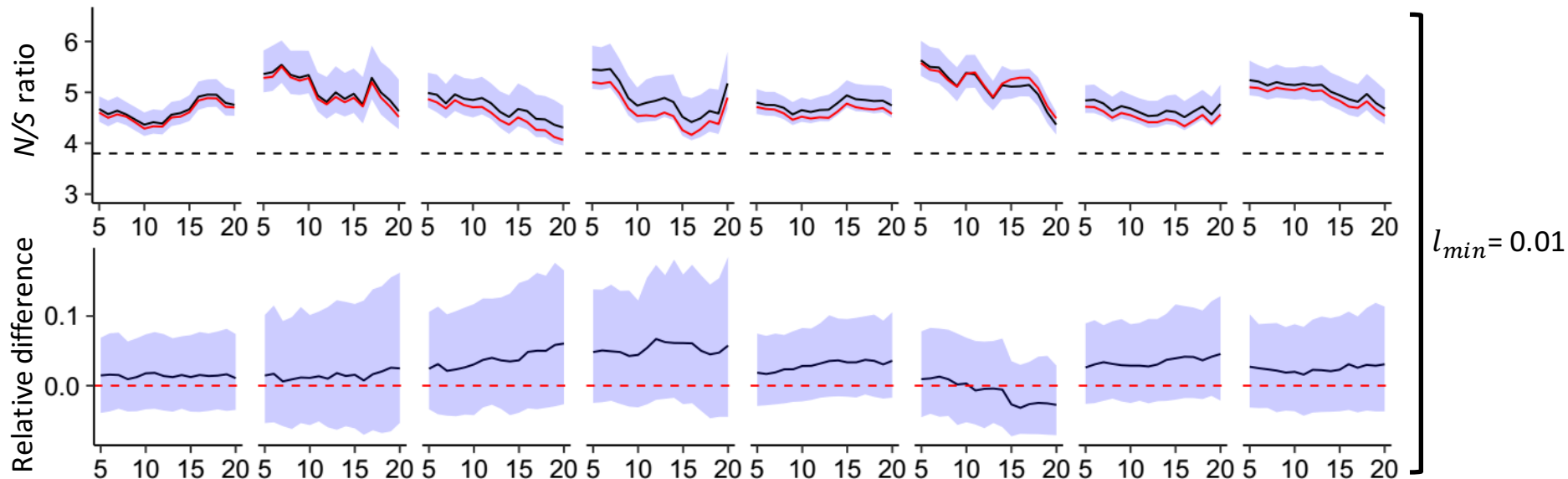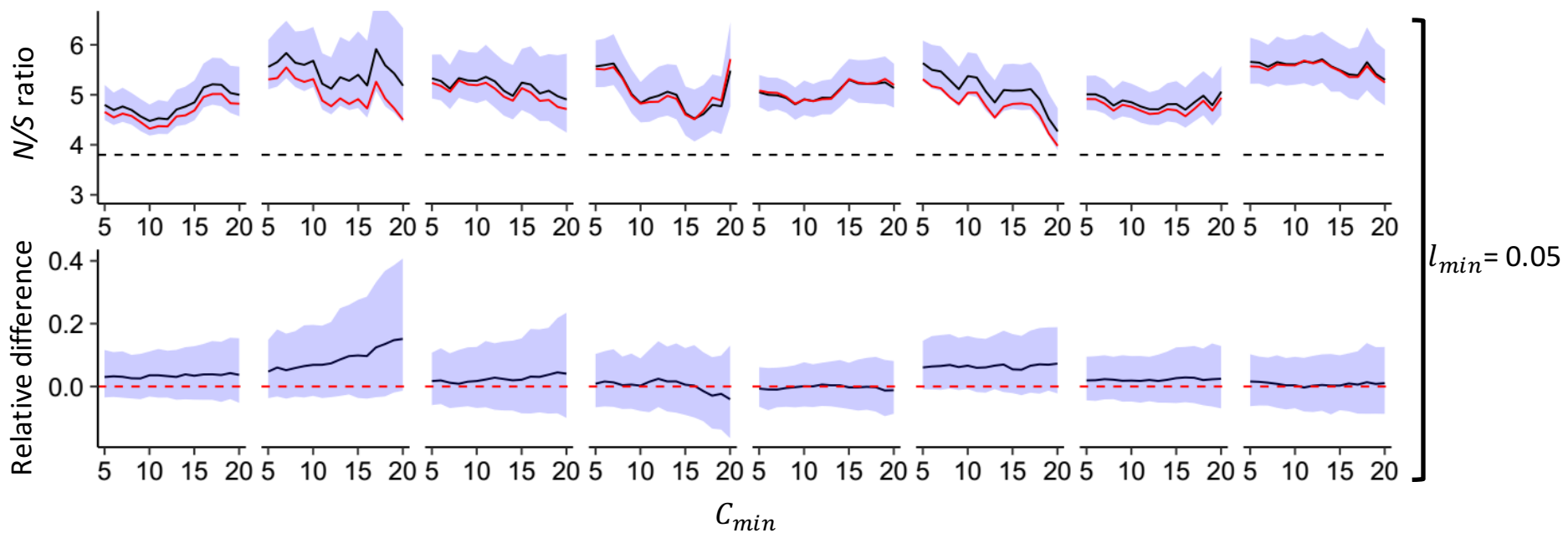

Supplement: S13 Fig — The x-axis is the cutoff of coverage (Cmin) and the y-axis is the simulated (median in black, and the range from 2.5% to 97.5% quantile is in blue) and observed (red) N/S ratio. The N/S ratio under neutral evolution (3.80) is indicated with dashed lines. The cutoff of editing level, lmin = 0.01 at top and 0.05 at bottom. (PDF) [file pgen.1006648.s050.pdf]

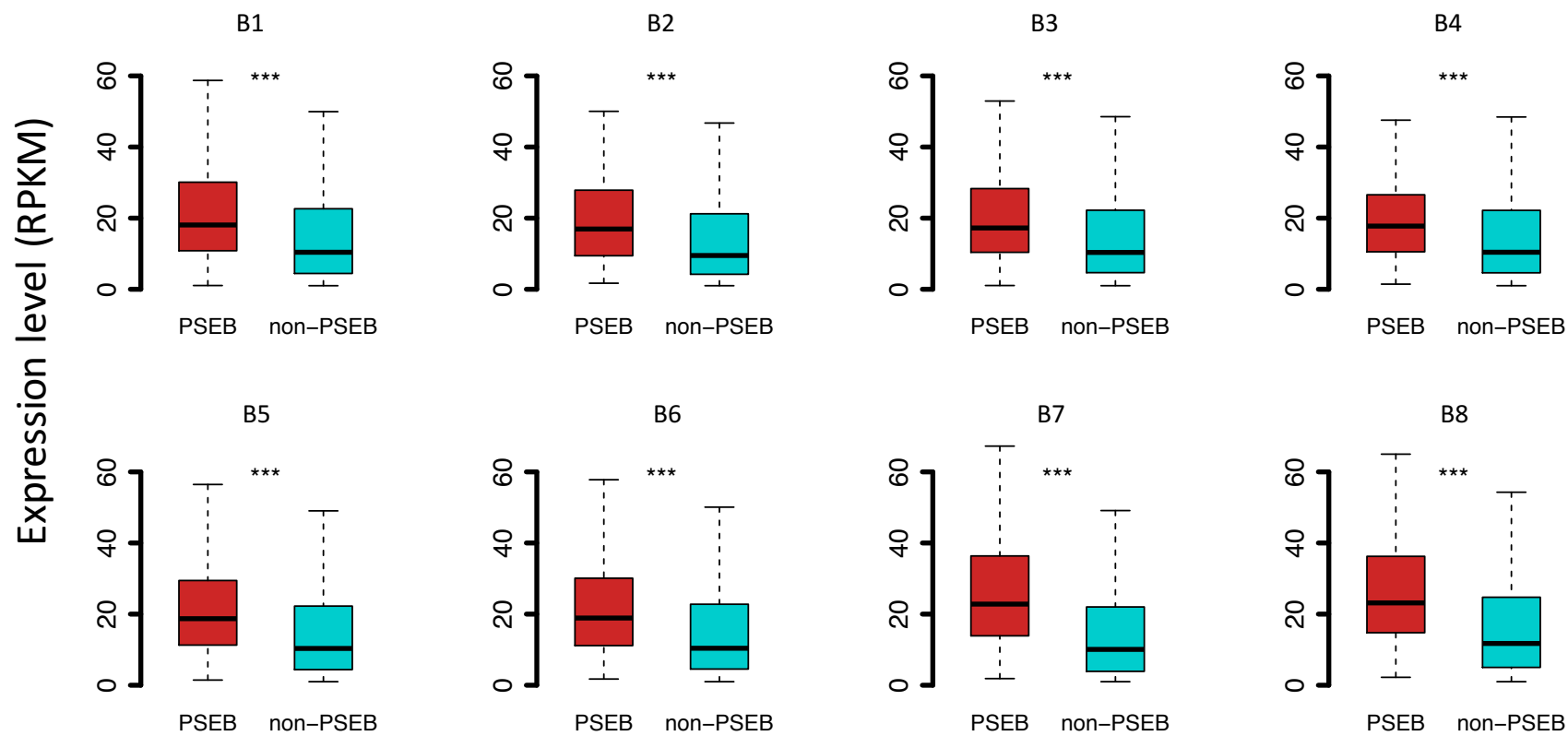

Supplement: S16 Fig — (PDF) [file pgen.1006648.s053.pdf]

Editing level G/(A+G)

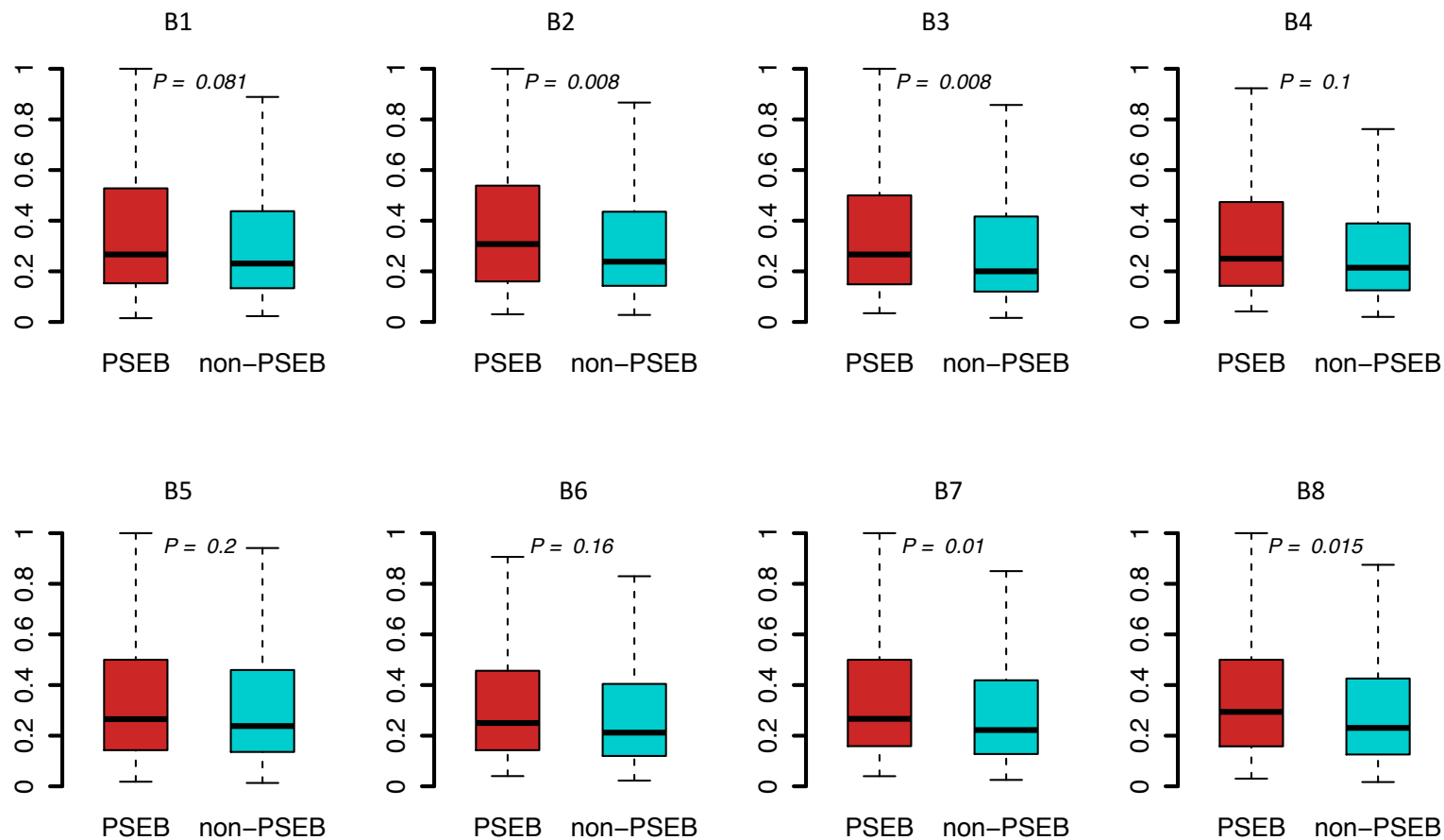

Supplement: S17 Fig — (PDF) [file pgen.1006648.s054.pdf]

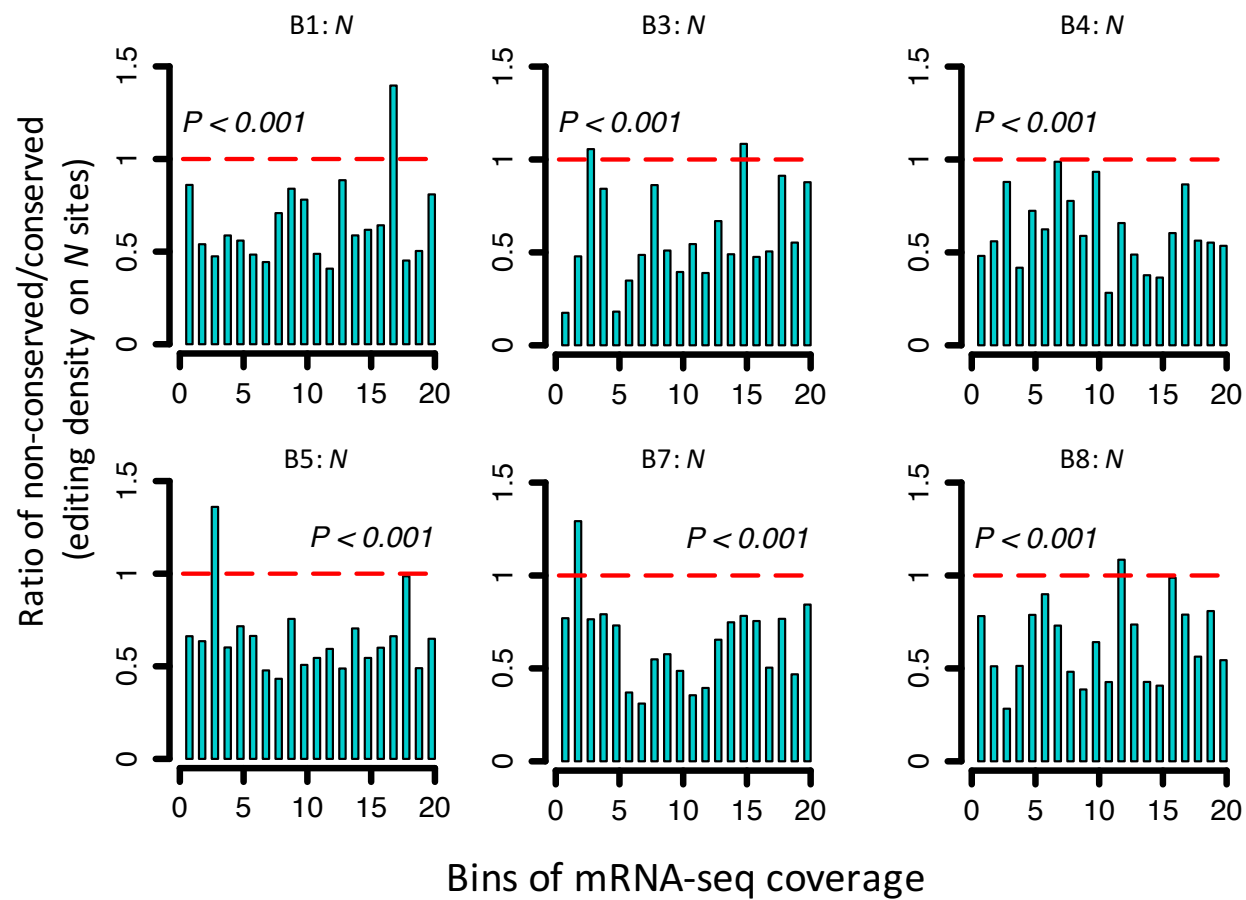

Supplement: S18 Fig — All the nonsynonymous adenosine sites (cause amino acid changes if edited; ≥ 5X coverage) are ranked with increasing sequencing coverage and binned into 20 categories (x-axis). Within each bin, the sites are divided into two equal-sized subgroups based on the phyloP scores. The y-axis is the editing density of the non-conserved relative to the conserved subgroup in each bin (P < 0.001 in each comparison, paired t test). (PDF) [file pgen.1006648.s055.pdf]

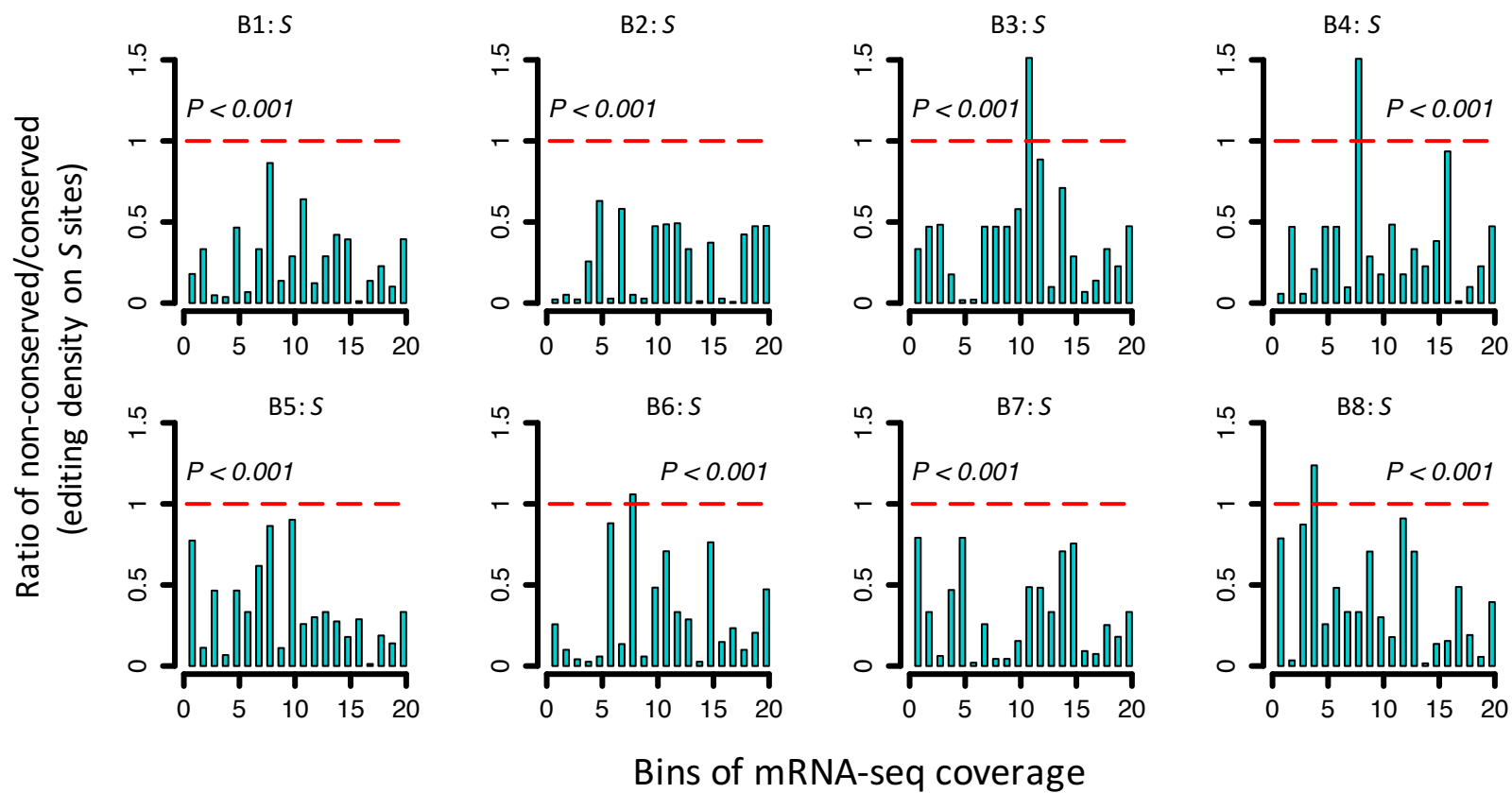

Supplement: S19 Fig — All the synonymous adenosine sites (do not cause amino acid changes if edited; ≥ 5X coverage) are ranked with increasing sequencing coverage and binned into 20 categories (x-axis). Within each bin, the sites are divided into two equal-sized subgroups based on the phyloP scores. The y-axis is the editing density of the non-conserved relative to the conserved subgroup in each bin (P < 0.001 in each comparison, paired t test). (PDF) [file pgen.1006648.s056.pdf]

Frequency

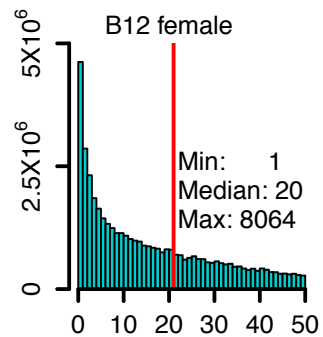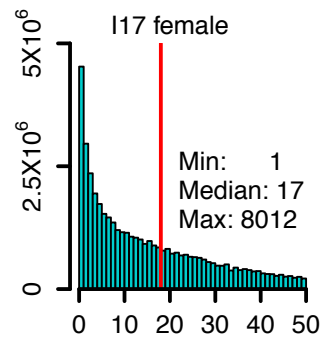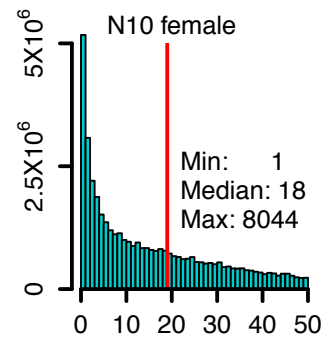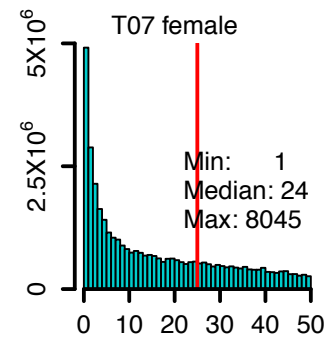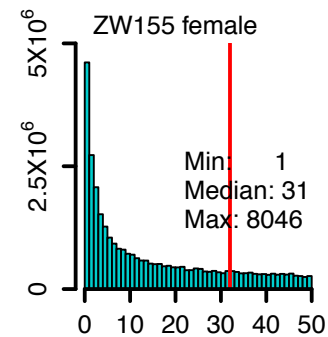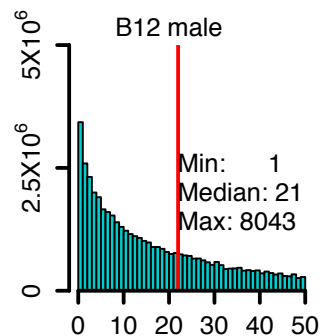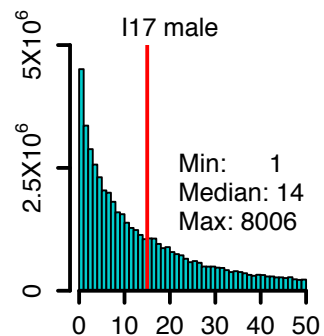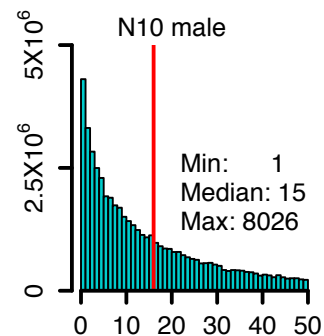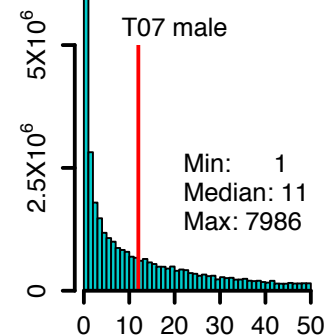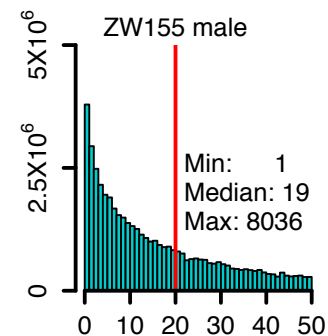

mRNA-seq coverage

Supplement: S20 Fig — (PDF) [file pgen.1006648.s057.pdf]

— Observed  $N/S$  ratio    — Median of simulated  $N/S$  ratio    ■ 2.5% - 97.5% quantile    - - - Neutral expectation (3.80)

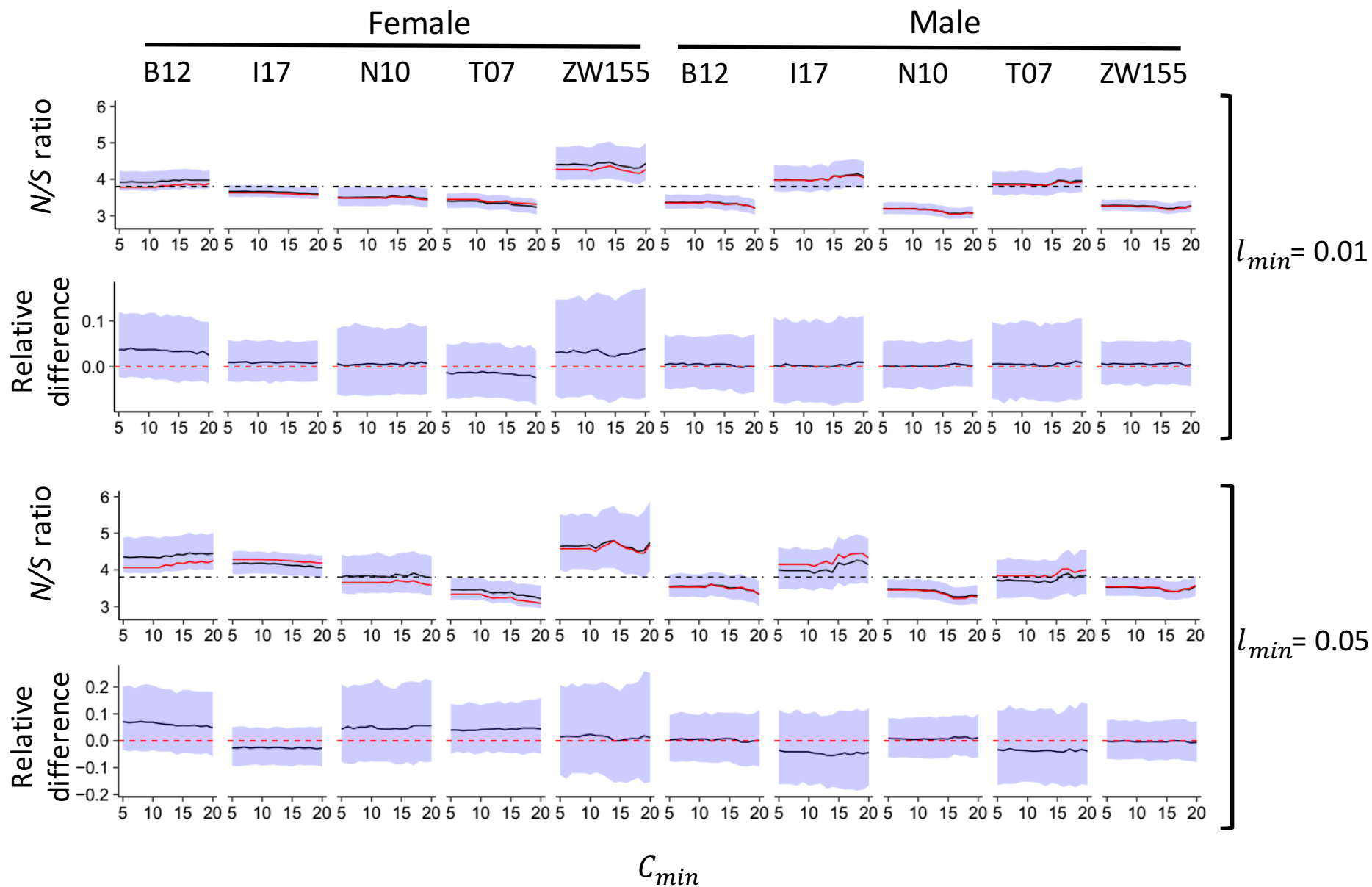

Supplement: S21 Fig — The x-axis is the cutoff of coverage (Cmin) and the y-axis is the simulated (median in black, and the range from 2.5% to 97.5% quantile is in blue) and observed (red) N/S ratio. The N/S ratio under neutral evolution (3.80) is indicated with dashed lines. The corresponding relative differences (the simulated/observed N/S ratio) for each simulation is given at the bottom panel. The editing level cutoffs lmin = 0.01 and 0.05 are used in the simulations. (PDF) [file pgen.1006648.s058.pdf]

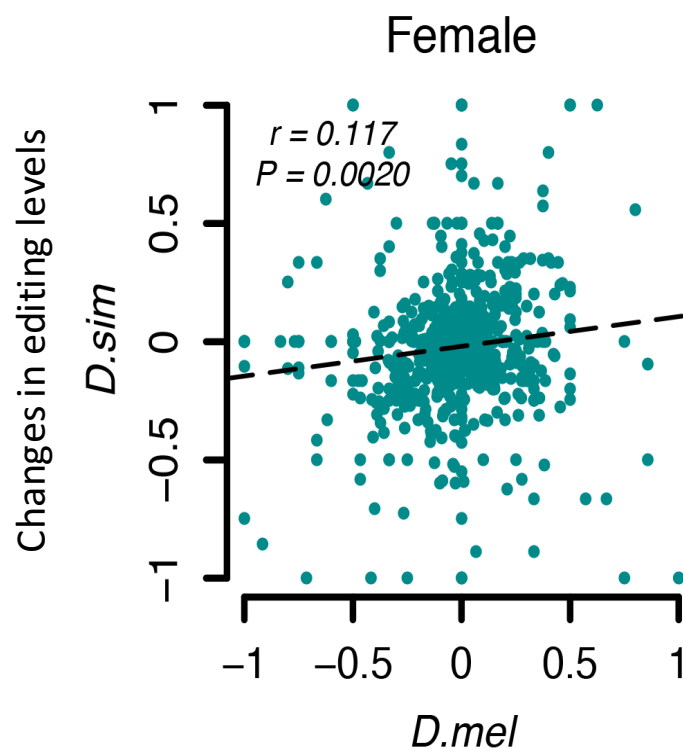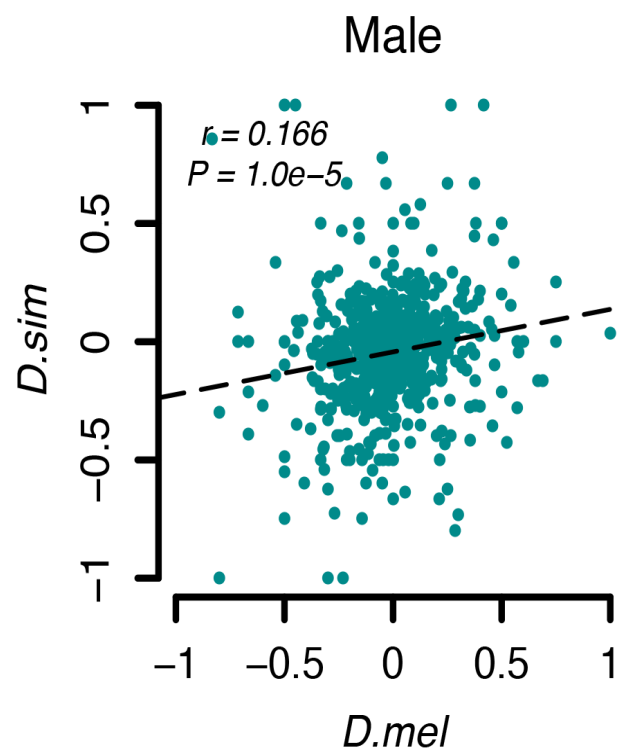

Changes in editing levels

Supplement: S22 Fig — (PDF) [file pgen.1006648.s059.pdf]

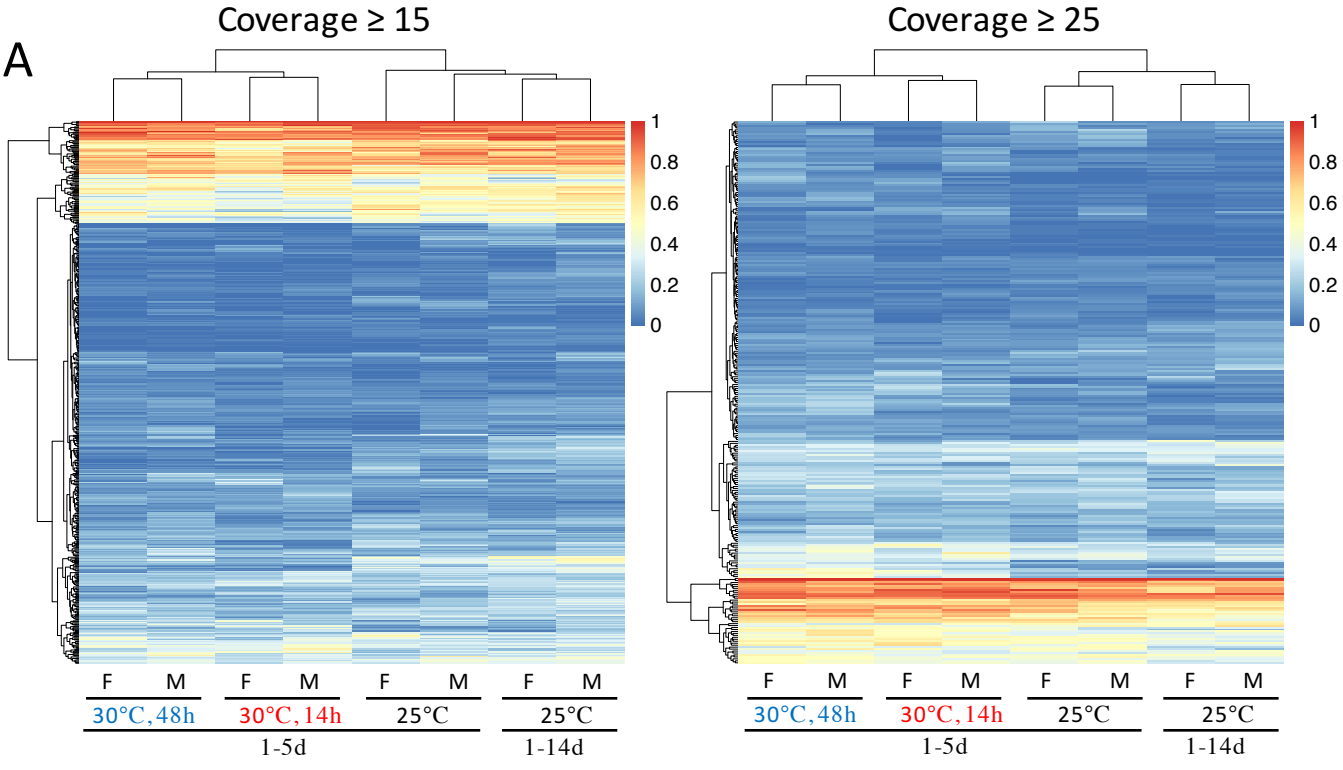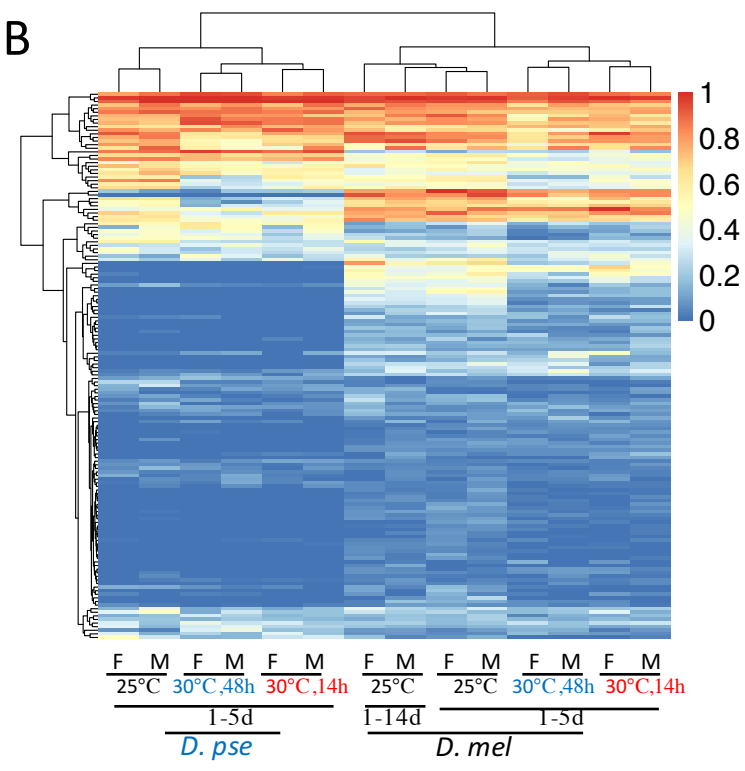

Supplement: S23 Fig — (A). Clustering the brain libraries of D. melanogaster based on the editing levels of high-confidence editing sites that have at least 15 (left) or 25 (right) raw reads in each brain library. Note flies of the same accommodation conditions always cluster together. (B). Clustering the brain libraries of D. melanogaster and D. pseudoobscura based on the editing levels of 152 high-confidence editing sites that have at least 20 raw reads in each brain library. Note species divergence plays a more important role than temperature in clustering the samples. (PDF) [file pgen.1006648.s060.pdf]

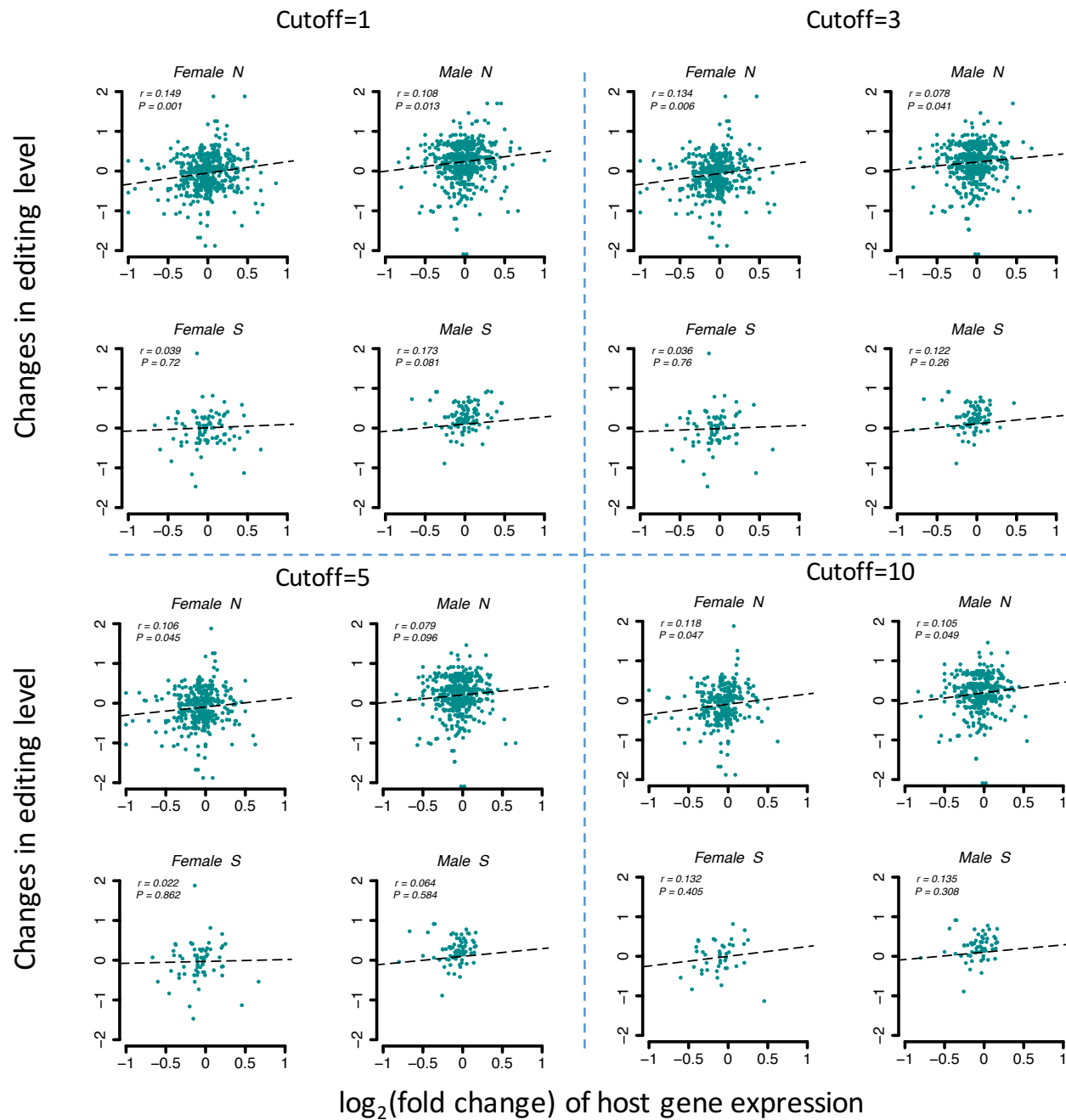

Supplement: S24 Fig — In contrast, no significant patterns are observed for the S sites (RPKM cutoff of 1, 3, 5, or 10 was used). (PDF) [file pgen.1006648.s061.pdf]

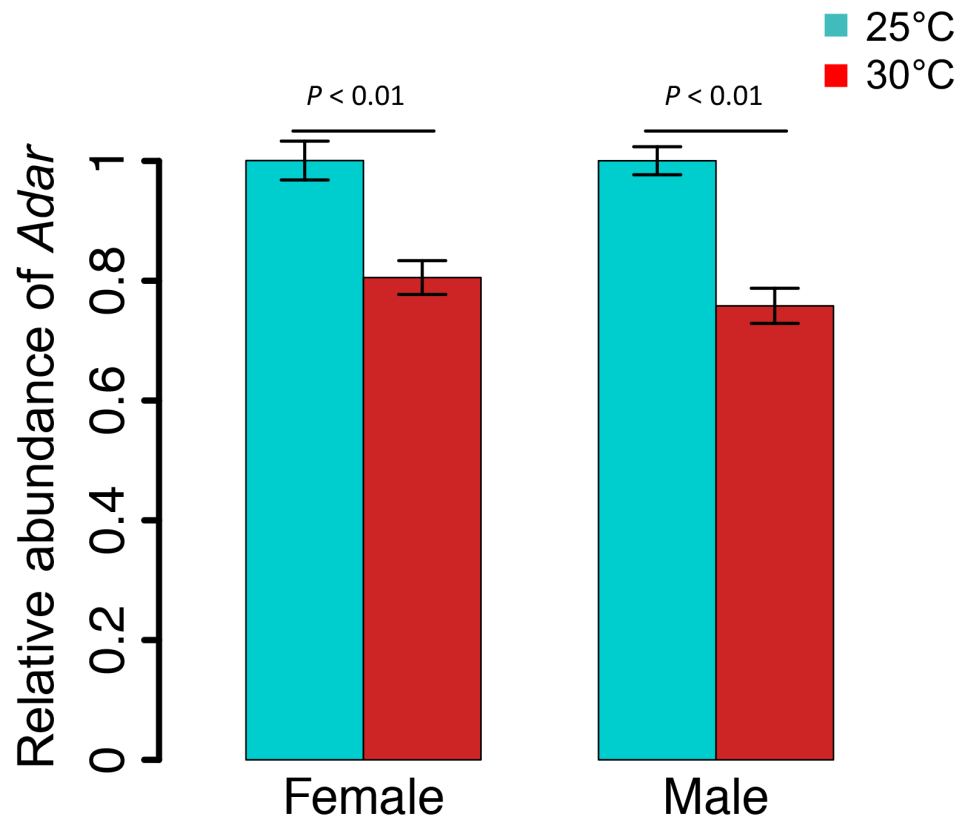

Supplement: S25 Fig — The error bars are s.e.. (PDF) [file pgen.1006648.s062.pdf]

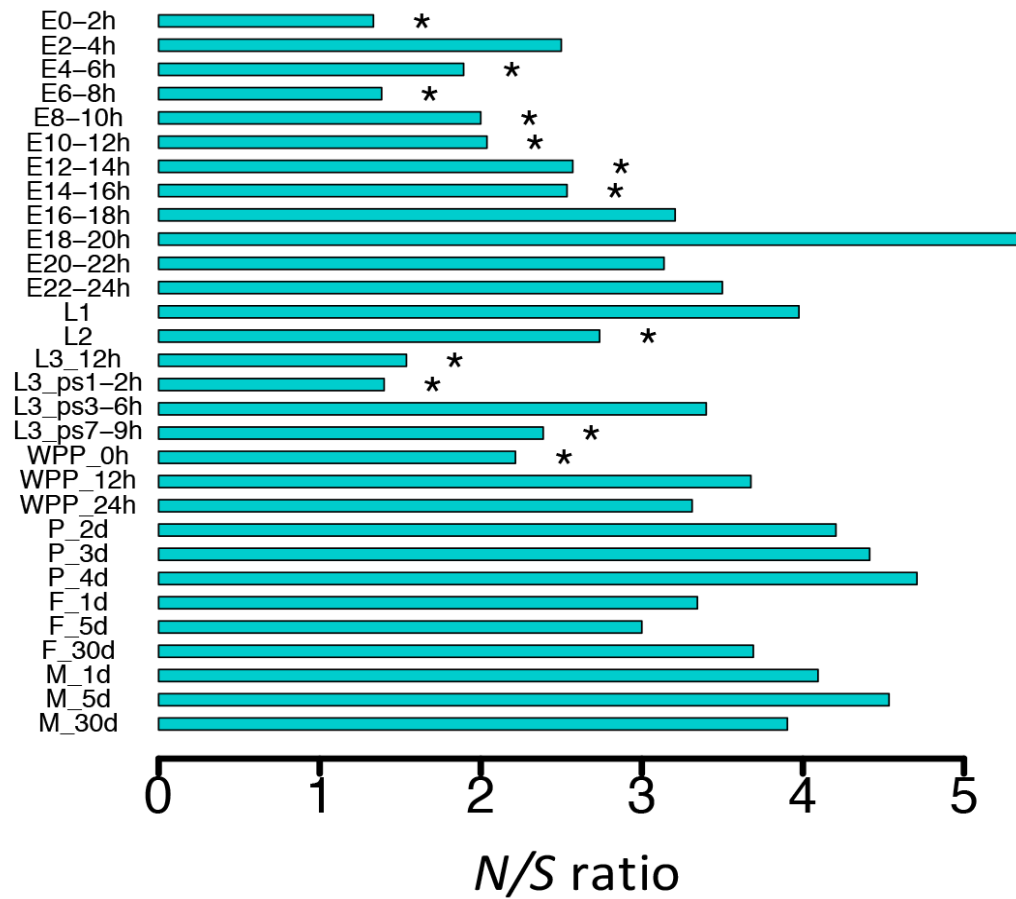

Supplement: S26 Fig — The N/S ratios (x-axis) for all the editing sites in different developmental stages of D. melanogaster in the modENCODE Project re-analyzed by Ramaswami et al. The overall observed N/S ratios are compared to the expected N/S ratio under neutral evolution (3.80): *, P < 0.05; **, P < 0.01; ***, P < 0.001. (PDF) [file pgen.1006648.s063.pdf]

## Highly-expressed genes

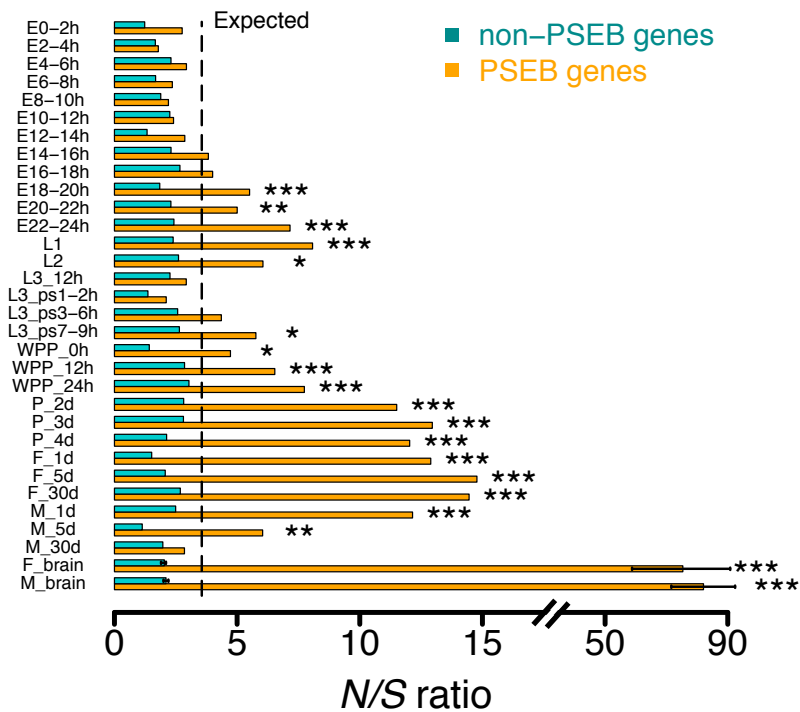

## Lowly-expressed genes

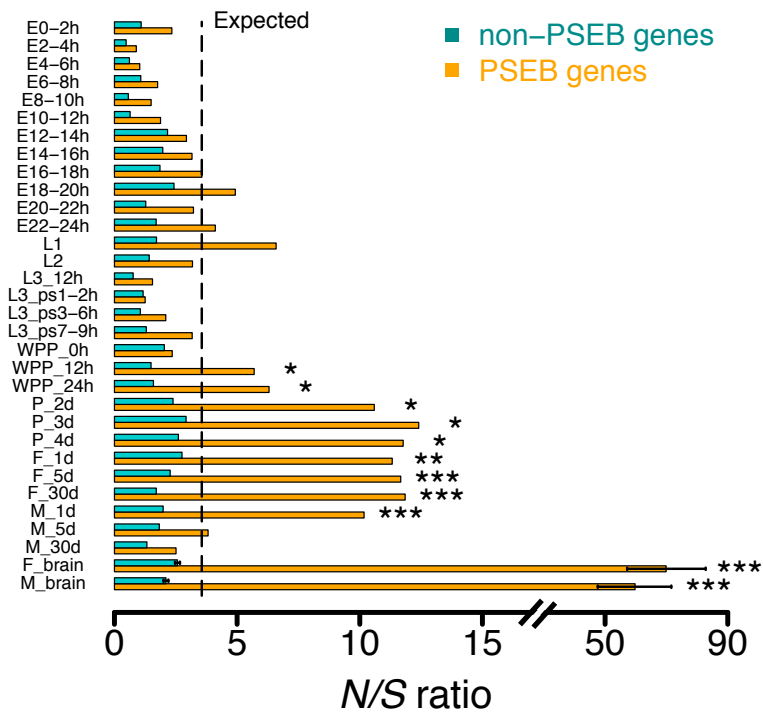

Supplement: S27 Fig — The N/S ratios (x-axis) for editing sites in PSEB genes (red) or non-PSEB genes (blue) that are Highly expressed (Left panel) or Lowly expressed (Right panel) in our brain data and different developmental stages of D. melanogaster in the modENCODE Project. Asterisks indicate significant difference in N/S ratios between PSEB and non-PSEB editing sites: *, P < 0.05; **, P < 0.01; ***, P < 0.001. (PDF) [file pgen.1006648.s064.pdf]

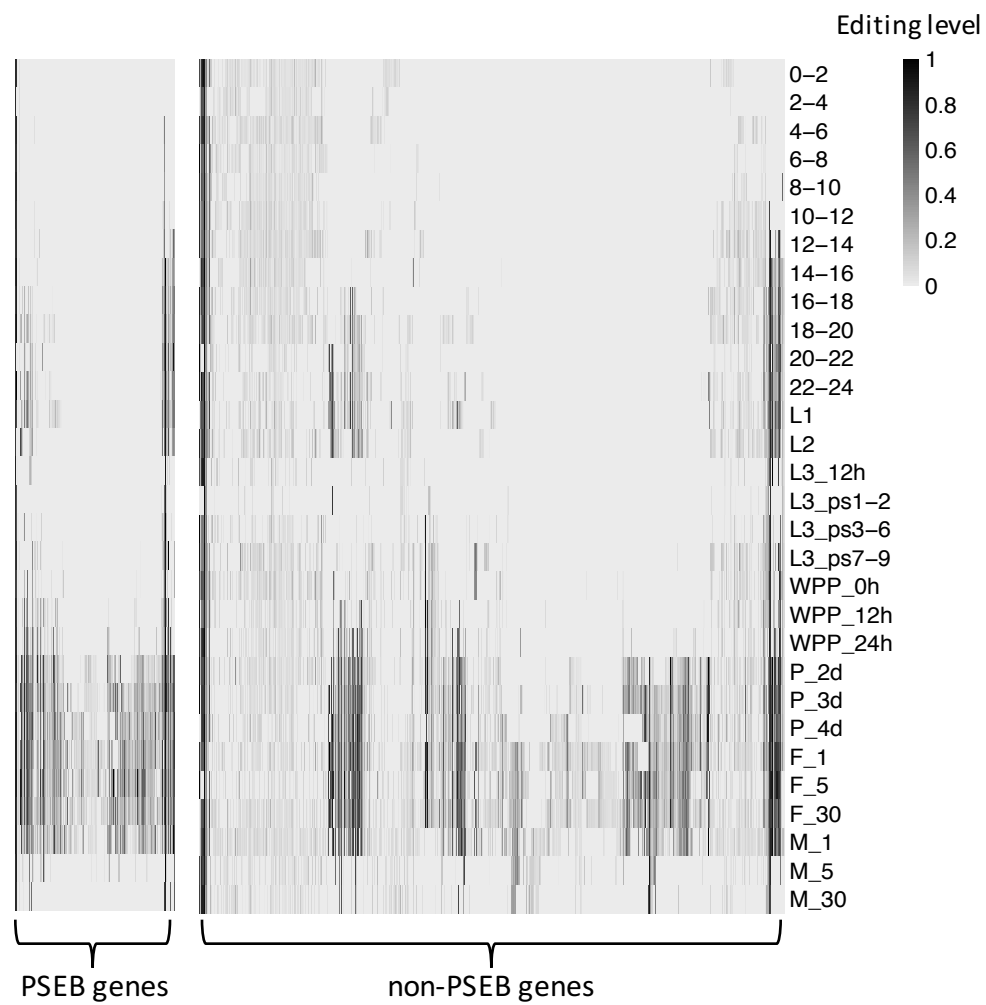

Supplement: S28 Fig — (PDF) [file pgen.1006648.s065.pdf]

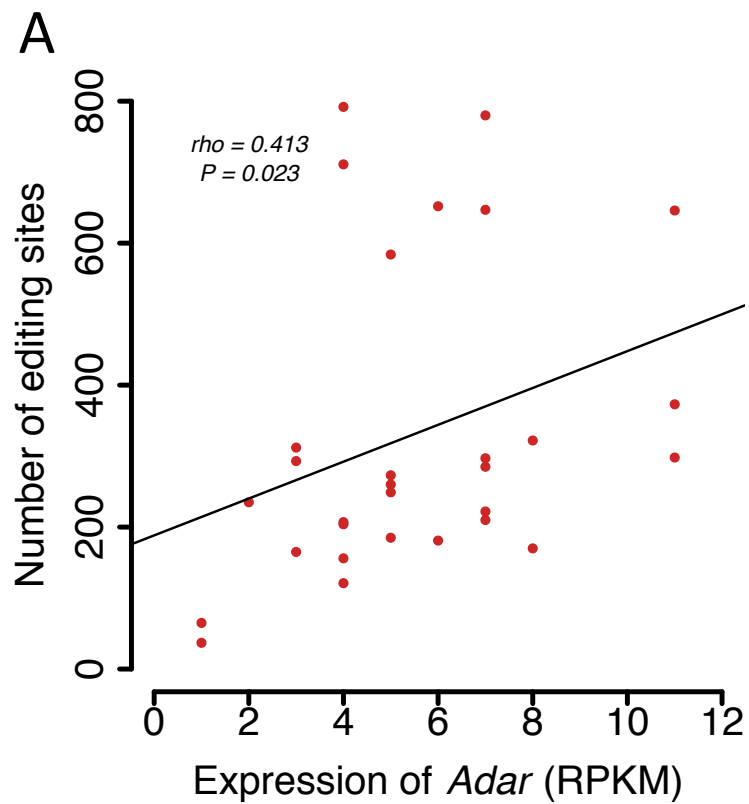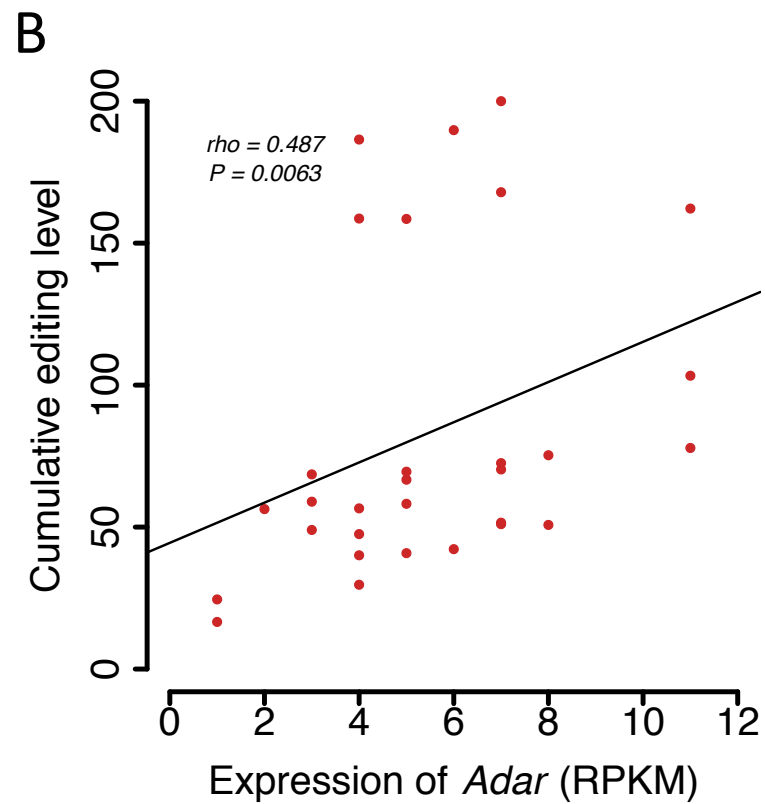

Supplement: S29 Fig — (A) The expression level of Adar is significantly positively correlated with the number of editing sites detected in our brain data and different developmental stages of D. melanogaster in the modENCODE Project. Spearman’s correlation coefficient rho was calculated and displayed in the plot. (B) The expression level of Adar is significantly positively correlated with the cumulative editing level of editing sites detected in our brain data and different developmental stages of D. melanogaster in the modENCODE Project. Spearman’s correlation coefficient rho was calculated and displayed in the plot. (PDF) [file pgen.1006648.s066.pdf]

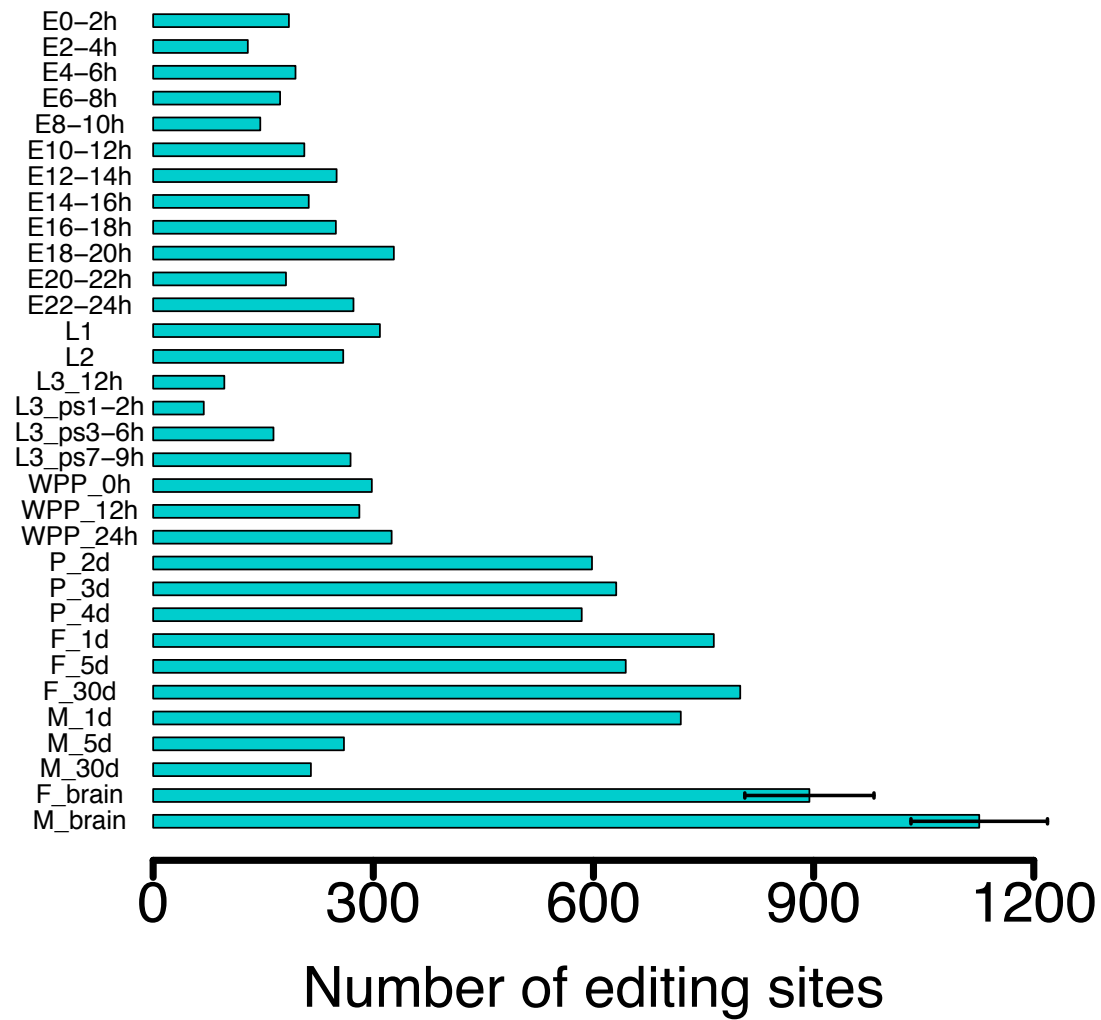

Supplement: S30 Fig — (PDF) [file pgen.1006648.s067.pdf]

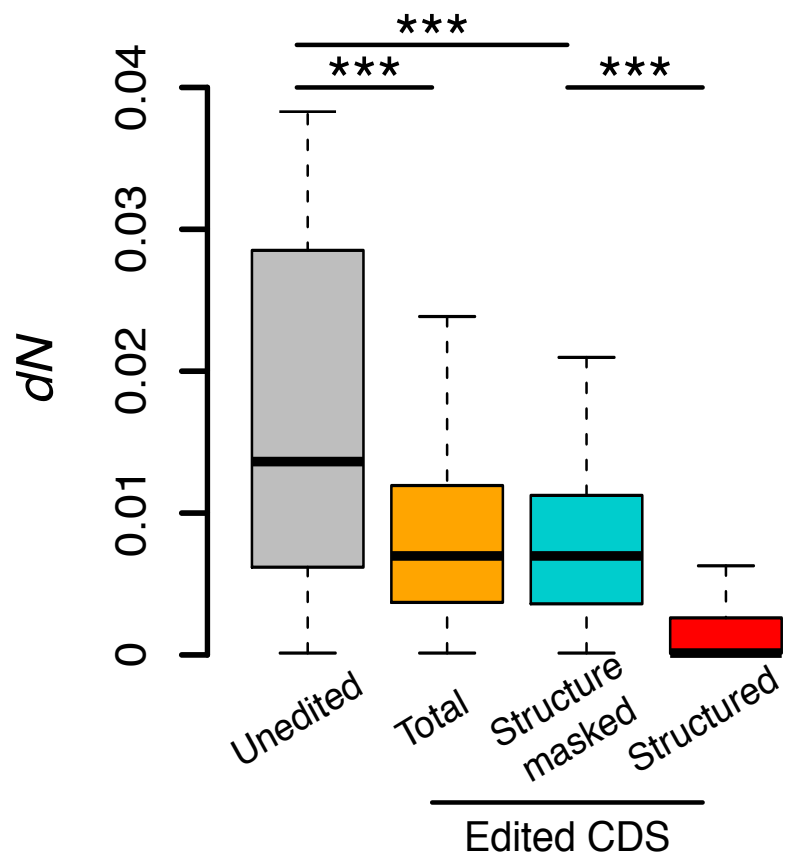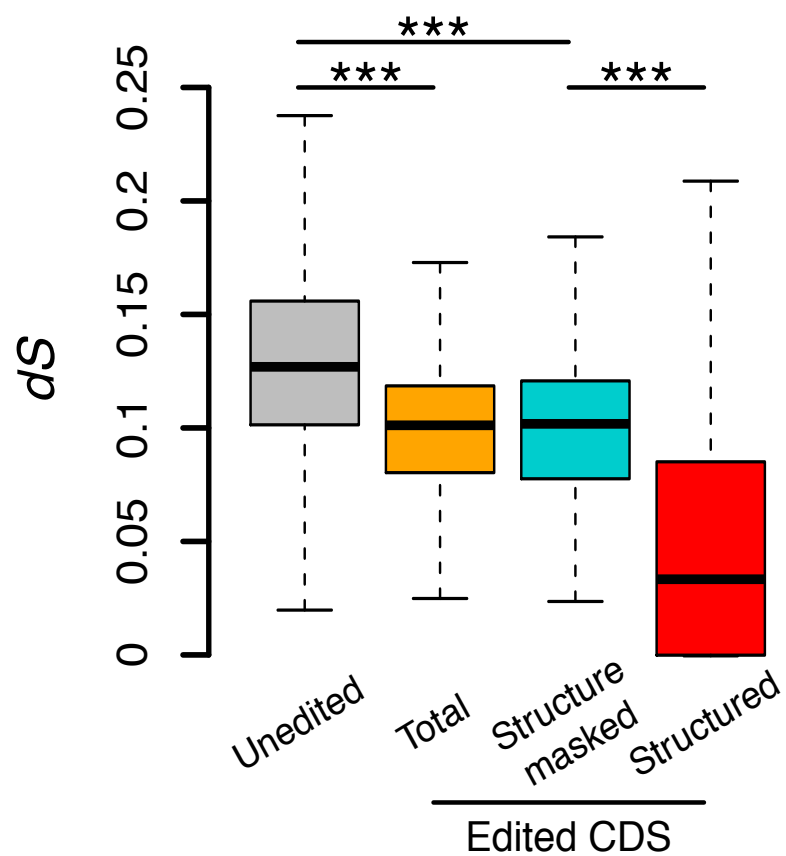

Supplement: S31 Fig — “Total” means the whole CDS regions; “Structured” means the CDS regions which form secondary structures and harbor editing events; and “Structure masked” means the remaining CDS regions which are outside secondary structures of mRNAs. (PDF) [file pgen.1006648.s068.pdf]
