# Supplementary material for: Adaptation of A-to-I RNA editing in Drosophila
Source: PLoS Genet. 2017 Mar 10;13(3):e1006648. doi: 10.1371/journal.pgen.1006648 (PMC5365144; doi:10.1371/journal.pgen.1006648)
Supplement: S5 Fig — (A) Nonsynonymous editing event (S>G) in Adar is located in a hairpin structure in D. melanogaster. The editing event was verified with Sanger sequencing of the female and male brains at 25°C and 30°C. Note that the editing level was reduced at 30°C in both the female and male brains. The nonsynonymous editing event (S>G) in Adar is located in a hairpin structure that is conserved in D. simulans and D. pseudoobscura. (B) A-to-I editing sites in the stable hairpin structures of the pre-mRNAs of rtp, DIP1, rdgA, CG43897 and CG42540. (C) Sanger verification of the editing events in the hairpin structure of rtp, DIP1, rdgA, CG43897 and CG42540. The editing sites are indicated by a blue arrow in the chromatograms above the Sanger traces. (D) A-to-I editing events in the 3' UTR of Adar are located in a long-range pseudoknot in D. melanogaster, D. simulans and D. pseudoobscura. Seven editing events in the 3' UTR of Adar are located in a long-range pseudoknot in D. melanogaster. Six of these events are evolutionarily conserved in the long-range pseudoknot in D. simulans, and five are evolutionarily conserved in a long-range pseudoknot in D. pseudoobscura. (PDF) [file pgen.1006648.s042.pdf]

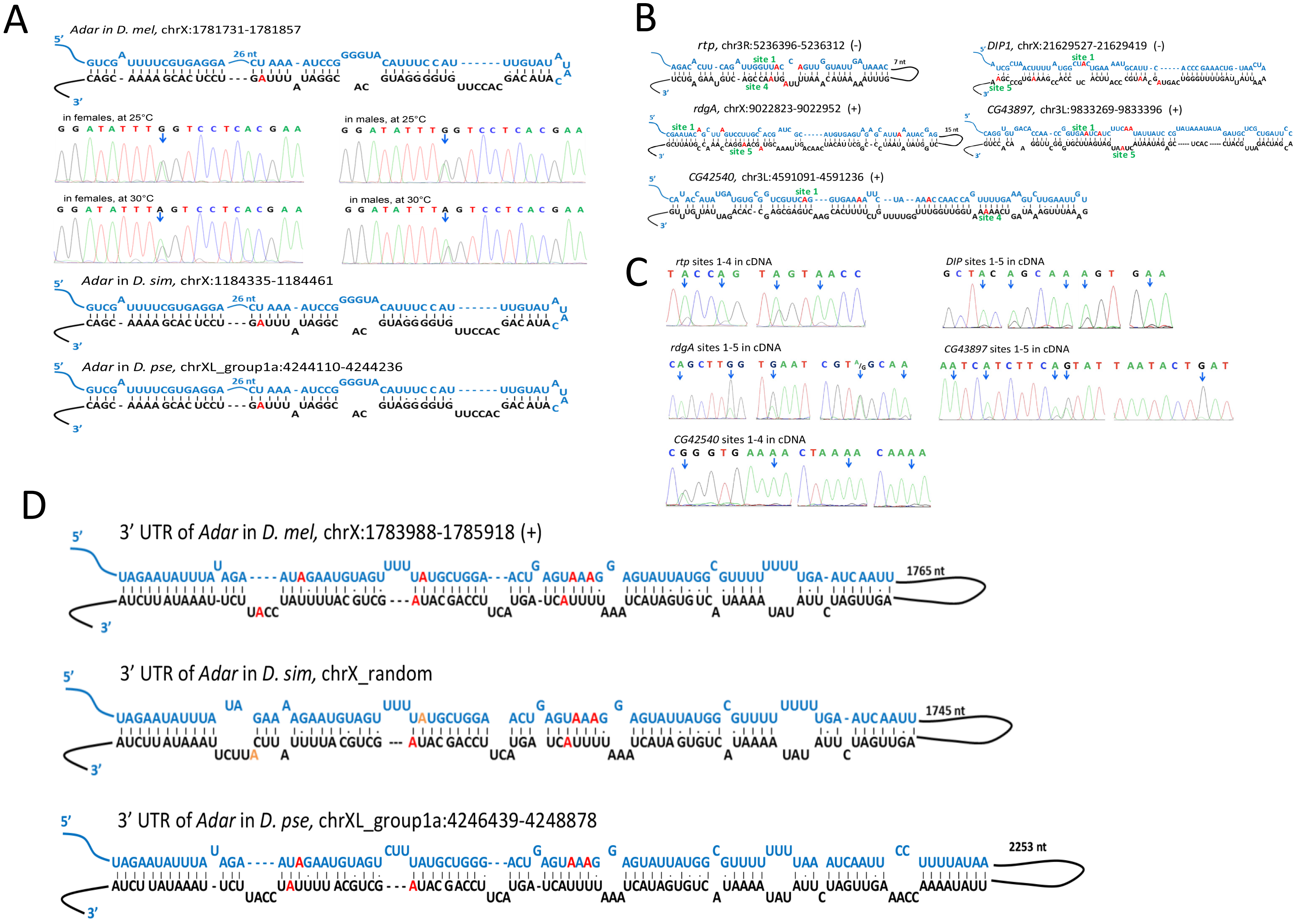
A
Adar in D. mel, chrX:1781731-1781857
in females, at 25°C
in males, at 25°C
in females, at 30°C
in males, at 30°C
Adar in D. sim, chrX:1184335-1184461
Adar in D. pse, chrXL_group1a:4244110-4244236
B
rtp, chr3R:5236396-5236312 (-)
DIP1, chrX:21629527-21629419 (-)
rdgA, chrX:9022823-9022952 (+)
CG43897, chr3L:9833269-9833396 (+)
CG42540, chr3L:4591091-4591236 (+)
C
rtp sites 1-4 in cDNA
DIP sites 1-5 in cDNA
rdgA sites 1-5 in cDNA
CG43897 sites 1-5 in cDNA
CG42540 sites 1-4 in cDNA
D
3' UTR of Adar in D. mel, chrX:1783988-1785918 (+)
1765 nt
3' UTR of Adar in D. sim, chrX_random
1745 nt
3' UTR of Adar in D. pse, chrXL_group1a:4246439-4248878
2253 nt
