# Supplementary material for: Adaptation of A-to-I RNA editing in Drosophila
Source: PLoS Genet. 2017 Mar 10;13(3):e1006648. doi: 10.1371/journal.pgen.1006648 (PMC5365144; doi:10.1371/journal.pgen.1006648)

A

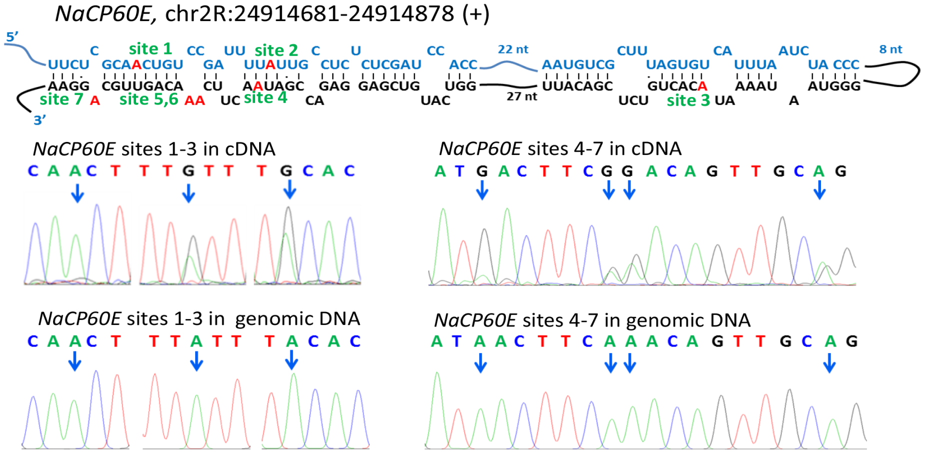

B

CaMKII, chr4:1041544-1041154 (-)

5’AGTTACTGGAAGGCGAACGAGAAATTTCATAGTTTTTTGGGCATATCGATAGACATTTACAAAACTAG
TAGAAAAGTATAAAAATATGTTTTAATAGTGTGGGTGTGGTAGTTTTGGGCAGCGTCTACGTCTCTGGA
ATCTGCTTGCCTAATCTCAACTTTCTATATTTTATTCTTCCTGAGATCTCGACCTTAACAAGGATGGACA
GACGGACGGACAGAAAGACATGGCCCGATCAACGTGGACCTGATCAAGGTGGTCGGAAACTCTTTCT
TCTATGCGATGCAATTTTTTCAACGCATCTAATATACTCTACTTTAGTCGACGAGTAACGAGTATAATTA
TTTCTATATGTATTTCTATGGTATTTCTATAGTCAAATACAGTATAA 3’

C

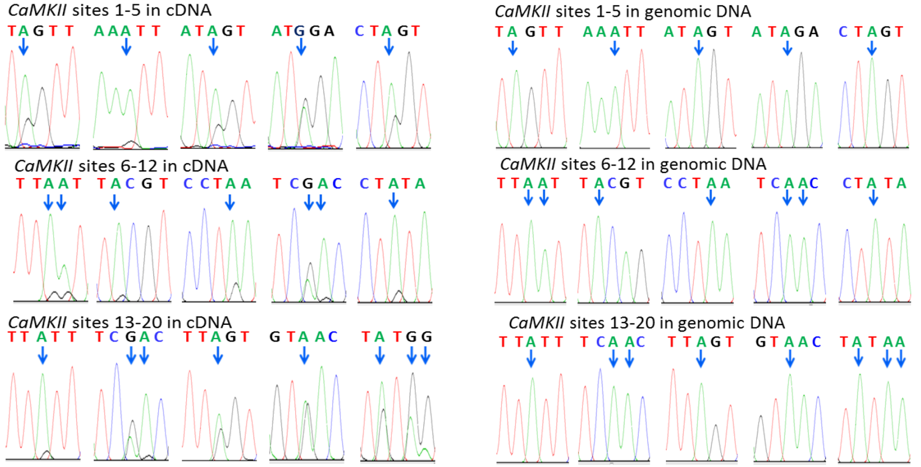

Supplement: S8 Fig — (A) Seven editing events are clustered in a hairpin structure in the CDS of NaCP60E. Editing sites are colored in red. All of these editing events were verified by Sanger sequencing the cDNA and genomic DNA. (B) Twenty editing events in the intron of CaMKII (editing sites are colored in red). (C) Verification of the 20 editing events in the intron of CaMKII by Sanger sequencing the cDNA (left) and genomic DNA (right). All of the editing events are indicated by a blue arrow in the chromatograms above the Sanger traces. (PDF) [file pgen.1006648.s045.pdf]
