# Supplementary material for: Adaptation of A-to-I RNA editing in Drosophila
Source: PLoS Genet. 2017 Mar 10;13(3):e1006648. doi: 10.1371/journal.pgen.1006648 (PMC5365144; doi:10.1371/journal.pgen.1006648)
Supplement: S14 Fig — Top: the x-axis is the cutoff of coverage (Cmin) and the y-axis is the simulated (median in black, and the range from 2.5% to 97.5% quantile is in blue) and observed (red) N/S ratio. The N/S ratio under neutral evolution (3.80) is indicated with dashed lines. Left: lmin = 0.01; Middle: lmin = 0.02; Right: lmin = 0.05. The corresponding relative differences (the simulated/observed N/S ratio) for each simulation is given at the bottom panel. (PDF) [file pgen.1006648.s051.pdf]

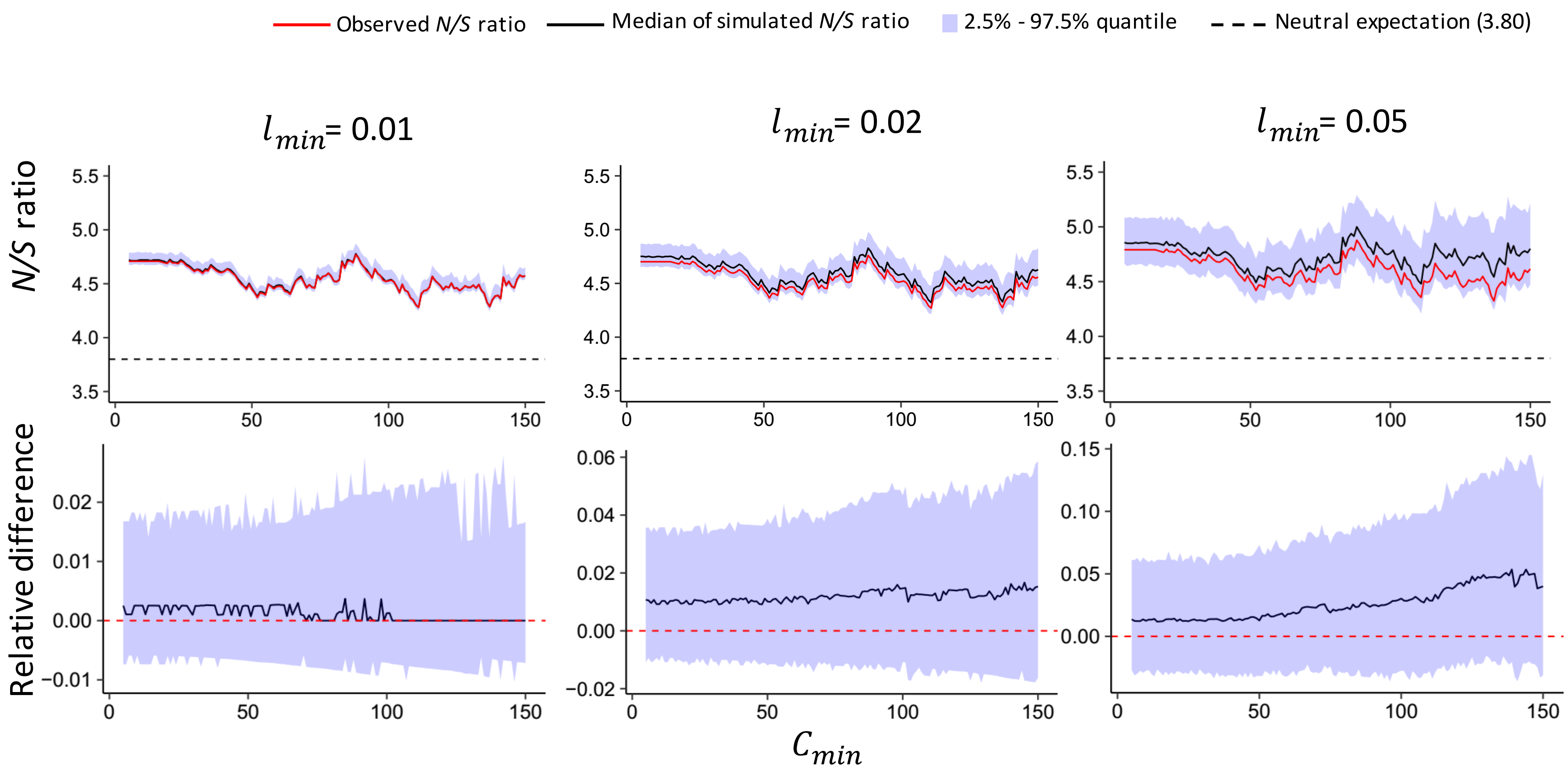
Observed N/S ratio
Median of simulated N/S ratio
2.5% - 97.5% quantile
Neutral expectation (3.80)
l_{min}= 0.01
l_{min}= 0.02
l_{min}= 0.05
N/S ratio
Relative difference
C_{min}
