# Supplementary material for: Adaptation of A-to-I RNA editing in Drosophila
Source: PLoS Genet. 2017 Mar 10;13(3):e1006648. doi: 10.1371/journal.pgen.1006648 (PMC5365144; doi:10.1371/journal.pgen.1006648)
Supplement: S15 Fig — (A) The observed N/S ratios for the X-linked and autosomal editing sites in all brain libraries of D. melanogaster. The expected N/S ratios for X chromosome and autosomes are presented in lines. For both X-linked and autosomal editing sites, the observed N/S ratios are significantly higher than neutral expectation (*, P < 0.05; * *, P< 0.01; ***, P < 0.001; Fisher’s exact tests). (B) N/S ratios for the X-linked and autosomal editing sites with events observed in brains of both D. simulans and the matched sample of D. melanogaster. The expected N/S ratios are presented in lines. For both X-linked and autosomal editing sites, the observed N/S ratios are significantly higher than neutral expectation (*, P < 0.05; * *, P< 0.01; ***, P < 0.001; Fisher’s exact tests). (PDF) [file pgen.1006648.s052.pdf]

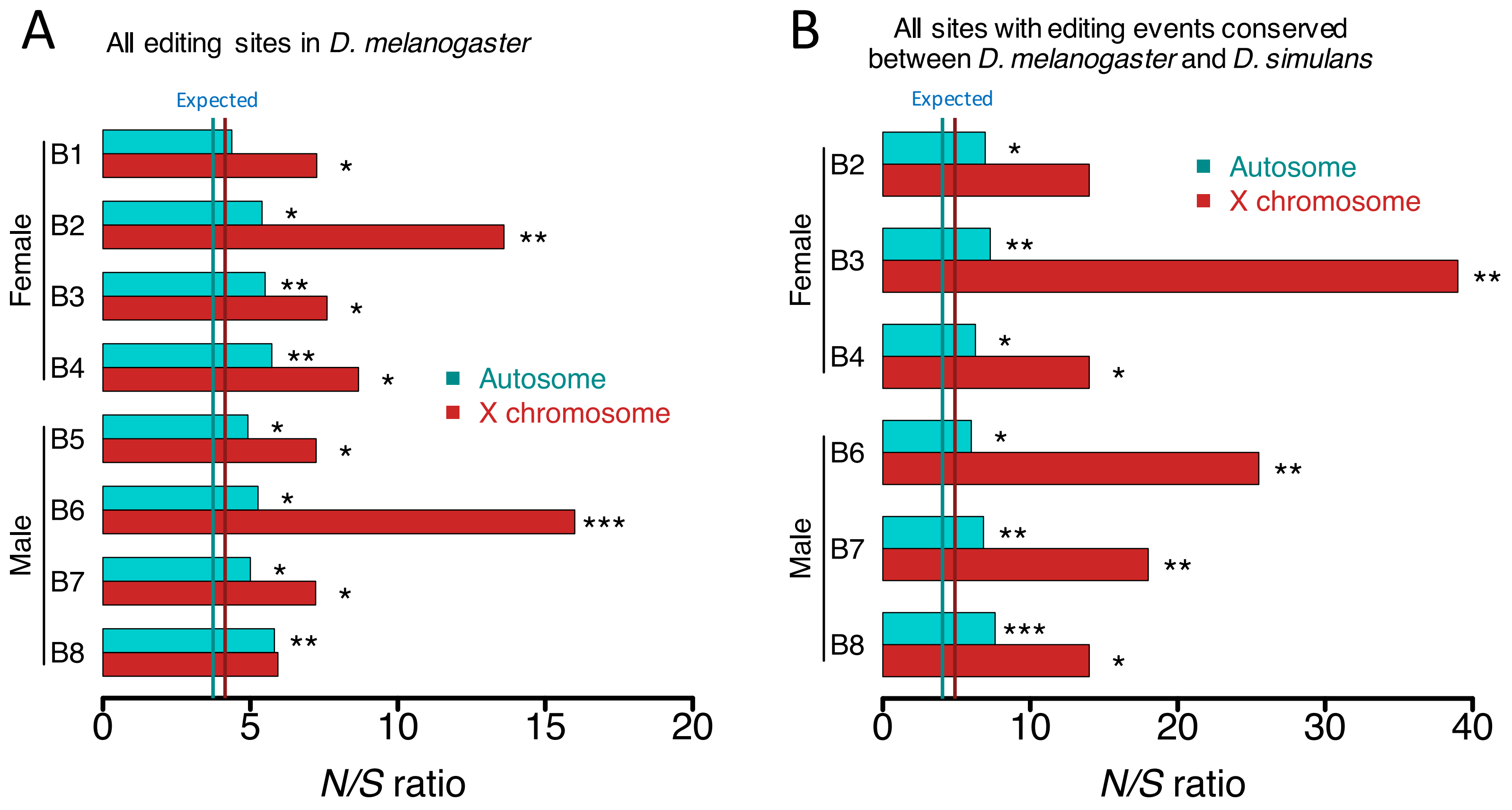

A
All editing sites in D. melanogaster
Expected
Female
Male
B1
B2
B3
B4
B5
B6
B7
B8
Autosome
X chromosome
0
5
10
15
20
N/S ratio
B
All sites with editing events conserved between D. melanogaster and D. simulans
Expected
Female
Male
B2
B3
B4
B6
B7
B8
Autosome
X chromosome
0
10
20
30
40
N/S ratio
