# Supplementary material for: Adaptation of A-to-I RNA editing in Drosophila
Source: PLoS Genet. 2017 Mar 10;13(3):e1006648. doi: 10.1371/journal.pgen.1006648 (PMC5365144; doi:10.1371/journal.pgen.1006648)
Supplement: S32 Fig — A-to-I RNA editing events in D. melanogaster that compensated for the G-to-A DNA mutation in the D. melanogaster lineage after splitting with its sibling species. The DNA sequences of D. simulans and D. yakuba are used as outgroups. (PDF) [file pgen.1006648.s069.pdf]

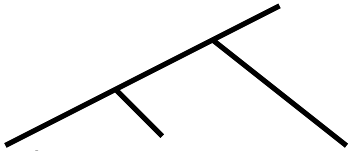

Sites	Gene	*D. mel*	*D. sim*	*D. yak*
chr2L:16263840, -	*crp*	A	G	G
chr2L:9790038, -	*CR18854*	A	G	G
chr3L:2562616, -	*msn*	A	G	G
chr3R:24512020, -	*nAChRbeta2*	A	G	G
chrX:19641592, -	*CG12237*	A	G	G
chrX:2112355, +	*csw*	A	G	G
chrX:3861324, -	*roX1*	A	G	G
chrX:3953264, +	*Vap-33A*	A	G	G
chrX:4659449, -	*CG3009*	A	G	G
